# Evolution of New *cis*-Regulatory Motifs Required for Cell-Specific Gene Expression in *Caenorhabditis*

**DOI:** 10.1371/journal.pgen.1006278

**Published:** 2016-09-02

**Authors:** Michalis Barkoulas, Amhed M. Vargas Velazquez, Alexandre E. Peluffo, Marie-Anne Félix

**Affiliations:** 1 Institute of Biology of the Ecole Normale Supérieure (IBENS), CNRS, Inserm, Paris, France; 2 Department of Life Sciences, Imperial College, London, United Kingdom; Harvard University, UNITED STATES

## Abstract

Patterning of *C*. *elegans* vulval cell fates relies on inductive signaling. In this induction event, a single cell, the gonadal anchor cell, secretes LIN-3/EGF and induces three out of six competent precursor cells to acquire a vulval fate. We previously showed that this developmental system is robust to a four-fold variation in *lin-3/EGF* genetic dose. Here using single-molecule FISH, we find that the mean level of expression of *lin-3* in the anchor cell is remarkably conserved. No change in *lin-3* expression level could be detected among *C*. *elegans* wild isolates and only a low level of change—less than 30%—in the *Caenorhabditis* genus and in *Oscheius tipulae*. In *C*. *elegans*, *lin-3* expression in the anchor cell is known to require three transcription factor binding sites, specifically two E-boxes and a nuclear-hormone-receptor (NHR) binding site. Mutation of any of these three elements in *C*. *elegans* results in a dramatic decrease in *lin-3* expression. Yet only a single E-box is found in the *Drosophilae* supergroup of *Caenorhabditis* species, including *C*. *angaria*, while the NHR-binding site likely only evolved at the base of the *Elegans* group. We find that a transgene from *C*. *angaria* bearing a single E-box is sufficient for normal expression in *C*. *elegans*. Even a short 58 bp *cis*-regulatory fragment from *C*. *angaria* with this single E-box is able to replace the three transcription factor binding sites at the endogenous *C*. *elegans lin-3* locus, resulting in the wild-type expression level. Thus, regulatory evolution occurring in *cis* within a 58 bp *lin-3* fragment, results in a strict requirement for the NHR binding site and a second E-box in *C*. *elegans*. This single-cell, single-molecule, quantitative and functional evo-devo study demonstrates that conserved expression levels can hide extensive change in *cis*-regulatory site requirements and highlights the evolution of new *cis*-regulatory elements required for cell-specific gene expression.

## Author Summary

Diversification of mechanisms regulating gene expression of key developmental factors is a major force in the evolution of development. However, in the past, comparisons of gene expression across different species have often been qualitative (i.e. ‘expression is on *versus* off’ in a certain cell) without precise quantification. New experimental methods now allow us to quantitatively compare the expression of gene homologs across species, with single cell resolution. Moreover, the development of genome editing tools enables the dissection of regulatory DNA sequences that drive gene expression. We use here a well-established “textbook” example of animal organogenesis in the microscopic nematode, *Caenorhabditis elegans*, focusing on the expression of *lin-3*, coding for the main inducer of the vulva, in a single cell called the anchor cell. We find that the *lin-3* expression level is remarkably conserved, with 20–25 messenger RNAs per anchor cell, in species that are molecularly as distant as fish and mammals. This conservation occurs despite substantial changes and compensation in the regulatory elements required for cell-specific gene expression.

## Introduction

Developmental systems operate in the presence of stochastic, environmental and genetic perturbations. While the output of a developmental system may be under stabilizing selection and remain mostly invariant, many internal variables such as the expression of a key gene or the activity of signalling pathways can be sensitive to perturbations. To reach a quantitative understanding of developmental systems, a key approach is to measure the sensitivity of the developmental system output to induced variation in an intermediate developmental phenotype. Whether and how this intermediate developmental phenotype varies within and among species then becomes a relevant evolutionary question [1]. The present work addresses the evolution of the expression level of the inducer of vulval development, *lin-3*, on which we previously performed a sensitivity analysis by manipulating its genetic dosage and addressing the phenotypic consequences for the developmental system [2].

The site and level of transcription of a gene can be modulated both in *cis* to the gene through *cis*-regulatory DNA sites directly influencing its transcription, or in *trans* due to evolution of trans-factors modifying the cellular context in which the gene is acting [3]. *cis*-regulatory sites containing binding sites for transcription factors often occur upstream of the coding region or in introns. These binding sites are often organized in modules, hence the designation as *cis*-regulatory modules (CRMs), acting in concert to enhance or repress gene expression in a given tissue at a given time. Changes in the number, relative order, orientation and spacing of transcription factor binding sites can affect transcription, often in a tissue-specific manner [4–6]. Tissue-specificity of CRMs is important for organismal evolution as it is thought to contribute to evolutionary novelty by minimizing pleiotropy [7–12]. Comparative studies in closely related species have revealed that transcriptional regulation can evolve through either extensive rewiring, or quantitative variation in the molecular components of a conserved network [11,13–17]. In particular, changes in *cis*-regulatory elements directly influencing the expression of critical developmental regulators have been shown to be a driving force for evolutionary innovation and phenotypic novelty in a variety of organisms. One example in *Caenorhabditis* concerns evolution between *C*. *elegans* and *C*. *briggsae* in the expression pattern of the transcription factor *lin-48* in the excretory system, resulting in a morphological change in excretory cell position. In this case, *lin-48* expression was gained in the excretory duct cell of *C*. *elegans* due to the acquisition of upstream binding sites for the transcription factor CES-2 [18,19].

Several features now make nematodes excellent experimental systems to understand gene expression evolution. First, rhabditid nematode species present a great advantage because homologous cells are easy to identify [20] so gene expression can be measured in a given cell. Second, the model organism *Caenorhabditis elegans* and other congeneric nematodes are amenable to functional genetics, transgenesis and now genome editing [21–26]. While transgenesis in *C*. *elegans* has long relied on formation of extra-chromosomal arrays containing many copies of the injected DNA that rearrange in an uncontrolled manner [27], the integration of a single copy at a defined locus is now possible, either at the endogenous locus using CRISPR/Cas9-mediated replacements [24–26,28] or at a controlled insertion locus using *Mos1*-mediated single-copy insertions (MosSCI) [29]. Third, *Caenorhabditis* species are highly divergent at the molecular level [30,31]. For example, *C*. *elegans* is as molecularly distant to *C*. *briggsae* as human is to mouse, and *C*. *angaria* as far as zebrafish to mouse [31], providing an opportunity to study the turnover of regulatory sequences at a large evolutionary scale where the nucleotide turnover is many times saturated yet the cellular context unchanged [32]. Many new *Caenorhabditis* species have recently been found and fully sequenced genomes are now available [33,34] (M. Blaxter, pers. comm.). Finally, the recent advent of quantitative methods, such as single-molecule fluorescent *in situ* hybridisation (smFISH) [35,36], allows to compare gene expression across species. The intensity of the conventional *in situ* hybridization signal cannot be meaningfully compared among species (regardless of whether the same probes or different probes targeting orthologs are used), while in the smFISH method the number of spots reflecting individual mRNA molecules can be counted, allowing a quantitative study of gene expression evolution.

**Fig 1:**
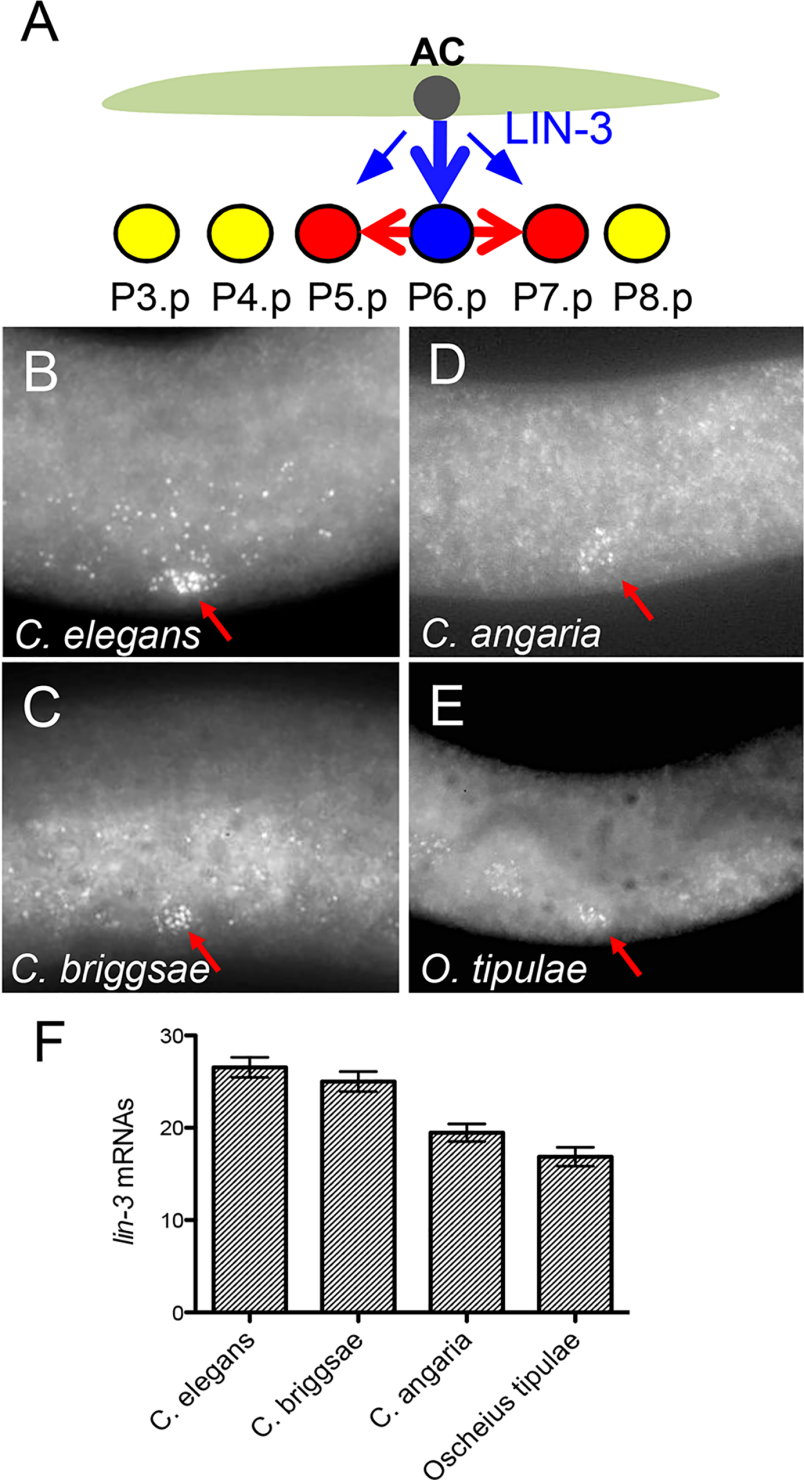
*lin-3* expression level in the anchor cell is overall conserved in different *Caenorhabditis* species. **(A)** Cartoon depicting the position of the anchor cell (AC) and Pn.p cells at the time of induction. Three Pn.p cells (P5.p –P7.p) are induced upon LIN-3 secretion. **(B-E)** smFISH using a *lin-3* probe in *C*. *elegans* N2 (data from [2]) **(B),** *C*. *briggsae* AF16 **(C)**, *C*. *angaria* RGD1 **(D)** and *O*. *tipulae* CEW1 **(E)**. Red arrow marks the position of the anchor cell. **(F)** Quantification of the number of spots detected in the anchor cell of these species at the time of induction (n = 32* animals for N2, n = 24 for AF16, n = 26 for RGD1 and n = 22 for CEW1). *: these include 20 animals from [2] (see Fig 6 for an independent dataset with a similar result). The difference between *C*. *elegans* and *C*. *briggsae* is not statistically significant with a Tukey’s multiple comparison test (P value = 0.99), whereas the difference between *C*. *elegans* and *C*. *angaria*, or *C*. *elegans* and *O*. *tipulae* is significant (P values < 0.0002).

Here, we take advantage of these recent developments to study the expression and requirement of *lin-3*, a model developmental gene involved in *C*. *elegans* vulval induction. The vulva is the egg-laying and copulatory organ of nematodes, and the *C*. *elegans* vulva is now a ‘textbook’ example of animal organogenesis [37]. *C*. *elegans* vulval development involves induction of three ventral epidermal cells (P5.p-P7.p) in response to the secretion of the LIN-3 signal from the anchor cell of the somatic gonad. LIN-3 activates the EGF receptor in the vulval precursor cells closest to the anchor cell and thereby acts as the upstream major inducer of vulval fates, in three precursor cells out of the six competent cells (Fig 1A). Induction of vulval fates involves interactions between EGF-Ras-MAPK, Notch and Wnt signalling, including some established pathway crosstalks [38]. We previously showed by modulating *lin-3* expression via single-copy transgenesis that the genomic level of *lin-3* expression is limited within a four-fold range for the vulva to develop normally in the *C*. *elegans* N2 background [2].

**Fig 2:**
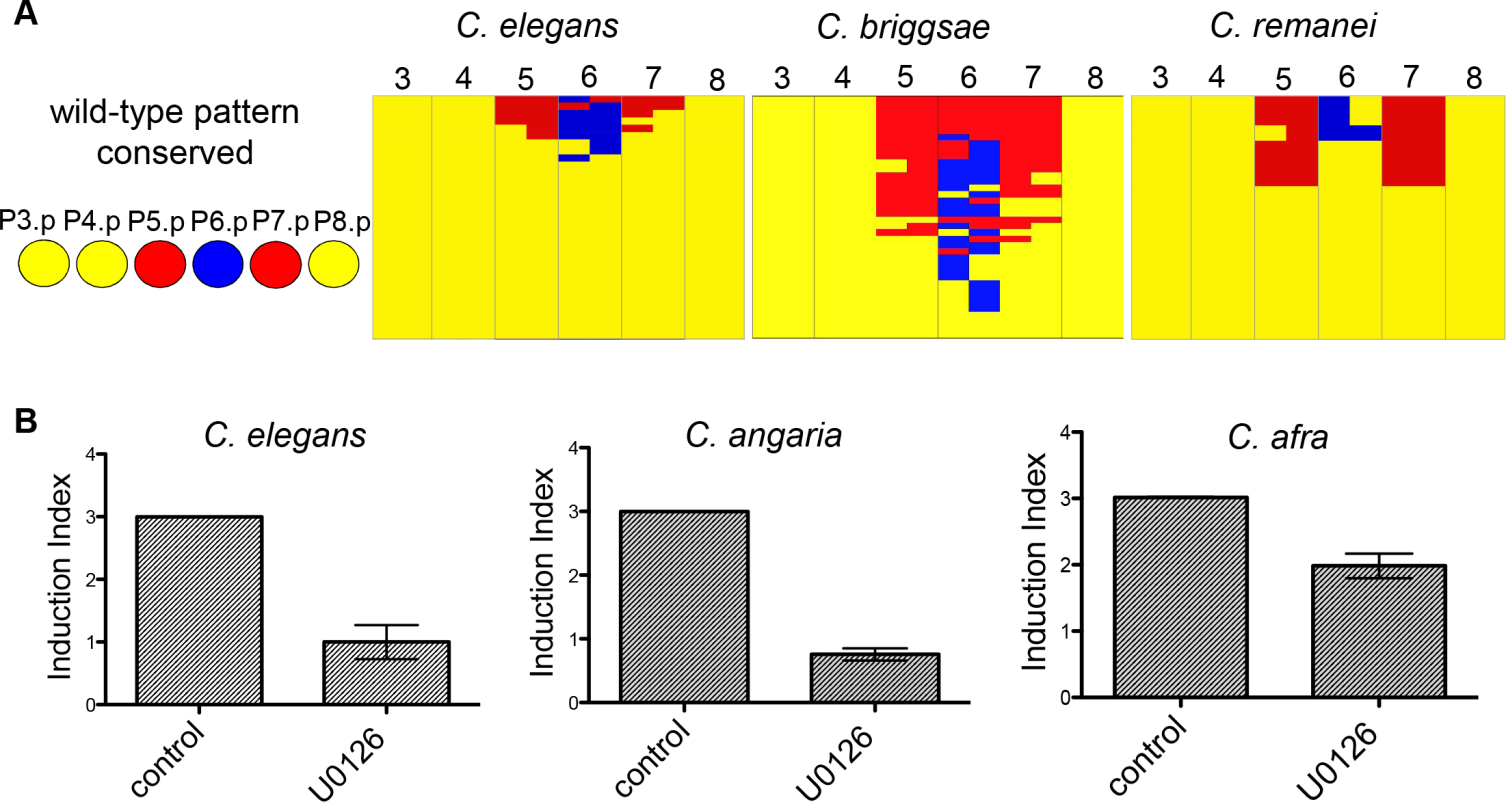
*lin-3* activity in vulval induction is conserved in *Caenorhabditis* species. **(A)** Comparative *lin-3* RNAi effect on vulva induction in *C*. *elegans*, *C*. *briggsae* and *C*. *remanei*. Tables show graphically the observed defects in vulval cell fate pattern after scoring at least 100 nematodes. Every column is a distinct Pn.p cell (3 to 8) and 1° fate is depicted in blue, 2° fate is depicted in red and 3° fate in yellow. Half fates represent cases where the Pn.p daughter cells adopt different cell fates after the first cell division. The defects are ordered based on their consequence on vulval induction index, from high index to low. **(B)** Treatment with the MEK inhibitor U0126 decreases vulval induction in *C*. *elegans* (n = 15 for DMSO control and 10 μM U0126 treatment), *C*. *angaria* (n = 32 for control, n = 27 for 150 μM U0126 treatment) and *C*. *afra* (n = 100 for control, n = 30 for 150 μM treatment*)*, as measured by the vulval induction index (average number of induced Pn.p cells at the population, wild-type index = 3). In all cases P<0.0001 with a Mann Whitney test.

The *C*. *elegans lin-3* gene has two alternative promoter regions, each including transcriptional and translational start sites. The *lin-3* anchor cell isoform is driven by a specific *cis*-regulatory module lying immediately 5' of the second promoter, which is located in the first intron of the mRNA driven by the upstream promoter. Within this region, a 59 bp element was shown to be sufficient to drive expression in the anchor cell, acting as a transcriptional enhancer if placed upstream of a minimal promoter [39]. Anchor cell expression was shown to rely on two types of transcription factor binding sites in this 59 bp element, conserved in *C*. *briggsae* [39] (Fig 2): an NHR-binding site and two E-boxes. The *lin-3(e1417)* mutation substitutes a single nucleotide within the NHR-binding site and results in a strong reduction of *lin-3* expression in the anchor cell [2,39]. This site can be bound *in vitro* by nuclear hormone receptors such as *C*. *elegans* NHR-25. The two E-boxes surround the NHR-binding site (E-box_L_ for left to the NHR and E-box_R_ for right), each consisting of the conserved sequence “CACCTG” but on opposite DNA strands to each other. When either of them is mutated in a *lin-3*::*GFP* transgene context, GFP expression in the anchor cell is strongly reduced [39]. We refer for simplicity to the ensemble of these three regulatory elements as the “regulatory triplet”.

We show here that a relative stability in *lin-3* mRNA expression in the anchor cell and conservation of LIN-3 vulval induction activity contrasts with the turnover of *cis-*regulatory binding sites at the *lin-3* locus. We show that the difference in requirement of regulatory elements for anchor cell expression is due to evolution in *cis* to the *lin-3* locus without a need to infer evolution in *trans*. This evolution in *cis* occurs in a very short 58bp region upstream of the *lin-3* vulval specific isoform. This study uncovers the evolution of new *cis*-regulatory motifs required for cell-specific gene expression.

## Results

### Evolutionary conservation of *lin-3* mRNA expression in the anchor cell of *Caenorhabditis* and *Oscheius*

To determine the level of intraspecific variation in *lin-3* expression, we quantified *lin-3* expression in different *C*. *elegans* wild isolates. In the reference strain N2, a mean level of 25.4 *lin-3* mRNA spots was detected using smFISH [2,40] (Fig 1B; S1A Table). We found that the mean and range of *lin-3* expression in the anchor cell at the time of vulval induction are comparable between the *C*. *elegans* reference strain N2 and the most genetically divergent *C*. *elegans* isolates such as DL238 and QX1211 (S1A Fig; S1A Table).

We further explored *lin-3* expression in different rhabditid species. First, we searched for the *lin-3* ortholog in other available genomes (S2 Fig). The LIN-3 proteins can be aligned along their whole length, with a conserved signal peptide, EGF and trans-membrane domains. Interestingly, the most conserved parts of the proteins are the N-terminal part following the signal peptide and the intracellular domain [41].

We designed smFISH probes for the *lin-3* gene of *C*. *briggsae*, *C*. *afra*, *C*. *angaria* and *Oscheius tipulae* and found that *lin-3* is expressed in a single cell within the somatic gonad, immediately dorsal to P6.p, which we identified by DAPI staining as the anchor cell (Fig 1C–1E; S1B Fig; S1B Table). Similar to *C*. *elegans*, we also detected *lin-3* expression at a lower level in the gonad outside the anchor cell and in the pharynx. We quantified fluorescent spots in the anchor cell and found no significant difference between *C*. *elegans* and *C*. *briggsae* (mean of 26.5±1 standard error in *C*. *elegans* vs. 25±1 in *C*. *briggsae*) (Fig 1F). In *C*. *angaria* and *O*. *tipulae*, we only found a small decrease compared to *C*. *elegans* (Fig 1F). Although *lin-3* was clearly detected in the anchor cell of *C*. *afra* (S1B Fig), the inferior quality of the hybridisation signal compared to the background did not allow us to quantify fluorescent spots in this species. We conclude that despite the great genetic distance between these nematodes [31], the mean number of *lin-3* mRNAs is remarkably conserved at least in *C*. *briggsae* and may only vary within a narrow range in *C*. *angaria* and *O*. *tipulae*.

### Conserved role of LIN-3 in inducing vulval cell fates

The vulval cell fate pattern is conserved throughout the Rhabditidae family, to which the *Caenorhabditis* and *Oscheius* genera belong [42], nevertheless molecular underpinnings of vulval induction in species other than *C*. *elegans* remain mostly unknown. *lin-3* RNAi experiments in *C*. *briggsae* so far produced a weak effect [43]. In *Pristionchus pacificus*, an outgroup and the only nematode species for which we currently have substantial molecular information related to vulval induction, vulval formation relies on Wnt signalling and is thought to be independent of the EGF pathway [44,45].

To address whether the *lin-3* homolog plays a functional role in vulval induction in different *Caenorhabditis* species, we used a combination of RNAi and pharmacological inhibition. First, we used recently established strains of *C*. *remanei* and *C*. *briggsae* that are rendered sensitve to RNAi administered by feeding due to the expression of the *C*. *elegans* intestinal transporter *sid-2* [21,46]. *lin-3* RNAi treatment in these *C*. *briggsae* and *C*. *remanei* strains resulted in substantial reduction in vulval induction (Fig 2A; S3A–S3D Fig). We observed vulval cell fate phenotypes upon *lin-3* RNAi that are not found in *C*. *elegans*, but are in keeping with published results revealing cryptic variation in vulval fate patterning following anchor cell laser ablations. Specifically, we found that P(5–7).p adopted a 2°-3°-2° cell fate pattern in *C*. *remanei* and a 2°-2°-2° pattern in *C*. *briggsae* [17,43]. Second, we used the MAP kinase (MEK) inhibitor U0126 that inhibits the downstream signalling events following EGF receptor activation. Application of this inhibitor has been previously shown to decrease vulval induction in *O*. *tipulae* [47]. Consistent with this result, we also obtained evidence for loss of overall vulval induction both in *C*. *angaria* and *C*. *afra* (Fig 1B; S3D Fig). Thus, we conclude that *lin-3* is expressed in the anchor cell and plays a conserved role in inducing vulval fates in the *Caenorhabditis* genus.

### The *lin-3* regulatory triplet evolved at the base of the *Elegans* species group

**Fig 3:**
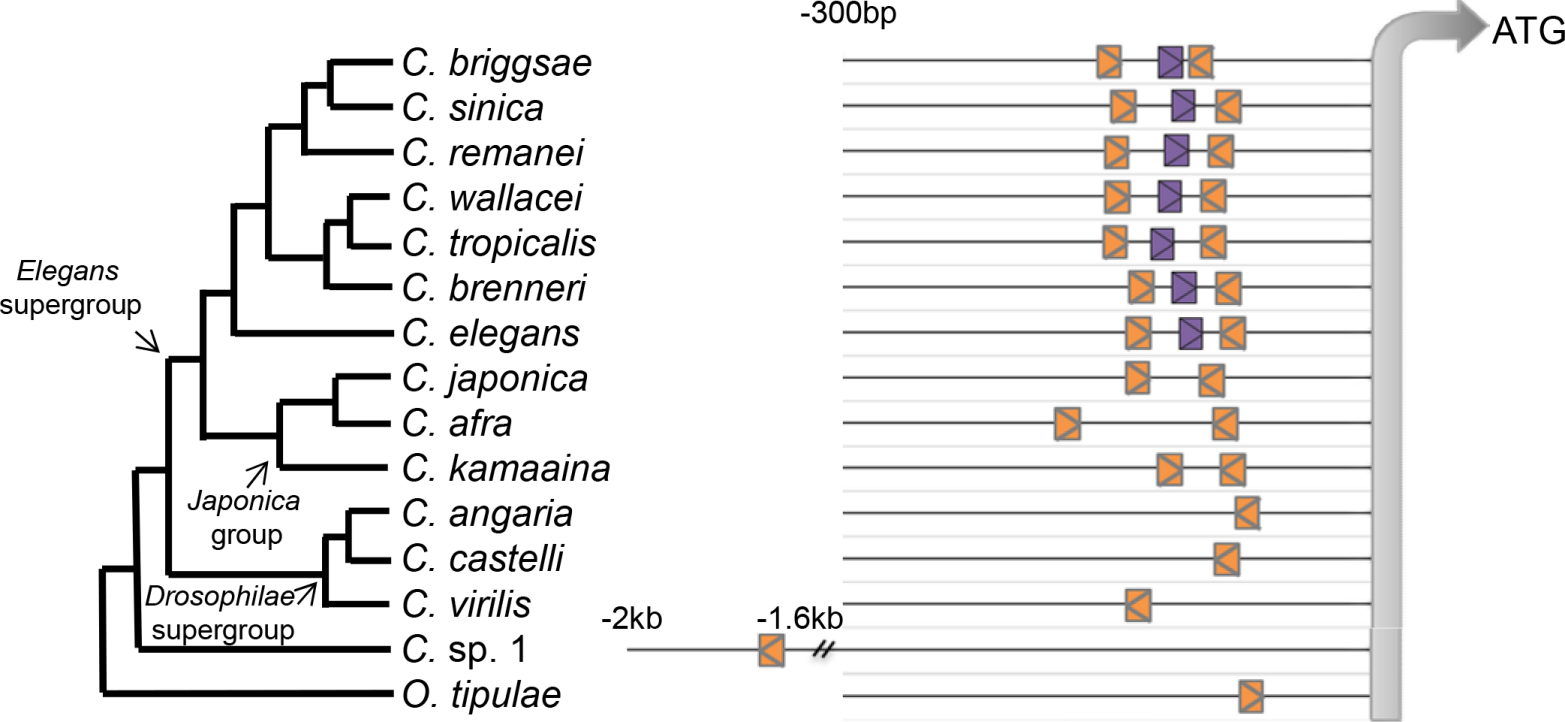
Evolution in the *cis*-regulatory elements necessary for *lin-3* expression in *C*. *elegans*. Distribution of *cis*-regulatory elements (the regulatory triplet) in different species. 300 bp upstream of the ATG of the vulval isoform of *lin-3* are shown (and up to 2 kb for *C*. sp. 1, where no upstream ATG is found). Orange depicts the E-box and purple the NHR site. “>” or “<” show orientation of the regulatory site.

Three transcription-factor binding sites, an NHR-binding site and two E-boxes, are required for *lin-3* expression in the anchor cell of *C*. *elegans* [39]. In light of the conserved expression pattern and level, we wondered whether these regulatory elements required for AC expression of *lin-3* are also conserved. The regulatory triplet was found to be present in different species of the *Elegans* group of *Caenorhabditis* including *C*. *briggsae* (Figs 3, S4 and S5). However, in the sister clade, called the *Japonica* group, we were able to find the two E-boxes, but no putative NHR-binding site within a window of 2.5 kb upstream of the translational start site of the vulval isoform of *lin-3*. In further outgroup species, such as *C*. *angaria*, we only found a single E-box, and no NHR-binding site in this region. One E-box within the *lin-3* CRM was also detected in the outgroup *Oscheius tipulae* (Fig 3). In *C*. sp. 1, we were able to detect a single ATG and the first E-box was only found 2 kb upstream. Overall, these observations suggest that the NHR-binding site was acquired in the branch leading to the *Elegans* group of the *Caenorhabditis* genus. The evolution of the second E-box at the base of the *Caenorhabditis* genus remains unclear: the second E-box may have been acquired in the branch leading to the *Elegans* supergroup or else be lost in the *Drosophilae* supergroup. No other sequence similarity could be found in the region upstream of the ATG of the vulva-expressed isoform of *lin-3* (S4 Fig).

The above results raised an interesting conundrum. How is it possible that some elements that are required for *lin-3* anchor cell expression in *C*. *elegans* are completely missing in related species, without any significant consequence for *lin-3* spatial and quantitative expression?

### A single *C*. *elegans* E-box cannot drive *lin-3* expression in the anchor cell

**Fig 4:**
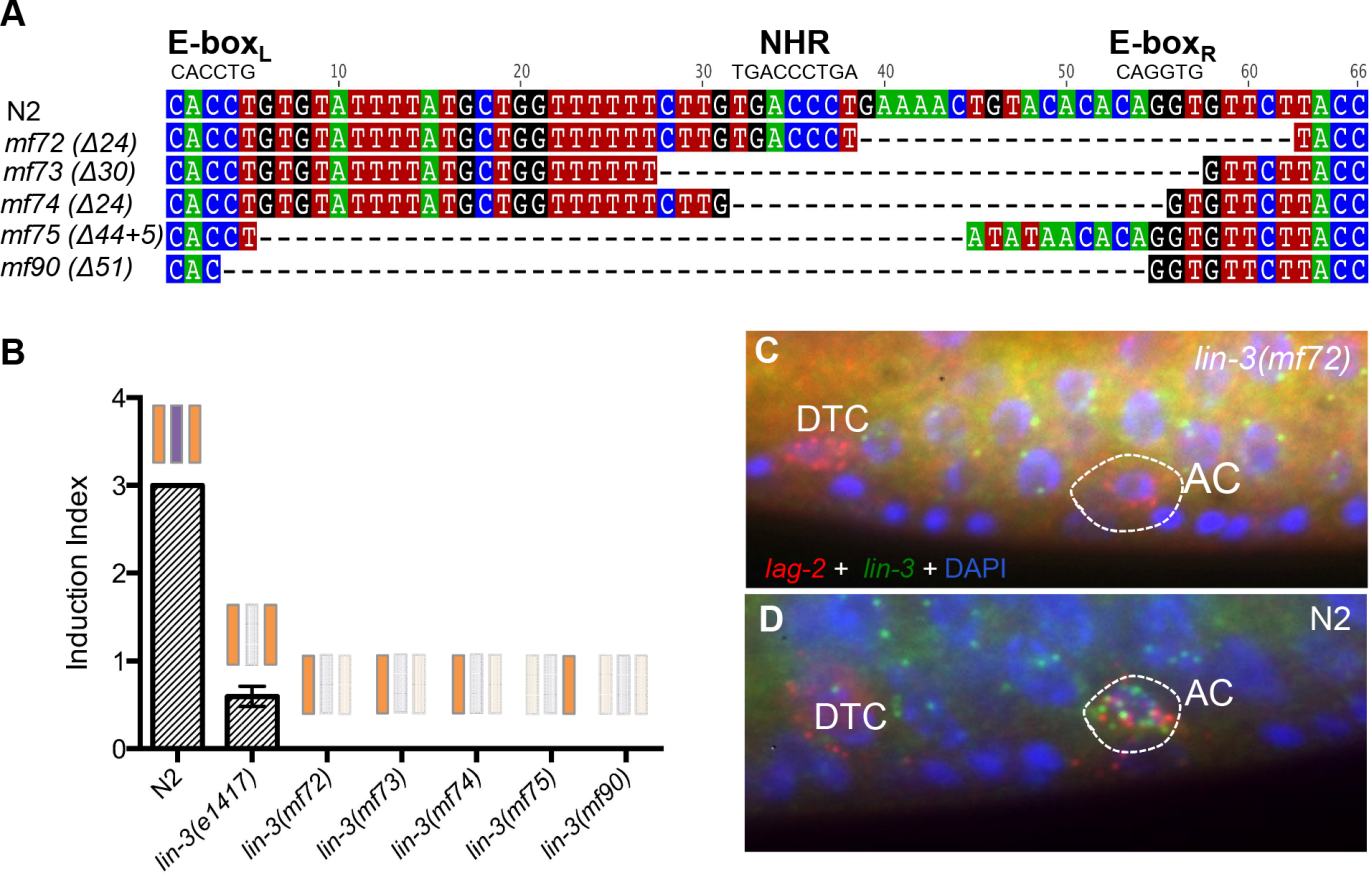
A single E-box in the *Cel-lin-3* CRM is not sufficient for *lin-3* expression in the anchor cell of *C*. *elegans*. **(A)** New *cis*-regulatory *lin-3* alleles with deleted E-box_L_ and NHR or NHR and E-box_R_. **(B)** Quantification of vulval induction in these new mutants. Note the complete absence of any induction in the recovered *lin-3* alleles (n>30). Scorings of *lin-3(1417)* animals are the same as those reported in Fig 5 and are used here to indicate that this mutation leads to vulval hypo-induction rather than no induction at all. **(C-D)** smFISH in *lin-3(mf72)* **(C)** and N2 **(D)** animals. Green spots correspond to *lin-3* transcripts and red spots to *lag-2* that is used as an anchor cell marker. Blue is DAPI staining of nuclei. Note the absence of *lin-3* expression in the anchor cell in the *lin-3(mf72)* mutant animal. Absence of *lin-3* signal in the anchor cell was also confirmed for the other *lin-3* alleles.

We first aimed to confirm that one E-box is not sufficient for *lin-3* expression in the anchor cell in *C*. *elegans*. We used CRISPR-mediated genome editing [48] to select deletions of *cis*-regulatory elements of the *C*. *elegans lin-3* gene. We generated a variety of alleles, in which either all three elements are deleted (*mf90*), or NHR and E-box_R_ are deleted leaving E-box_L_ intact (*mf72-mf74*) or only E-box_R_ is left intact (*mf75*), the latter recapitulating the cis-regulatory context of the *C*. *angaria lin-3* upstream module (Fig 4A). All these alleles result in fully penetrant vulvaless phenotypes with no cell induced to a vulval fate, thus a stronger phenotype than the *lin-3(e1417)* allele with one-nucleotide substitution in the NHR binding-site (Fig 4B). We used smFISH to detect *lin-3* transcripts and found no *lin-3* expression in the anchor cell, which was visualised by the unperturbed expression of *lag-2*. Interestingly, we still detected *lin-3* expression in the gonad of these mutant animals (Fig 4C and 4D). We conclude that these new *lin-3* alleles are anchor cell-specific null alleles.

These results confirmed that one E-box in the upstream cis-regulatory module of *lin-3* is not sufficient for *lin-3* expression in the anchor cell of *C*. *elegans*—whereas it appears sufficient in species of the *Drosophilae* group such as *C*. *angaria*.

### Compensatory evolution occurs in *cis* to the *lin-3* locus

The evolution in the requirement of transcription-factor binding sites for *lin-3* expression in the anchor cell could be due to changes in *cis* or in *trans* to the *lin-3* locus or both. We reasoned that if differences in *trans* were important, we would expect *lin-3* genomic fragments derived from species missing one or two *cis*-regulatory elements from the regulatory triplet to be unable to be expressed in the anchor cell of *C*. *elegans*. We tested this hypothesis and obtained multiple lines of evidence suggesting no role for changes in *trans* to the *lin-3* locus in explaining the differential binding site requirement.

First, we overexpressed in *C*. *elegans* a *C*. *angaria lin-3* genomic fragment containing 200 bp of upstream sequence, the coding region and the 3’ UTR. This fragment drove anchor cell expression of *Can-lin-3* and triggered vulval hyperinduction in *C*. *elegans*, further showing that the Can-LIN-3 protein could activate the *C*. *elegans* LET-23/EGF receptor (S6A and S6B Fig). Vulval hyperinduction was also observed when an equivalent genomic fragment from *C*. *elegans* was expressed in *C*. *angaria* or a fragment from *C*. *afra* was expressed in *C*. *elegans* (S6C and S6D Fig). These results indicate that the injected *lin-3* fragments from different *Caenorhabditis* species contain the necessary information for anchor cell-specific expression, despite the fact that a superficially equivalent *C*. *elegans* fragment with only one E-box, as in the new *lin-3* alleles described above, cannot be expressed in this cell.

**Fig 5:**
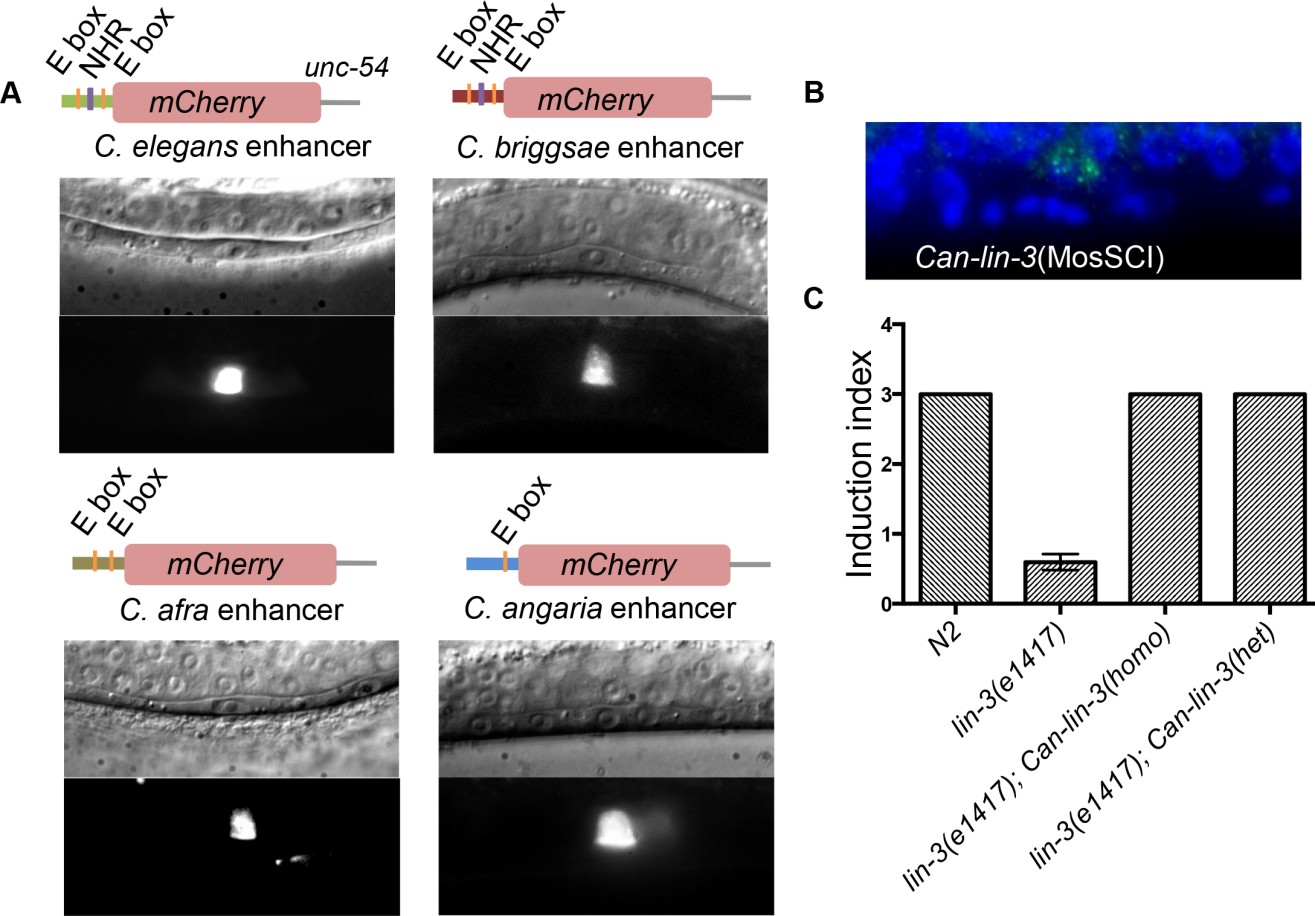
Evolution in *lin-3 cis*-element requirement does not result from a modified *trans* environment. **(A)** Transcriptional *lin-3-CRM*::*Cherry* fusions from different *Caenorhabditis* species are all expressed in the anchor cell of *C*. *elegans*. **(B)** A *Can-lin-3* single-copy transgene is expressed in the *C*. *elegans* anchor cell. smFISH detection of *Can-lin-3* in *C*. *elegans* strain harbouring a single extra-copy of *Can-lin-3*. Green spots correspond to *lin-3* expression, while blue is DAPI staining. **(C)** A single extra-copy of *Can-lin-3* fully rescues vulval induction of the *Cel-lin-3(e1417)* mutant to wild-type levels, both when homozygous (two copies, n = 100) or when hemizygous (one copy, n = 30). See the corresponding brood size rescue results in S8 Fig.

Since the regulatory triplet for *C*. *elegans* anchor cell expression is missing in these transgenes, we tested whether sequences in the introns, exons or 3'UTR sequences were required for expression of the *C*. *angaria* transgene in the anchor cell. To this end, we fused the *Can-lin-3* upstream sequences to a fragment containing the *C*. *briggsae lin-3* coding sequence and 3’ UTR. We expressed this fragment in *C*. *elegans* N2 and again observed clear expression in the anchor cell. As expected, in control injections containing only the promoterless *C*. *briggsae* fragment, the transgene was not expressed anywhere in the body (S7 Fig). To further strengthen these results, we fused the *lin-3 cis*-regulatory modules amplified from *C*. *elegans*, *C*. *briggsae*, *C*. *afra and C*. *angaria* to sequences encoding an unrelated protein, the fluorescent protein Cherry, and the unrelated *unc-54* 3'UTR. In all cases, we observed clear expression in the anchor cell (Fig 5A), indicating again that these short *cis*-regulatory modules alone contain the necessary information for anchor cell-specific expression in *C*. *elegans*. We conclude that evolution within the 200 bp upstream *cis*-regulatory module of *lin-3* is sufficient to explain the difference in requirement of regulatory elements for anchor cell expression within *Caenorhabditis*.

### The *C*. *angaria lin-3* transgene quantitatively mimics a *C*. *elegans lin-3* transgene

Above, we used multicopy transgenesis, which may cause sufficient expression and hyperinduction due to summing of weak transcriptional activity of many copies. We thus next asked whether the *C*. *angaria lin-3* fragment had quantitatively a similar activity to that of its *C*. *elegans* counterpart when introduced in single copy at a targeted genomic location outside the *lin-3* locus (using MosSCI transgenesis, see Methods). We found that a single-copy *Can-lin-3* insertion in *C*. *elegans* N2 is expressed in the anchor cell (Fig 5B) and does not cause hyperinduction, like an equivalent *Cel-lin-3* transgene copy [2]. Most interestingly, this single copy transgene could completely rescue the induction and brood size of *lin-3(e1417)* mutants, both in homozygous and hemizygous states (Figs 5C, S8). This quantitative behavior of the *Can-lin-3* transgene (rescue in the hemizygous and homozygous state, no effect when added to the endogenous locus) recapitulates the activity of a *C*. *elegans* copy inserted at the same genomic location [2]. This experiment shows that the *C*. *angaria lin-3* gene driven by its cis-regulatory element acts in a similar quantitative manner to the *C*. *elegans* fragment, even in the absence of the regulatory triplet.

### A 58 bp cis-regulatory fragment from *C*. *angaria* with a single E-box can replace the entire *C*. *elegans* regulatory triplet

**Fig 6:**
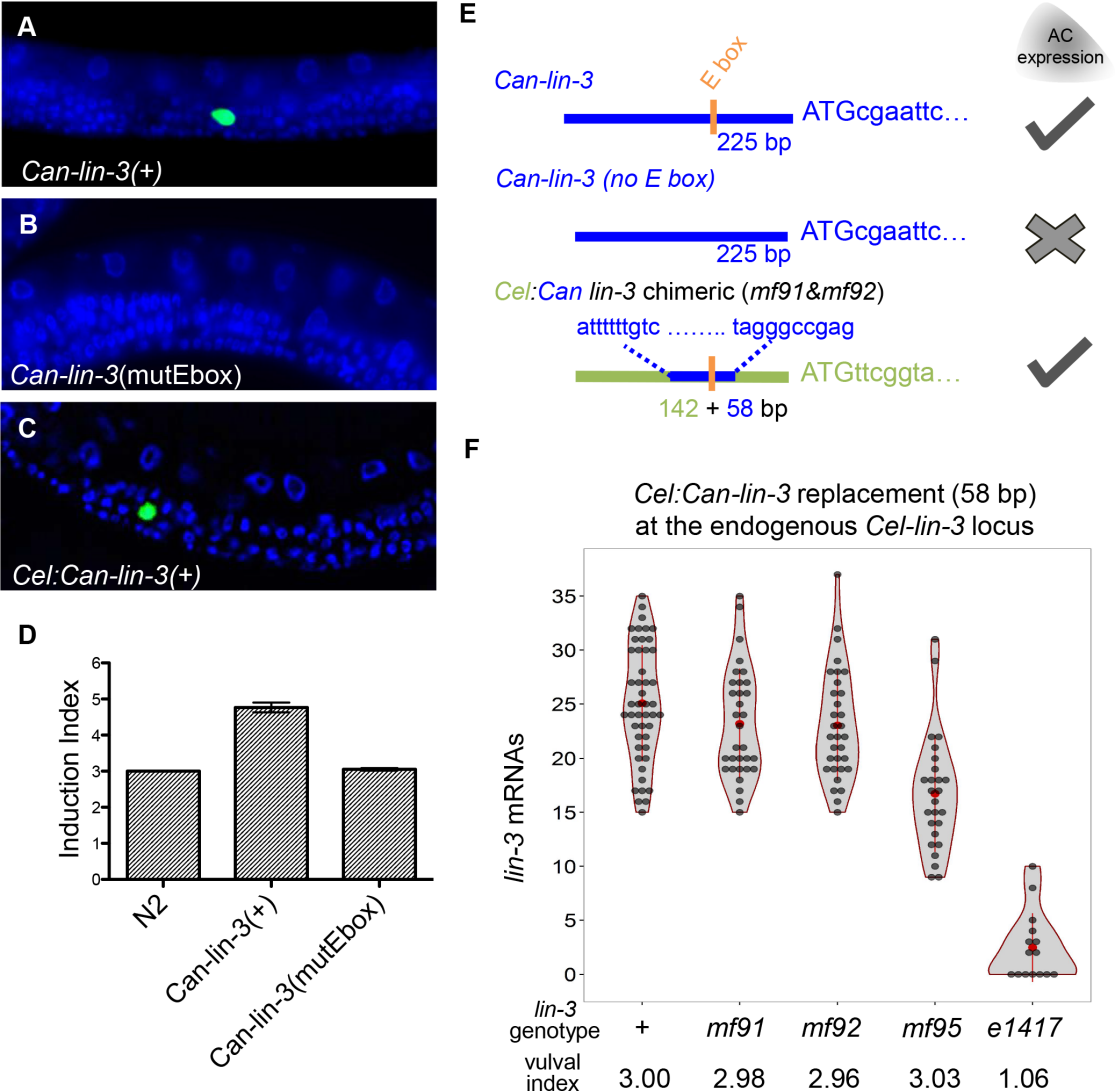
A 58 bp *C*. *angaria* fragment with a single E-box is able to replace the *C*. *elegans* regulatory triplet. **(A)** Expression of a *Can-lin-3* fragment in *C*. *elegans* containing the *Can-lin-3* CRM, coding sequences and 3’ UTR leads to *Can-lin-3* expression in the anchor cell, as detected by FISH. **(B)** Expression of the same fragment with a mutated E-box in the CRM results in loss of anchor cell expression. **(C)** A chimeric CRM that is mostly *C*. *elegans* apart from a 58 bp region around the regulatory triplet that is taken from *C*. *angaria* is also expressed in the anchor cell of *C*. *elegans*. (A) and (C) are using classical transgenesis in multicopy arrays experiments. **(D)** Over-expression of the *Can-lin-3(+)* fragment in *C*. *elegans* causes an increase in vulval induction, but not if the *Can-lin-3* fragment with a mutated E-box is used. **(E)** Summary of the compensatory changes in *cis* to the *C*. *angaria lin-3* locus allowing *lin-3* expression in the anchor cell. *C*. *elegans* sequences are depicted in green and *C*. *angaria* sequences in blue. Orange box corresponds to the E-box. **(F)** Modification at the *Cel-lin-3* endogenous locus, replacing the regulatory triplet with 58 bp from *C*. *angaria* containing a single canonical E-box with its genomic context (*mf91* and *mf92*) or with 7 bp modified to its right *(mf95* and *mf112)*. The violin plots show the number of *lin-3* mRNAs spots quantified in the anchor cell in the CRISPR replacements compared to N2 and *lin-3(e1417)* which are used as control strains. The whisker plot is superimposed in red. The average number of induced VPCs is shown below with the number of scored animals being 130, 159, 111, 42, and 35 from left to right.

To pin down the regulatory elements in the *C*. *angaria* transgene that are required for anchor cell expression, we mutated the E-box, which is the only distinguishable regulatory element in this short upstream region. We found that *Can-lin-3* genomic fragments with a mutated E-box lose their ability to be expressed in the anchor cell of *C*. *elegans* and to trigger vulval hyperinduction when expressed as multi-copy transgenes (Fig 6B, 6D and 6E). This shows that the single E-box_R_ of *C*. *angaria* is necessary for *lin-3* expression in the anchor cell of *C*. *elegans*.

Changes in the flanking sequences to core binding sites have been shown to contribute to binding efficiency of transcription factors, so we reasoned that perhaps the difference in requirement of regulatory elements for *lin-3* expression in the anchor cell may rely on nucleotides adjacent to the single E-box. To this end, we synthesised a chimeric CRM, where a 58 bp central portion harbouring the regulatory triplet in *C*. *elegans* was replaced with 58 bp from *C*. *angaria* containing E-box_R_ (Fig 6E). We first showed that this chimeric fragment can be expressed in the *C*. *elegans* anchor cell when used in multiple-copy extra-chromosomal array transgenesis (Fig 6C). Furthermore, we used genome editing at the *Cel-lin-3* locus to replace the endogenous *lin-3* CRM with this chimeric CRM. We found that the genome-edited animals expressed *lin-3* in the anchor cell at a normal level and produced a phenotypically wild-type vulva (Fig 6F; S2 Table).

These results demonstrate that the difference in requirement of *cis*-regulatory elements between *C*. *elegans* and *C*. *angaria* is explained by compensatory evolution within a very short cis-regulatory fragment (58 bp), rendering the presence of a second E-box and the NHR binding site unnecessary in *C*. *angaria*. Despite this loss of transcription factor binding sites, the activity of the cis-regulatory module in driving transcription in the anchor cell remains at the same quantitative level.

The compensation could be explained by the gain of new transcription factor binding sites in the *C*. *angaria* 58 bp regulatory region. To identify putative transcription factor binding sites, we performed a motif discovery approach in the anchor cell cis-regulatory *lin-3* regions of *Caenorhabditis* species close to *C*. *angaria* and an exhaustive search of transcription factors that could bind the 58 bp sequence (see Methods). We found the *GTTTATG* sequence, a possible Forkhead-binding site, to be significantly over-represented. This sequence is only one bp to the right of the *C*. *angaria* E-box. We tested whether modifying this sequence in the 58 bp *C*. *angaria* replacement would change the *lin-3* expression level. Indeed, when scrambling these 7 bp (see Methods; S2 Fig), *lin-3* expression was reduced significantly to about 60% of the wild-type level (*mf95* allele in Fig 6F; t-test, p-value < 6 10^−8^). However, as expected from a less than two-fold decrease [2], this new replacement, like the intact *C*. *angaria* CRM, produced phenotypically wild-type vulva cell fate induction (Fig 6F). Thus, we could affect the expression of the *C*. *angaria* CRM by modifying a motif adjacent to the E-box. This motif contributes to the compensation in *cis* in the 58 bp, but does not explain all of it, as *lin-3* expression in the *mf95* mutated replacement allele was still much higher than with a single *C*. *elegans* E-box.

## Discussion

### A quantitative account of gene expression evolution

This study addressed the level of expression of a critical developmental regulator in a single cell. We showed that both *lin-3* expression level in the anchor cell and its requirement for the induction of vulval cell fates are conserved in *Caenorhabditis* and *Oscheius* nematode species. We found that the mean *lin-3* mRNA level in the anchor cell only varies within 30%, despite the vast genetic divergence in this group—corresponding to that found among the most diverged vertebrates. We previously showed using quantitative perturbations that the mean level of *lin-3* expression in *C*. *elegans* needs to stay within a four-fold range for a correct vulva pattern to arise and that the mean *C*. *elegans* N2 level is in the very middle (on a log scale) of this permissible zone. Therefore, it is likely that stabilizing selection acting on vulva formation [49] leads to stasis both in *lin-3* expression level and in its effect on vulval induction.

By contrast with this evolutionary stasis in vulval pattern and in the *lin-3* mRNA level, we showed that this cell-specific level of *lin-3* expression involves substantial turnover of key *cis*-regulatory elements, namely the appearance of a novel binding site (NHR) and the turnover of a second copy of an existing binding site (E-box). Each of these elements is required for anchor cell expression in *C*. *elegans* yet is absent in some *Caenorhabditis* species. We further focused on the difference in requirement of *cis*-regulatory elements for *lin-3* expression between *C*. *elegans* and *C*. *angaria*. A 58 bp fragment from *C*. *angaria* with a single E-box can replace the three *C*. *elegans* binding sites, demonstrating that compensatory evolution within this short *cis*-regulatory fragment at the *lin-3* locus is sufficient to explain this difference in transcriptional regulation

Among evo-devo studies that center on comparisons of gene expression patterns and the evolution of *cis*-regulatory sequences, this is to our knowledge the first study taking advantage of the latest available capabilities to edit genomes and to quantify the level of mRNA expression at the single-cell level in a multicellular eukaryote.

### Turnover of transcription-factor binding sites

Gene expression may evolve due to changes in *cis* or in *trans* to a given locus, two possibilities that are not mutually exclusive. *Cis*-regulation may occur from sites quite distant to the transcriptional unit due to long-range chromatin interactions. Our data provided strong support for compensatory *cis*-changes, and this in a DNA fragment directly upstream of the translational start site of the vulva specific isoform of *lin-3*. We cannot exclude that some further *trans*-changes facilitate the difference in requirement of regulatory elements between the two species. However, the *cis*-regulatory changes that we uncovered in this work are at least sufficient to explain the difference in requirement of regulatory elements for anchor-cell-specific gene expression in *Caenorhabditis*.

We have narrowed down the compensatory changes that allow the *C*. *angaria lin-3* to be expressed in the anchor cell in a very short region of 58 bp. To explain the compensatory changes, we performed an exhaustive search of transcription factor binding sites and found a putative Forkhead binding site immediately adjacent to the E-box in *C*. *angaria* and absent from the replaced 58 bp region of *C*. *elegans*. Mutation of this site significantly lowered *lin-3* expression, but insufficiently to affect the vulval induction level and it thus only partially explained the compensatory evolution in *cis* (Fig 6E). We further note that, because this putative Forkhead binding site is immediately adjacent to the E-box, we cannot distinguish between two scenarios: a role for another specific transcription factor binding site versus an alteration of the affinity of the E-box itself. An alternative model would indeed be that compensation occurs through a stronger affinity of the E-box in the *C*. *angaria* regulatory region, while the *C*. *elegans* E-box is insufficient to drive expression. Such differences in affinity may arise from changes in the sequences flanking the core binding sites as it has been shown for bHLH factors binding to E-boxes [50,51]. Variation in the flanking sequences next to core transcription factor binding sites has also recently been shown to influence both the levels and sites of gene expression for another developmentally important gene [52]. We conclude that the *GTTTATG* sequence contributes to the compensation, but does not explain it entirely.

### Evolution of transcriptional regulation without change in gene expression

Here we described some evolution in *cis*-regulatory elements that occurs without consequences at the level of gene expression, as observed in many other genes and various groups of organisms [53–56]. This *cis*-regulatory element turnover in the absence of phenotypic consequence can be viewed as an extension to the notion of developmental systems drift, which posits that distinct molecular mechanisms may underlie the emergence of similar developmental phenotypes [57]. In a similar way, the conservation of gene expression pattern and level may depend on distinct molecular mechanisms due to the loss and gain of binding sites. Indeed, if the invariant output phenotype that we consider is *lin-3* expression level in the anchor cell, the molecular events leading to it, such as transcription factor binding, do vary in evolution.

The best-studied example for conservation of gene expression pattern despite turnover of *cis*-regulatory elements is the stripe 2 enhancer of the *Drosophila* pair-rule gene *even-skipped*. The minimal stripe 2 enhancer (*eve2)* in *D*. *melanogaster* is a DNA region of approximately 500 bp that consists of multiple binding sites for activators such as Bicoid and Hunchback and for repressors such as Giant and Krüppel: their combination allows a confined expression in the second stripe along the antero-posterior axis of the early *Drosophila* embryo [58]. Compared to the described *lin-3 cis*-regulatory module, the *eve2* stripe element involves more transcription-factor binding sites and results in expression in a group of cells (nuclei) rather than in a single cell. Similar to the *lin-3* CRM, the transcription-factor binding sites change in *Drosophila* species in a way that binding sites required for correct expression in *D*. *melanogaster* are absent in the stripe 2 element of other species, though without leading to alteration in the expression domain, due to compensatory *cis*-changes [53,59]. Here we went further in replacing the endogenous cis-regulatory sequences at the locus by those of a distant species, and show a quantitative rescue of gene expression and vulval induction.

One previous example in *C*. *elegans* of turnover of binding sites involves *lin-48* expression in hindgut cells, which is conserved between *C*. *elegans* and *C*. *briggsae* despite turnover of EGL-38 upstream response elements [60]. This turnover shows both similarities and differences to the described evolution of *lin-3 cis*-regulatory elements. The similarity is that there is an increase in the number of EGL-38 response elements in *C*. *elegans*. However, in the *lin-48* case, there is evolution towards redundancy because the gain in one EGL-38 response element decreases the reliance on the existing element for correct gene expression.

More recently, evolution of *cis*-regulatory elements between *C*. *elegans* and *C*. *briggsae* has been studied by placing exogenous *cis*-regulatory elements from *C*. *briggsae* into *C*. *elegans*. A main result over several genes whose expression is conserved between the two species is the appearance of ectopic gene expression domains in these transgenic experiments, implying evolution both in *cis* and in *trans* [61,62]. In one case, the ability of the *unc-47* proximal promoter from *C*. *briggsae* to drive ectopic expression in some *C*. *elegans* neurons was mapped next to a conserved *cis*-regulatory motif [61].

We note that the *C*. *angaria* fragment conveys the same level of transcriptional activity yet that a few vulval cell fate patterning "errors" occur in the replacement lines (Fig 6F). We observed both hypoinduced and hyperinduced variants in each of the two replacement lines (S2 Table), but the very low frequency of these variants make them difficult to study quantitatively. In the case of the *eve2* enhancer, the minimal stripe element is embedded within a larger region of approximately 800 bp, and these flanking sites contribute to robustness to some genetic and environmental perturbations [63]. In *Caenorhabditis*, the distal promoter of *unc-47*, although largely not conserved, is also important for robust gene expression, acting perhaps in a sequence-independent manner [64]. It remains unclear whether any regions within and/or outside the *lin-3* CRM can play a similar role in stabilizing expression of *lin-3* in *Caenorhabditis* to different perturbations.

### An evolutionary gain in binding site requirement

The distribution of *lin-3 cis*-regulatory elements in different *Caenorhabditis* nematodes and the mapping of changes on the phylogeny suggests as the most likely evolutionary scenario a gain of regulatory sites: the likely acquisition of an E-box before the common ancestor between the *Elegans* and *Japonica* groups and a gain of an NHR-binding site before the origin of the *Elegans* group. In addition, these sites not only appeared, but also became indispensable for *lin-3* anchor cell expression at least in *C*. *elegans*.

The acquisition of such new short regulatory motifs (6 bp) is easy and gains of regulatory motifs have been observed in other systems as well [65]. Given the high robustness of vulval development to several perturbations, the evolution towards a dependence on a higher number of sites for anchor cell expression is counter-intuitive and suggestive of evolution towards fragility. It is currently unclear what drove the evolution of these novel motifs with a conserved gene expression, whether selection or drift. Gains in interconnectedness between components of transcriptional networks may often occur non-adaptively, for example if they do not disrupt the underlying regulation [66]. Such gains can also be reshaped in equivalent network configurations and eventually become necessary depending on the evolution of the transcriptional network [67].

## Materials and Methods

### Nematode culture, genetics and pharmacology

A complete list of strains used in this study is presented in the supplement (S3 Table). All strains were maintained at 20°C and handled according to standard procedures [68]. We used the Bristol N2 strain as a reference *C*. *elegans* strain on standard NGM plates with OP50 as a food source. The U0126 treatments were performed by supplying the DMSO-dissolved inhibitor to NGM plates at a concentration between 10–150 μM and letting synchronised L2 stage nematodes develop into L4 larvae. Control treatments in this case were performed by growing nematodes on plates supplemented with DMSO only.

For the *Can-lin-3* rescue of the *C*. *elegans lin-3(e1417)* mutant, JU2495 hermaphrodites were crossed to JU2498 males and the F1 or F2 progeny were analysed for hemizygous or homozygous insertion phenotypic rescue, respectively.

### Identification of *lin-3* orthologs in *Caenorhabditis* genomes

The *lin-3* genomic sequences of the different species were accessed in WormBase (www.wormbase.org; version WS252) or from the Caenorhabditis Genomes Project by Mark Blaxter's laboratory (http://bang.bio.ed.ac.uk:4567) or from Matt Rockman’s laboratory. The *Oscheius tipulae* genome was sequenced and assembled as a collaborative effort between M. Blaxter's and our lab (Besnard, Kotsouvolos et al., in preparation) and is available (http://oscheius.bio.ed.ac.uk/). We first used the TBLASTN algorithm conditioning only to the most identical hits, favouring those with high similarity in the N-terminal part and signal peptide, and lower e-value. Afterwards, we proceeded to predict gene bodies in these contigs using FGNESH (http://www.softberry.com) with a hidden Markov model specific to *C*. *elegans*. Finally, manual curation and annotation of the *lin-3* sequences were performed using as a reference the amino-acid sequence of the closest available *lin-3* ortholog.

### Transcription-factor motif recognition in *lin-3* promoter sequences

To study the evolution of the regulatory triplet in the *Caenorhabditis* clade, we analysed the promoter regions upstream of the downstream ATG corresponding to the N-terminal exon homologous to that known to be expressed in the AC of *C*. *elegans* (S2 Fig). First, to address whether the *cis*-regulatory *C*. *elegans* NHR-binding sites and E-boxes were present in the other species, we performed a scan in the promoters with the position weight matrices of HLH-2 and NHR-proteins available in JASPAR [69] using *matrix-scan* [70] and a n = 2 Hidden Markov Model specific to *C*. *elegans* (Fig 3). Similarly, we looked in these regions for DNA patterns known to be binding sites of bHLH proteins [51] using the *dna-pattern* tool present in the RSAT suite [71]. Once we had the position of these sites across the promoter regions, we proceeded to plot their location using RSAT *feature-map* tool (S5 Fig).

Additionally, we looked for DNA motifs different from the *cis*-regulatory *C*. *elegans* NHR and E-boxes binding sites by performing a motif-discovery approach in *Caenorhabditis lin-3* promoters using the RSAT tool *oligo-analysis* [71]. The top over-represented words of length 6, 7 and 8 base pairs were compared to known motifs available in JASPAR. We thus identify the *GTTTATG* to the right of the E-box. Finally, to identify possible transcription factors acting on the AC *lin-3* expression in the 58 bp *C*. *angaria* fragment, we performed an exhaustive search of the full JASPAR motif repertoire in the 58 bp replaced sequence using RSAT *matrix-scan*. This search found the putative Forkhead-binding motif and a putative overlapping bZIP-binding motif (Fos/Jun repressors). The 7 bp modification in the *mf95* replacement also affected this predicted binding site of bZip transcription factors.

### Cloning

All *lin-3* CRMs reside directly upstream of the ATG of the vulval isoform of *lin-3*. To create the *lin-3 CRM*::*Cherry*::*unc-54* constructs, we used a three-fragment Gateway approach merging the *lin-3* CRMs cloned in pDONOR P4-P1R, the Cherry ORF cloned in pDONOR 221 and the unc-54 3’UTR cloned in pDONOR P2R-P3. All primer sequences containing attB4 forward and attB1 reverse recombination sites used to amplify the CRMs from gDNA from different species are shown in S4 Table.

*unc-54* 3’ UTR was amplified from N2 genomic DNA using primers unc-54attB2 and unc-54attB3. Worm-optimised Cherry was amplified from pAA64 using primers containing the attB1and attB2 sites. All constructs were injected at 10 ng/μl with *myo-2*::*GFP* as co-injection marker and pBluescript as carrier DNA.

To create the *Can-lin-3* insertion by MosSCI, we amplified a 2.9 kb *lin-3* fragment from *C*. *angaria* genomic DNA using primers Canlin-3AvrII and Canlin-3XhoI. The amplicon was cloned into pCFJ151 (chromosome II targeting vector) [29] as an AvrII/XhoI fragment. Injections and recovery of insertions were performed using the direct insertion protocol, as previously described.

To overexpress *lin-3* fragments in *C*. *elegans* or *C*. *angaria*, we amplified genomic fragments amplified from *C*. *elegans* (5.2 kb), *C*. *angaria* (3.2 kb) and *C*. *afra* (5.1 kb) using primer pairs RH9for/RH9rev, Canlin-3F2/Canlin-3R1 and Caflin-3oxF2/Caflin-3oxR1, respectively. The PCR products were injected directly (30 ng/μl) together with pBluescript as carrier and *myo-2*::*GFP* as co-injection marker.

To mutagenize the E-box in the *C*. *angaria lin-3* CRM, the above 3.2 kb fragment was cloned into pGEM-Teasy and the 5’-CAGGTG-3’ sequence was modified to 5’-CAGGAA-3’ using primers t211a_g212a/ t211a_g212a_anti and standard *in vitro* site directed mutagenesis.

The chimeric construct replacing a 58 bp region containing the *C*. *elegans* regulatory triplet (5’-cacctgtgtattttatgctggttttttcttgtgaccctgaaaactgtacacacaggtg-3’) with a similar in length sequence from *C*. *angaria* containing only one E-box (5’-attttttgtcaaagatttttcggcgccaggtgtgtttatgactcatgttagggccgag-3’) was synthesised by Genewiz. This construct was used as PCR template to permute 7 bp to the right of the *C*. *angaria* E-box (5’-CAGGTGtGTTTATG-3’ to 5’-CAGGTGtTTGGATT-3’).

The chimeric construct to drive *Cbr-lin-3* under the *C*. *angaria* CRM was built using fusion PCR. Briefly, the *Can-lin-3* CRM was amplified from *C*. *angaria* genomic DNA with primers Canlin-3 F2 and CaACFusion and the *Cbr-lin-3* region coding region and 3’ UTR from *C*. *briggsae* genomic DNA with primers Cbrlin-3F1 and Cbrlin-3R1. The two amplicons were then fused together using a third PCR reaction with primers Canlin-3F2 and Cbrlin-3R1. The final product was injected as a PCR fragment at 20 ng/μl concentration.

### Single molecule fluorescence *in situ* hybridization

smFISH was performed in synchronized populations of L3 stage animals using short fluorescently labelled oligos as probes, as previously described [2]. The animals were age-synchronized by bleaching, followed by hatching of embryos in M9 buffer. The L1 larvae were then placed onto culture plates with food until the L3 stage, as determined by Nomarski microscopy, and then fixed.

The *C*. *elegans lin-3* and *lag-2* probes have been previously described [2]. The low level of genetic divergence within *C*. *elegans* allowed us to detect fluorescent spots while using the same FISH probe as in the N2 strain.

For all other species we followed the same protocol as with *C*. *elegans* with the following two modifications to decrease the more pronounced background fluorescence. We used 20% formamide in the hybridisation and wash solutions and performed three washes post-hybridisation instead of two in *C*. *elegans*. Given that we are using different probes consisting of fewer oligos for the detection of *lin-3* transcripts in these species together with slightly more stringent hybridisation conditions, the observed difference in the number of fluorescence spots may thus even be due to technical rather than biological reasons. The sequences of the new *lin-3* probes can be found in S5 Table. The probes were labelled with Quasar 670 (Biosearch Technologies) and diluted to 100–200 nM for the overnight hybridisation.

### RNAi

RNAi was performed by feeding the animals with dsRNA-expressing bacteria, as previously described [2]. The *C*. *elegans lin-3* RNAi feeding clone used in this study is from the Ahringer RNAi library (Source Bioscience). A *Cre-lin-3* fragment was amplified using oligos Crelin-3RNAiF1 and Crelin-3RNAiR1 that contain an XhoI restriction site. The PCR product was cloned into L4440 as an XhoI fragment. To create the *C*. *briggsae lin-3* RNAi clone, a fragment was amplified using primers Cbrlin-3RNAiF1 and Cbrlin-3RNAiR1 and then cloned into pDONR 221 (Invitrogen) using attB1F and attB2R universal oligonucleotides. The *lin-3* fragment was sequence verified and transferred to a Gateway compatible L4440 plasmid. Both constructs were transformed into *E*. *coli* HT115 for use in *C*. *elegans* feeding.

### Phenotypic characterisation

To score the vulval cell fate pattern, nematodes were mounted with M9 on 3% agar pads containing 10 mM sodium azide and analysed under Nomarski optics. Standard criteria were used to infer cell fates based on the topology and number of cells at the L4 stage [43,72]. Half fates were assigned when two daughters of the Pn.p cells acquired distinct fates after the first cell division.

### Genome editing

We followed the CRISPR/Cas9 target design and used reagents as previously described [48]. We targeted the following region at the *C*. *elegans lin-3* CRM 5’-accctgaaaactgtacacacAGG-3’ with AGG representing the PAM motif. We replaced the *unc-119* target site under the pU6 promoter [48] with the *lin-3* target site using fusion PCR first with primers E-box2A gRNA-F/ U6prom HindIII and E-box2A gRNA-R/ oligos U6prom EcoRI F followed by amplification of the full sgRNA fragment with U6prom EcoRI F/ U6prom HindIII R. The only modification was that we did not clone the *lin-3* sgRNA in a vector but injected it directly as a PCR product (40 ng/μl, together with 40 ng/μl *eft-3*::*Cas-9* and *myo-2*::*GFP* as co-injection marker).

To replace the endogenous *lin-3 cis*-regulatory element of *C*. *elegans* by a 58 bp *lin-3* element from *C*. *angaria*, we first obtained a chimeric double-stranded DNA as homologous recombination template, using Gibson assembly of *C*. *elegans lin-3* promoter extremities with 58 bp of the *C*. *angaria lin-3* upstream sequence. In a similar fashion, we obtained a homologous recombination template identical to the previous but with modified bases next to the *C*. *angaria* E-box. Oligonucleotide sequences are found in S4 Table. *C*. *elegans* N2 animals were injected with a DNA mix containing the Peft-3::Cas9 plasmid, the pU6::dpy-10 sgRNA plasmid (co-CRISPR marker), the Ebox-2A sgRNA containing plasmid and the double-stranded DNA repair templates (independently), with final concentrations of 50, 40, 100, and 30 ng/μl, respectively. On plates with a high number of animals displaying the Dpy phenotype, the F1 progeny were singled, and their progeny screened by PCR. Broods from independent P0 animals were found positive and rendered homozygous (two independent lines for each replacement). Both replacements were confirmed by Sanger sequencing. The resulting lines were given allele names *mf91* and *mf92* for the first replacement, and *mf95* and *mf112* for the second one.

## Supporting Information

S1 Fig Single-molecule FISH of *lin-3* in *C*. *elegans* and *C*. *afra*. **(A)** smFISH quantification of *Cel-lin-3*. The level of *lin-3* expression in other *C*. *elegans* isolates is similar to that in the N2 reference strain (n≥14 animals; S1A Table. **(B)** smFISH localising *lin-3* transcripts in the anchor cell of *C*. *afra*. Serial optical sectioning through the anchor cell of a single animal showing *lin-3* fluorescent spots.
(TIF)

S2 Fig (Text file): *lin-3* sequences from the different *Caenorhabditis* species, *Oscheius tipulae* and the *Cel*:*Can-lin-3(mf91&mf92) and Cel*:Can*-lin-3(mf95) replacement, with annotations of *cis*-regulatory binding sites. The sequences of the enhancers used in Fig 5A are in bold. The endogenous 3’ UTR used for the overexpression experiments in Figs 5B, 6A–6D, S6 and S7 is underlined.
(DOC)

S3 Fig *lin-3* RNAi and MEK inhibitor treatment in different *Caenorhabditis* species. **(A-D)** Nomarski images of L4 stage animals upon *lin-3* RNAi **(A-C)** or MEK inhibitor (U0126) treatment **(D)**. **(A-C)** *lin-3* RNAi by feeding in *C*. *remanei* strain JU1184 results in 2°-3°-2° **(B)** or 3°-3°-3° **(C)** vulval cell fates for P(5–7).p compared to the 2°-1°-2° of the wild-type **(A)**. **(D)** Treatment with U0126 decreases vulval induction in *C*. *angaria* and *C*. *afra*. Note uninduced cells in both cases. The vulva in control L4 animals shows the typical “Christmas tree” morphology in all species.
(TIF)

S4 Fig *lin-3 cis*-regulatory sequence alignments in different *Caenorhabditis* species. **(A-C)** Alignment of the 300 bp region upstream of the *lin-3* ATG shows no other similarity in different species outside the E-box (B) and NHR (C) binding sites. Cbr = *C*. *briggsae*, Csi = *C*. *sinica*, Cre = *C*. *remanei*, Cwa = *C*. *wallacei*, Ctr = *C*. *tropicalis*, Cbn = *C*. *brenneri*, Cel = *C*. *elegans*, Cja = *C*. *japonica*, Caf = *C*. *afra*, Can = *C*. *angaria*. **(D)** Comparison of the *Drosophila* FushiTarazu/F1 (FTZ-F1) binding site, the NHR-binding site in wild-type *C*. *elegans* and *lin-3(e1417)* mutant. At least two nucleotide changes are required to align putative NHR binding sites from the *Japonica* group of the *Caenorhabditis* genus to the sequence in *C*. *elegans* and multiple changes are required for *C*. *angaria*.
(TIF)

S5 Fig Distribution of bHLH *cis*-regulatory binding sites upstream the AC’s specific *lin-3* TSS in *Caenorhabditis* species. Location of transcription factor binding sites belonging to the bHLH protein family (as described in [51]) across DNA sequences upstream the TSS of the vulval form of *lin-3* mRNA. The location of the NHR-binding site belonging to the *lin-3* regulatory triplet is also depicted. Only the first 500 bp before the ATG are displayed for most of the species, except for *C*. *virilis* (only 300 bp) and *C*. sp. 1 (up to 1.9 Kb).
(TIF)

S6 Fig Cross-species *lin-3* transgenesis. **(A**) Wild-type *C*. *elegans* vulval invagination in the L4 stage as seen by Nomarski optics. **(B)** Over-expression of *Can-lin-3(+)* in *C*. *elegans* via transgenesis with repeated extra-chromosomal arrays results in vulval hyper-induction, with several additional invaginations in the L4 stage (arrowheads). **(C)** Injection of a *Cel-lin-3* fragment in *C*. *angaria* leads to vulval hyperinduction. **(D)** Over-expression of a *Caf-lin-3* fragment in *C*. *elegans* with repeated extra-chromosomal arrays leads to vulval hyperinduction.
(TIF)

S7 Fig The *Can-lin-3* CRM is able to drive specific expression in the anchor cell in *C*. *elegans*. **(A)** A promoterless *C*. *briggsae* fragment introduced into *C*. *elegans* is not expressed in N2. **(B)** The same fragment under the *Can-lin-3* CRM drives expression in the anchor cell of N2, as monitored using *Cbr-lin-3* FISH. Green corresponds to *lin-3* expression and blue is DAPI staining of nuclei.
(TIF)

S8 Fig Rescue of brood size defects by a single-copy insertion of *Can-lin-3*, in the homozygous or hemizygous state. A single-copy insertion of *Can-lin-3(+)* rescues brood size defects of *lin-3(e1417)* mutants (n>15). Note that the presence of a *myo-2*::*GFP* transgene linked to *lin-3(e1417)* in the background enhances the *lin-3(e1417)* brood size defects and does not allow rescue to wild-type brood size. Vulval induction in this experiment is presented in Fig 5C.
(TIF)

S1 Table ***lin-3* mRNA single-molecule FISH quantification in the anchor cell in different *C*. *elegans* wild isolates (sheet A) and different *Caenorhabditis* species (sheet B).** Each entry is an individual animal. Mean and standard deviation (SD) are indicated at the bottom.
(XLSX)

S2 Table *lin-3* mRNA single-molecule FISH quantification in the anchor cell of different *C*. *elegans* genotypes differing in their *lin-3 cis*-regulatory region, and corresponding vulval indexes (last sheet). The genotype is indicated in the name of the sheet. Gonad size of each individual is given in pixels. Experimental batch is indicated in the "Batch" column.
(XLSX)

S3 Table Nematode strains used in this study. (XLSX)

S4 Table Sequences of oligonucleotides used in this study (except those for smFISH). (XLSX)

S5 Table Sequences of the oligonucleotides used as pools for smFISH experiments. (XLSX)
