# Supplementary figures and images for: Evolution of New cis-Regulatory Motifs Required for Cell-Specific Gene Expression in Caenorhabditis
Source: PLoS Genet. 2016 Sep 2;12(9):e1006278. doi: 10.1371/journal.pgen.1006278 (PMC5010242; doi:10.1371/journal.pgen.1006278)

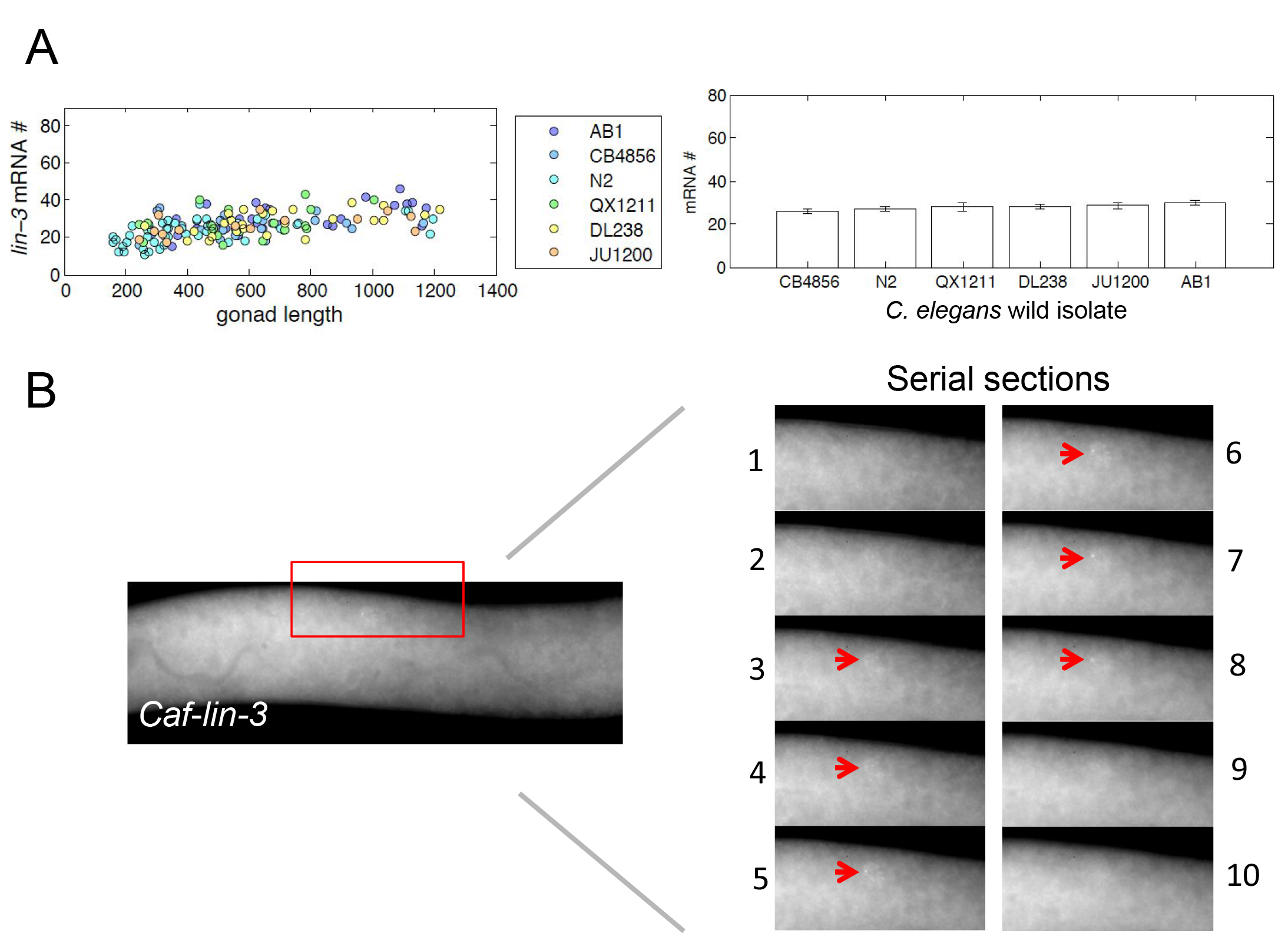

Supplement: S1 Fig — (A) smFISH quantification of Cel-lin-3. The level of lin-3 expression in other C. elegans isolates is similar to that in the N2 reference strain (n≥14 animals; S1A Table. (B) smFISH localising lin-3 transcripts in the anchor cell of C. afra. Serial optical sectioning through the anchor cell of a single animal showing lin-3 fluorescent spots. (TIF) [file pgen.1006278.s001.tif]

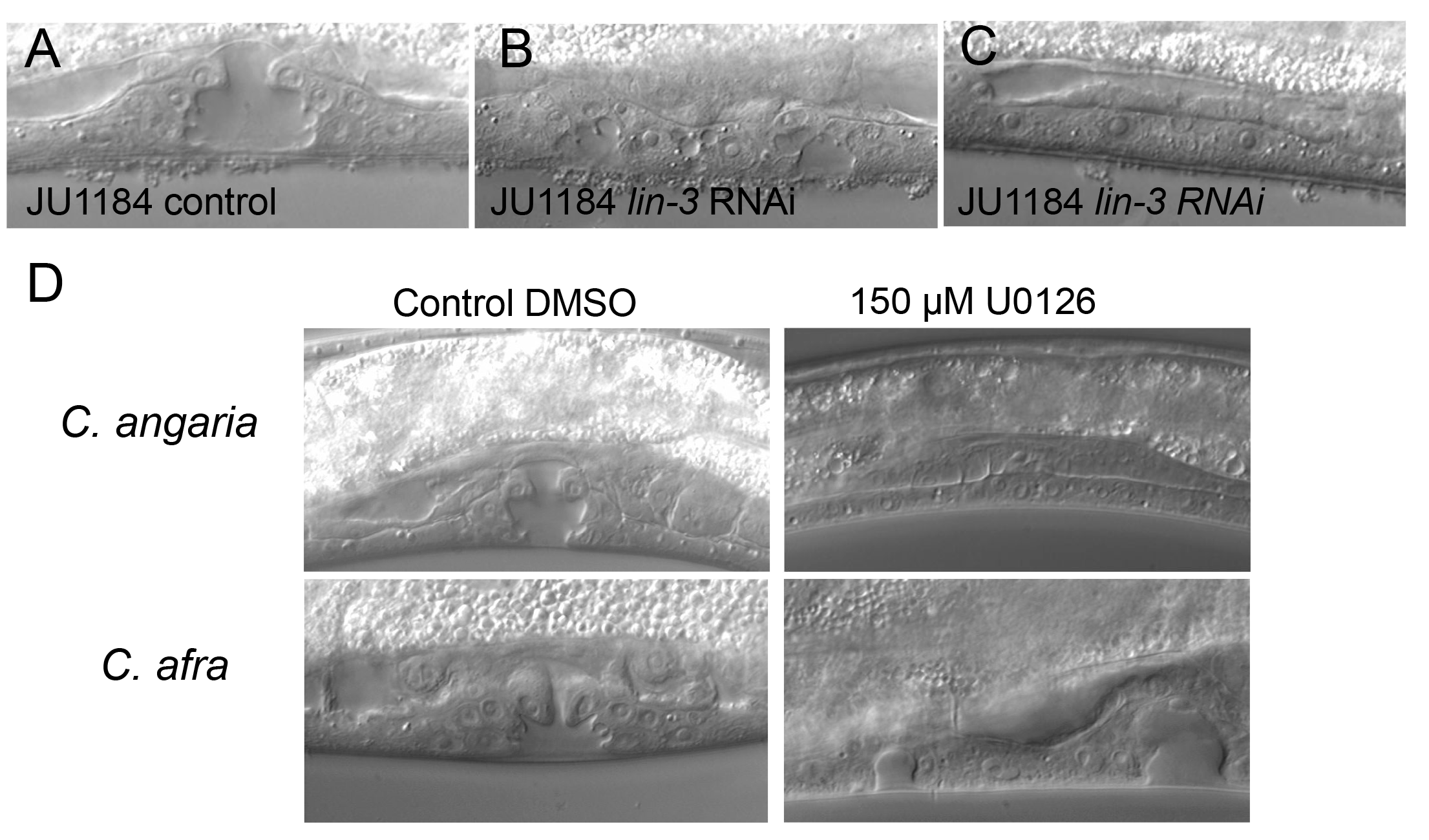

Supplement: S3 Fig — (A-D) Nomarski images of L4 stage animals upon lin-3 RNAi (A-C) or MEK inhibitor (U0126) treatment (D). (A-C) lin-3 RNAi by feeding in C. remanei strain JU1184 results in 2°-3°-2° (B) or 3°-3°-3° (C) vulval cell fates for P(5–7).p compared to the 2°-1°-2° of the wild-type (A). (D) Treatment with U0126 decreases vulval induction in C. angaria and C. afra. Note uninduced cells in both cases. The vulva in control L4 animals shows the typical “Christmas tree” morphology in all species. (TIF) [file pgen.1006278.s003.tif]

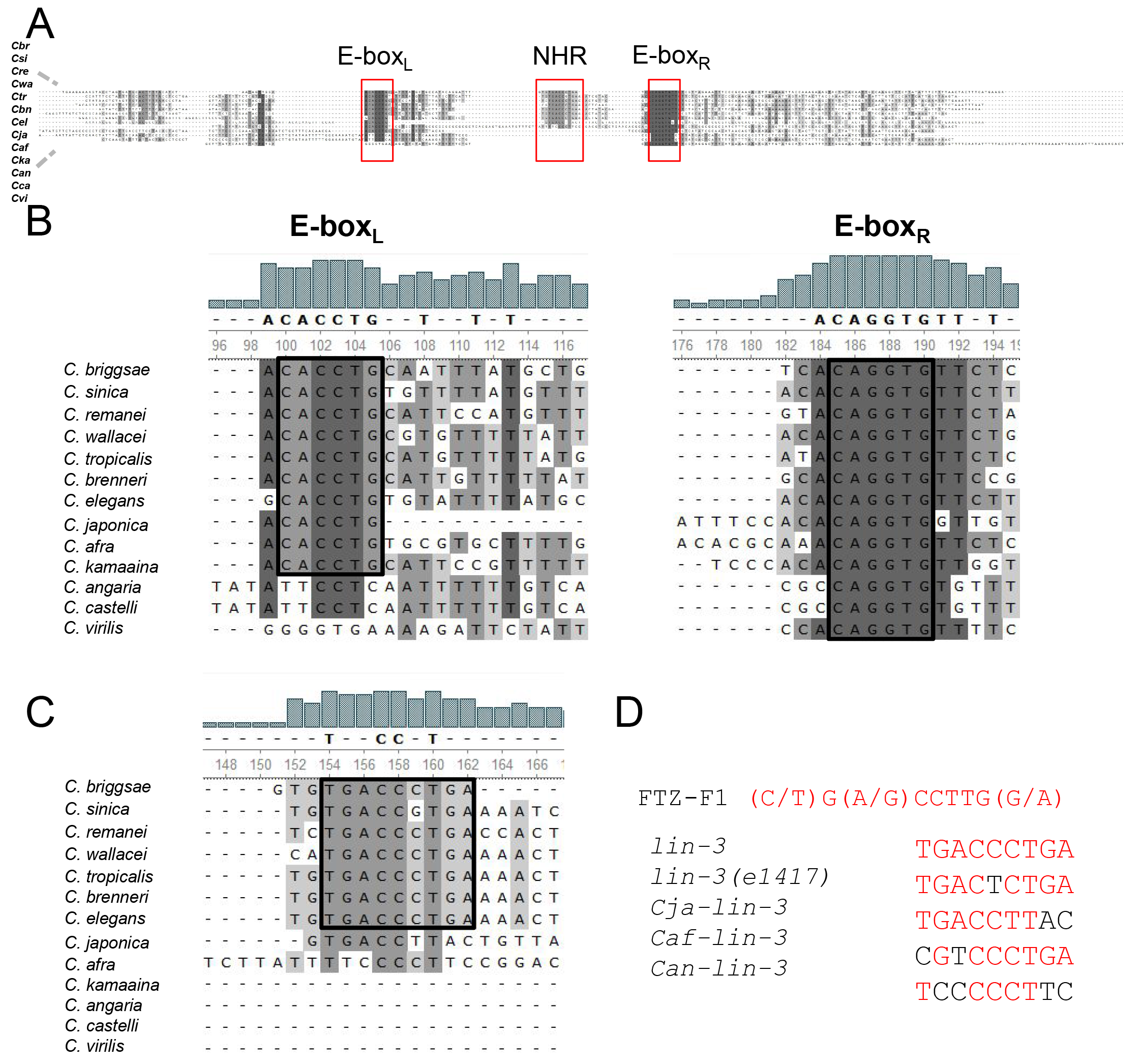

Supplement: S4 Fig — (A-C) Alignment of the 300 bp region upstream of the lin-3 ATG shows no other similarity in different species outside the E-box (B) and NHR (C) binding sites. Cbr = C. briggsae, Csi = C. sinica, Cre = C. remanei, Cwa = C. wallacei, Ctr = C. tropicalis, Cbn = C. brenneri, Cel = C. elegans, Cja = C. japonica, Caf = C. afra, Can = C. angaria. (D) Comparison of the Drosophila FushiTarazu/F1 (FTZ-F1) binding site, the NHR-binding site in wild-type C. elegans and lin-3(e1417) mutant. At least two nucleotide changes are required to align putative NHR binding sites from the Japonica group of the Caenorhabditis genus to the sequence in C. elegans and multiple changes are required for C. angaria. (TIF) [file pgen.1006278.s004.tif]

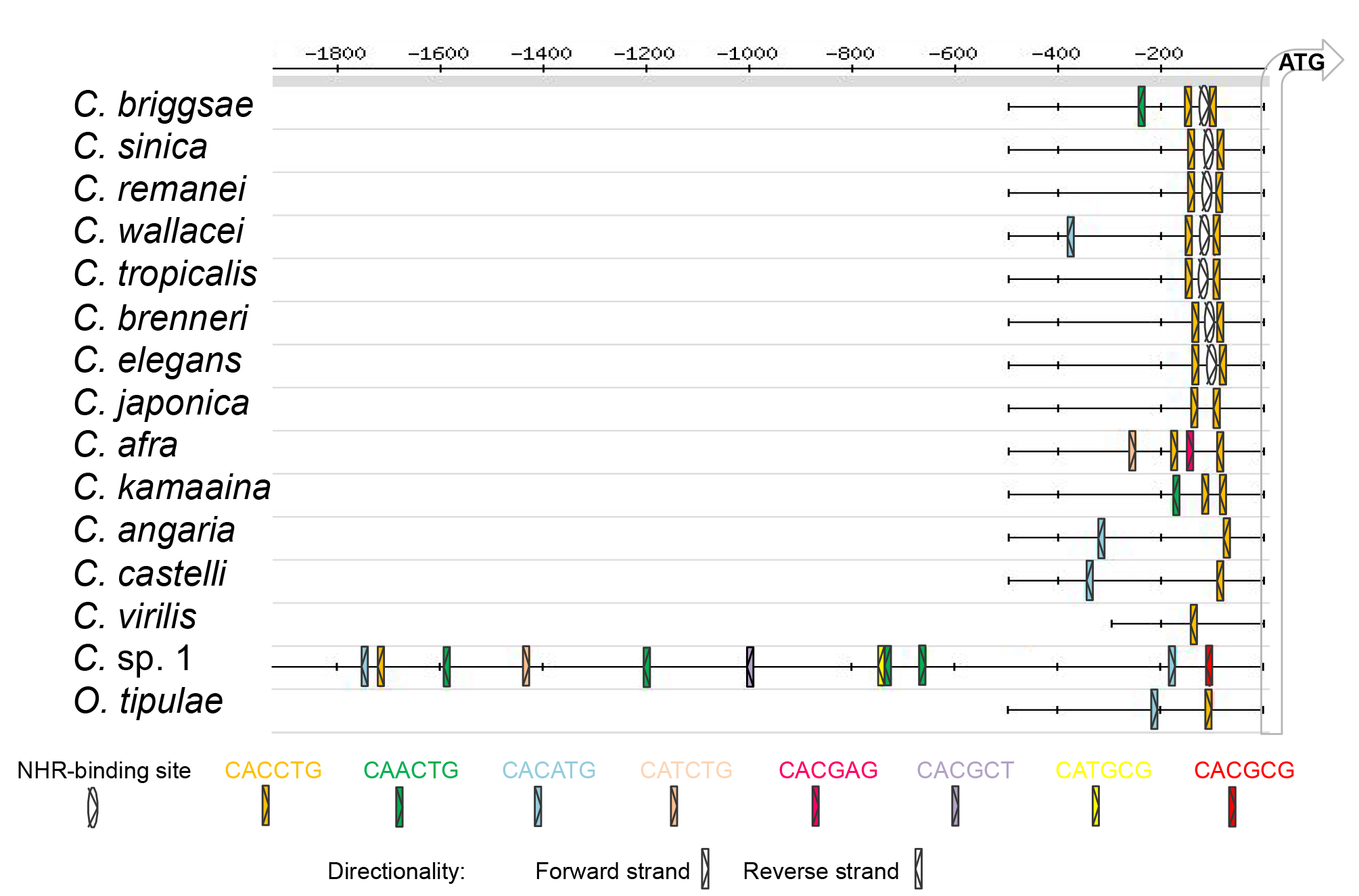

Supplement: S5 Fig — Location of transcription factor binding sites belonging to the bHLH protein family (as described in [51]) across DNA sequences upstream the TSS of the vulval form of lin-3 mRNA. The location of the NHR-binding site belonging to the lin-3 regulatory triplet is also depicted. Only the first 500 bp before the ATG are displayed for most of the species, except for C. virilis (only 300 bp) and C. sp. 1 (up to 1.9 Kb). (TIF) [file pgen.1006278.s005.tif]

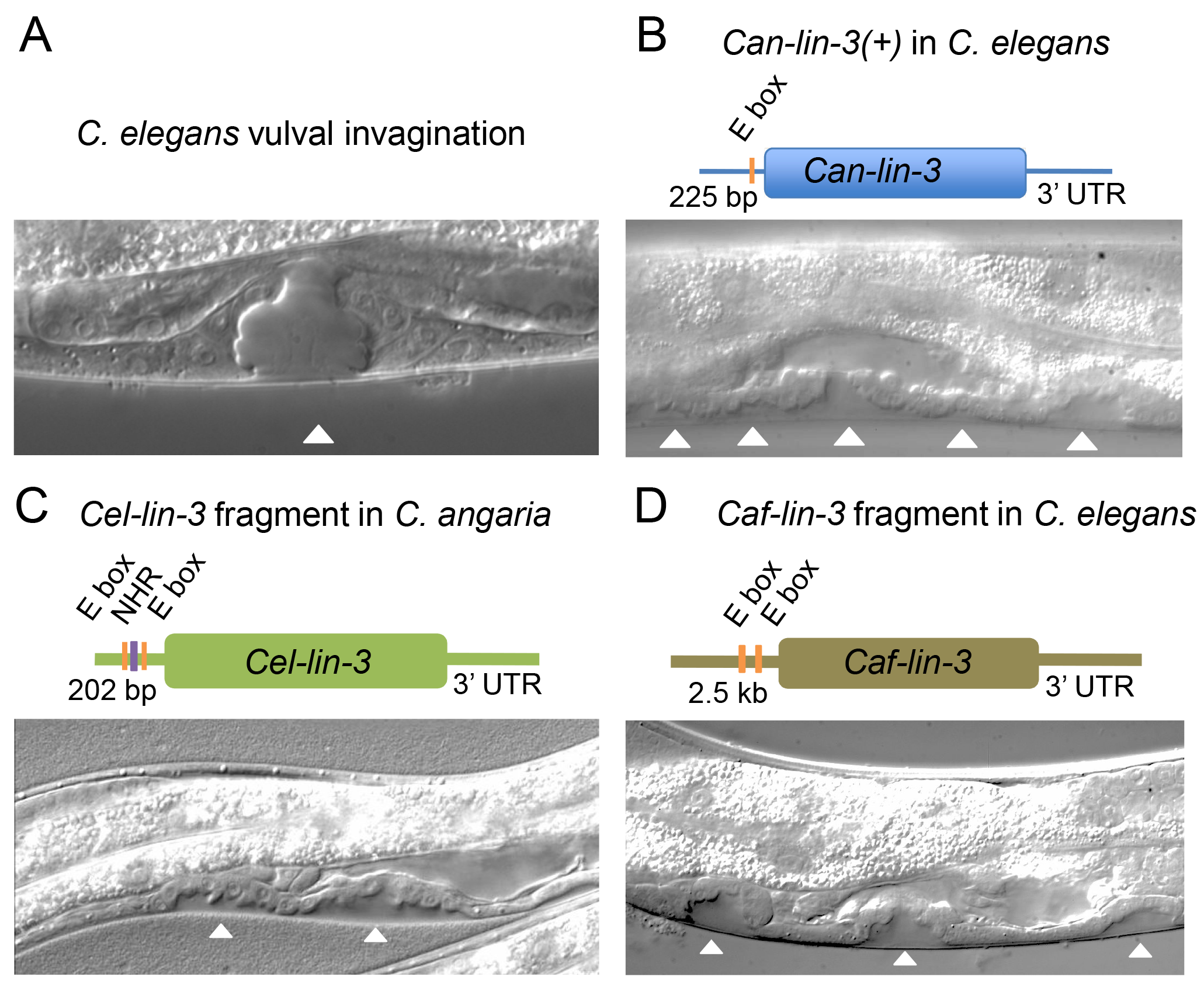

Supplement: S6 Fig — (A) Wild-type C. elegans vulval invagination in the L4 stage as seen by Nomarski optics. (B) Over-expression of Can-lin-3(+) in C. elegans via transgenesis with repeated extra-chromosomal arrays results in vulval hyper-induction, with several additional invaginations in the L4 stage (arrowheads). (C) Injection of a Cel-lin-3 fragment in C. angaria leads to vulval hyperinduction. (D) Over-expression of a Caf-lin-3 fragment in C. elegans with repeated extra-chromosomal arrays leads to vulval hyperinduction. (TIF) [file pgen.1006278.s006.tif]

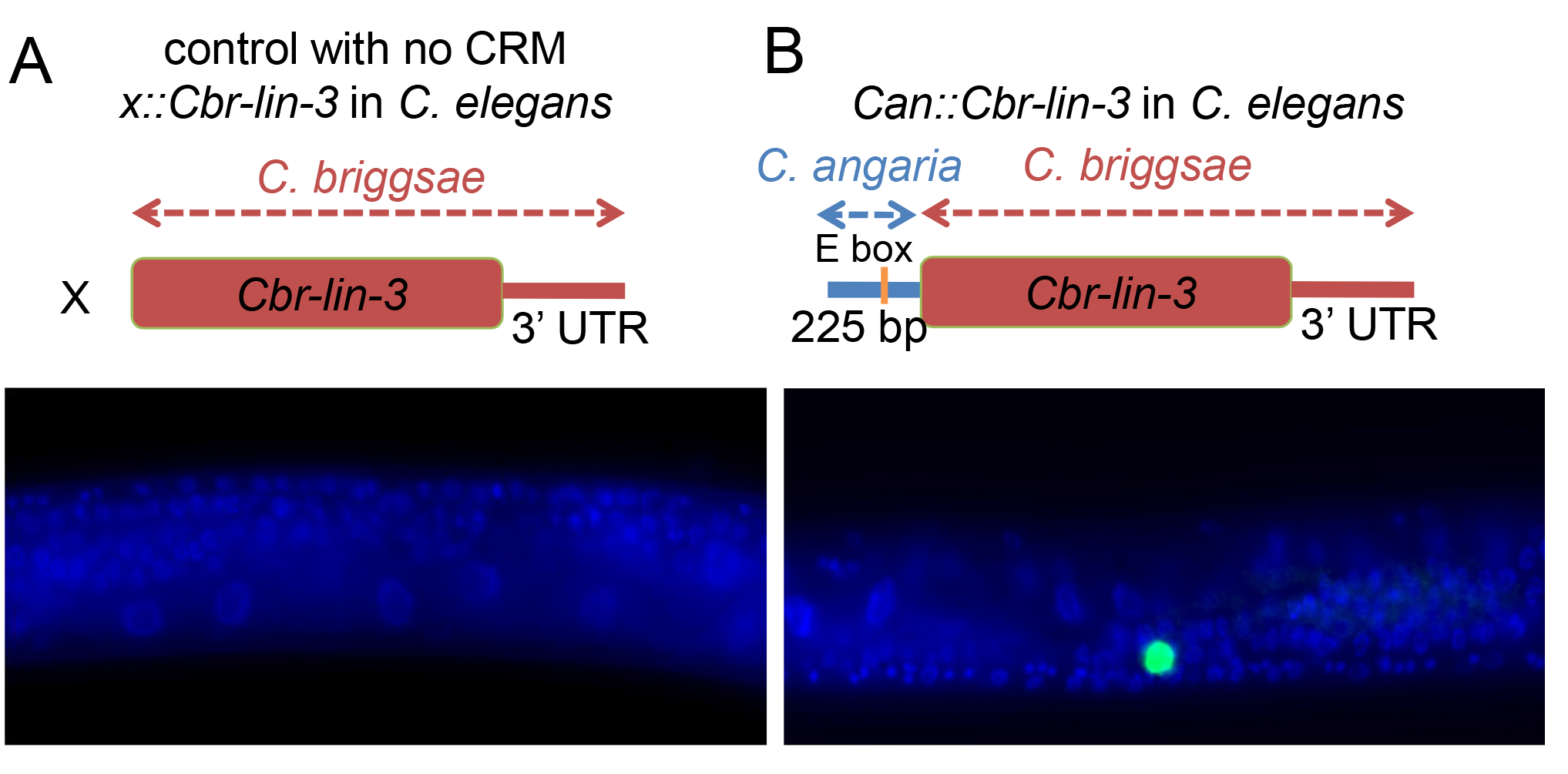

Supplement: S7 Fig — (A) A promoterless C. briggsae fragment introduced into C. elegans is not expressed in N2. (B) The same fragment under the Can-lin-3 CRM drives expression in the anchor cell of N2, as monitored using Cbr-lin-3 FISH. Green corresponds to lin-3 expression and blue is DAPI staining of nuclei. (TIF) [file pgen.1006278.s007.tif]

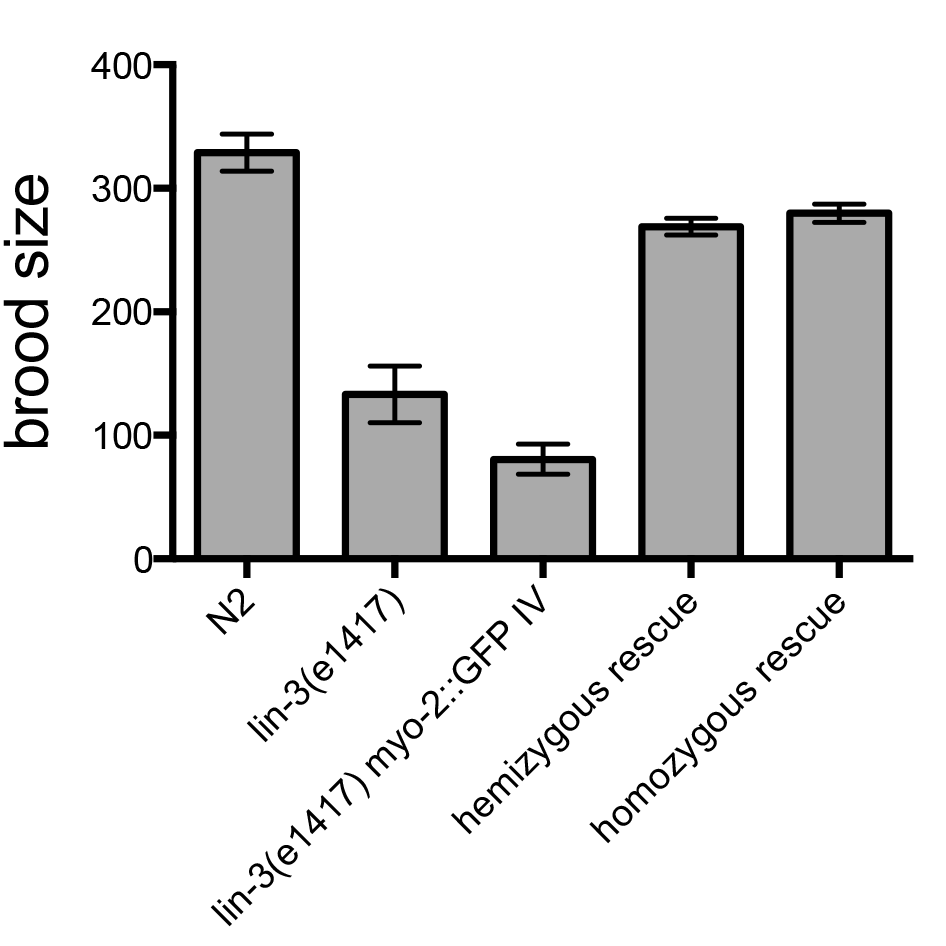

Supplement: S8 Fig — A single-copy insertion of Can-lin-3(+) rescues brood size defects of lin-3(e1417) mutants (n>15). Note that the presence of a myo-2::GFP transgene linked to lin-3(e1417) in the background enhances the lin-3(e1417) brood size defects and does not allow rescue to wild-type brood size. Vulval induction in this experiment is presented in Fig 5C. (TIF) [file pgen.1006278.s008.tif]
