# Supplementary material for: Evolution of New cis-Regulatory Motifs Required for Cell-Specific Gene Expression in Caenorhabditis
Source: PLoS Genet. 2016 Sep 2;12(9):e1006278. doi: 10.1371/journal.pgen.1006278 (PMC5010242; doi:10.1371/journal.pgen.1006278)
Supplement: S2 Fig — The sequences of the enhancers used in Fig 5A are in bold. The endogenous 3’ UTR used for the overexpression experiments in Figs 5B, 6A–6D, S6 and S7 is underlined. (DOC) [file pgen.1006278.s002.doc]

*LABELLING SCHEME:*

**Enhancer** - 3’UTR sequences for figure 5 and 6

FIRST ATG SECOND ATG EXONS

E-BOX NHR-BINDING MOTIF GTTTATG site

>C.BRIGGSAE

ATGCGGAATCTACTCCTATTCTGCATCATCCTCCTACTCATGCCTCAGTTTTCAGGTAAATAATAAAGTCAACGCATATTAAAATTCACCCCTAAGGATGTTCGCAATTTTCGAAATCATCCAATTTTCAAAATTTCAAAATGTTCGGAATTTTTCAACTTTCAAATTTTAAAGTAGAAATCCCATTTTCCCATCACAAAAAATCGAAAATTTATGAAAAATTTACGAAAAATATTTCGAAAACGCAAAAAAAGTTAAAGTAATTCCGCTCTAGACCCGCACGATAACTTTTCGCGTGCGCCTTTACCCCAAAAAAAATCAGAAGAGTTCATATCGGAATTTCCGGAATTTTCGGGATTCCTAATTCCGTTCCGCTGATGAGCCTAGAGAAGAGAGTTCACCAGATCTTTCTAAAACATATTGAACGGATCGGTTGATGATATTTGACACAACCACTAACACTACCACCAGATGTGTGCCTATGTGTTCTACATGTCGTCATACATGTGCATTAACCAGTGTCATATGCCCATTCAACTTACTTTTCCTCCCAAACGACTCTGAGAACTAGGGACTTTAGGATATCTCACACCAACAAACTCCCCCGATTGCAAATTGTCAACCTTTCGAACGGCCAGTGGGTGGTAGTCGTAAATATTCAAGTACCCGGTAGGAACACCGCCAAAATGTATGCACCACTCATAAACATTGTCACCTTTATACCGCATATATCGGTATTACTTTCACTTTGAACTTTTATCGATTCTACAACTTTACCACCTTGATCCATGTACATTCCGGTGGTTTCTTATGCAAGTTTGGACGAACCTTTACGGGGTAAGAAATCTACAGTAACCCAGGACGGGGAACAAAATTGAGAAAAGTTGAAATTTTGGAATACGCATTGATAGAATAGGACAGTTGTTAAGTTGGAATACAATAGAAAAAACTGTACGGTATT**TTGAAGAAAAGTACCTAGTGATGCACTTTTACATCTAATAGTTGGAACACCTGCAATTTATGCTGCCATACAGGATTTGTGTGACCCTGATCACAGGTGTTCTCGTTAGAAATTTCCCGCAAGACATTGAAAATTACCAAAGAAAGTACTGTACAATTGCCAAGGCTTCTTATATTTCTTTTAACTACTTTCATGAAGA**ATGTCTCGTTATTCGGTTCCAGAACGACTTTTGATTGCATTTGGTAGGTAGACTAGAGTATAATAAAACGGAAATTAAGAATCGAACCTTTGGTACATCCTTGAATTATCAAAAGTTTCAGAATCGTGTCTTCCGTCGTGGTTCCGCCAAGAACGTAGTGCTCCAGAAAAATTTCAATCTTCTGAAAATGCTGAATTCGCTTCCGAGAATAGTGGATCTCCTCCATCGGATTCCCCTAGGAATGCACTGGATACAAACGAGATTGGAGAAGCACCCTCTACAAATTCGAATGTGCCTGGTGCGACTACAGTATCTTCTGATGTAGTTGAAGGAGAAGAAGAGCAGTTGCAAGAAGTTGTCAAAAATTTAAGTGAGTTTTTTCGCGAAATTAGAAATTTTTTTAATAATCGAATGATTTCCAGCCGAAAAAGAAGCAGAATACGAAGATGAATACGAAGAAGAGAAAGAGGAAGAAGAAGCAGAAGAAGCTCTAAAATACAATGAAGATGTCACACGAGATGCAACTTCAACATTAAAACCTGCGGTACGAAAAGAGATAGAAAAGCTGAAAGAGGCAAAATGCAAGGATTACTGTCACCATAACGCCACGTGTCATGTAGAAGTGATATTCCGAGACGACCGAGTTTCAGCAGTGGTTCCTTCTTGCCAGTAAGCGAACTAGTTTAAAGCATCGTTTATTTTTTCAAAACTTCAGTTGTCCCCACGGTTGGGAAGGGACTCGGTGTGATCGTCACTACGTACAGGCGTTTTACGCCCCTATCAATGGCAGATATAATGTACGTTTGATCACGATGAGCAGCACGGCGCAACTCTTCGTTCAAGTAATTATCCTCTCTAATAATTAAAACGAATCGAAAACTAACACTCTGACTTACTTATTACTCACCCACTGAGATATATTTGCATGTAGCATGGTTCATTTGGTTTAGCCTTATTTTAGATTGGTTTGCGGTGCTGGTTGTTTTAACTCAAAACAGTGGAATTATTTTGTGAAATTTTCTAGGGACCAGTGAAAAAACCAAAAGTTTTTCTTGCTCGAAATCAAACGGTTACTACAACGCCGCCATCAAACAAAGATTCTGATATTCCATCTATTCTCAGTGGTATTTATGATAGAATTGTGGTAAGAATGGCAAAAAACTTAGTGACTTAAAATTTGATCCAACGGGACGACTATCTACCTTCCGGTAACTATTTTTCAGCAGTCTTCATCCTCGGCCATTCCCGCTTTCGCCTTTCTTATCGTCATGCTGGTCATGTTCATTGCTATTGTTATTTACGCCTACAGACGGTAATCCAGAATCCTCTGAATTTAATCTCTCAGTAAAAGTTTTCAGTATGTCCAAGCGAGCAGACGACATGACCTATACAATGAGTCATATGTGCCCACCAGAAGCATTTACCGTTTTGAAAACTCCCAATGGTCGTCACATTCCAGTTCACCAGATGACTTCGTGCCCTGCTTCTGCTTCTACGGCATTACCACCAACTCCTCATCCATCTTCAGCTTCTGGAGGATCGAGAATACCAATGAGACAACAGGCGATTCGAAACAATGCCGATCAAGCTAGAAACAACTTTTTCAGCATTCTCAGAAGTCAGGGAACTATTCCATCGAGGTATTTAATTTCTTTGATTCAGTTTTCCAAAAGGTTCAAATTTTCAGAAATCTTAACGACGACGATACTCCGAAGCACTACAAATCGGTGCCACGTGTCGAAGTTTCAGCCATAAACTATTCGGGACACATTGATTTCTCCACTATATCTTTTCAGTCGGTACGATTTTTCTTTTCTTTCAAATTCAATGAAACTTCTGTTTTCAGACAGATTCAGAAGTTTCGAAAGCATCAAAAACATGTCCACCACCTACACATTGTGTTATTGATATAGAACAAGATTCAGCGGATACAGTATGTTCCAATTAATTAAACTTTTAAATCAAATCACTTTCCAGAACTTTCGATCTCCCTCTCGGAGTTCCGGAGAACCAGGATCACCAACAACCTGTGAACCAATGATTCCTCATATGCATCTTTAAtttttaattgctcatttttccttctacttcttttctgtgctataattgtgatctacccatttgtttggtctctctcccccattttcttttcgctttctcttattccatatcgttcttgacaccataccgtttttttatagattgttctttatttgcaccctcatcaaatccgtccgttcaaactatattgttattcccttgctcatttcctgtgacaatgtgtatatttttgccagttcgtctcccatatcttctcacgggttccccgaccaaattcattttgtgcacgcaattcgattttcgatttctcccctgtatattactagtcgtttttctgctcaggttctg

>C.SINICA

ATGCGGAATCTACTCCTTTTCTGCATCCTTCTCCTACTCATGCCTCAGTATACCGGTAAGTATAGAAAAACAGAACTAATAACTCGAGAATACCGTGAATTTCTTCAAAAATTAGTTGAGTAGCCTCACACCTTCTAAAACATATTGAACGGATCGAATGATGATACTTGACACGACCACATGTGTTCTACATAGTCGTTTTTTTGCTGGTGCATTAACCAGTGTCATATGGAATGAAAATGTTCCCCACAACCACTACTAACTTCTCCTCAAACGACTCTGGGAACCAGAAGGGATATTTGGATTCCTTGATCATAACTAGGAACTCCCCCTCCGATAGAAAATTGTCAATTGGTGTCACCCGTCCACAGTAGGTGGTCGTAAATATTCAAGTACCCGATAGAACACCCGCCAAAATGTATGCACCACTCATAAACATTGTCACCTTCACACCACATATGTCGGTATTACTTTCACTTTGAACTCCTACACAACTTTACCATCATCCATCCGTTATGTACATTCCGGTAATATCATATTTGGATCGAGATGGACCATTAAATGGTACGTAAATGGAAATGAGGGAAAAGTAACTAAAAAGGATTTTTGAAACGTTTAGGAAGGTCTAGCTGTTATATATGGATATGCCGTAAAAACTGTGATAGACCGACACCCAGAATTTCAACTTTCGTACGAAAACAGCTTTCATAATTTTCGTTCCAAATTTTCAACATTCCACAAACAACTAATCGGCTAGAAGAAATTCTGAGTTTGTCGACTTGGGGATCAAAGGTTTTCTTAAGATTTATGTTAAGAAATAGCTGGTAGGGTGTCGCTCTATTACAGTTTTTACGGTGAATTTCAACTCCCCACTTCAAAAACAGTAGTATCCCTAGTTTCTTCCCATTTCCTATTTTGCACTTTTACCTCCTGACCATACTTTTCTCGTTCGGGACACCTGTGTTTTATGTTTTTATGCTTCATTTTGTGACCGTGAAAATCGTACACACAGGTGTTCTTCTCGAAATTTCCCGCAACACACTAAAGATTAGTATTACTTACGAAAATCTCATATATTTGTTCCCATTCATTAGTTAAGTAATGTCTCGGTACTCGATTCCAGAACGACTTCTAGTGGCGTTCGGTAGGTAAATTTTGCTCAAGTACCTAGTTTTTAACACTAGCTACTTCTGAGAAAGTTCAGCCCAAATGTCTTTCGACTGTTGCTCATGATTTCAGAATCTTGCCTTCCGTCGTGGTTCCGCCAAGAACGTAGTGCTCCCGAAAGGTTTCAATCTTCTGAAAATGTCGAAAATGCTCCTGAAACTAGTGATTCTCCTTCACCCGAATCGCCCAAAAATGCTCTAGAAACCAACGAAATCGGAGAAGCACCGACGGCCACTTCTGAGGAGACCTCTGATGTGACTACCGTAACACCCGAAATCAGAGAAGAAGAGGAAGAAAAACAATTGGAAGAAGCAGCTAAAGAATTAAGTGGGTTTCTTGAAATTTCTAGACAGAAAGATGTTTTGAATTTTCAGCCGAGAAAGAAGCAGAGTATGAGGAAGAATATGAAGACGAAAGAGAAGAGGAAGAAGCAGAAGAAGCTCTGAAATACAACGAAGAAGTCGTTCGAGATGCCACGTCAACATTGAAACCCTCTGTGCGCAAAGAGATAGAAAGACTGAAAGAGGCGAAGTGCAAAGATTACTGTCACCATAATGCAACGTGCCATGTGGAAGTGATATTCCGTGATGACCGAGTCTCGGCAGTGGTTCCATCGTGCCAGTAAGAATACTGAACTGGTATATTTGCTATATTCTAATTACAGCTGTCCTCACGGATGGGAAGGTACTCGTTGTGATCGTCACTATGTACAGGCGTTTTACGCCCCGATCAACGGCAGATATAATGTACGTTTGAGCCCGATGAGCAGCACGGCGCAACTCCTCGTTGAAGTAATTATACCCTCTCATCTAAAAATATATGTACTAACTATCCTTAACTAAACCCCCTTGCATGTAGCATGTTCATCTTAACTCAGTTCTCTGATTTGGTTTGGTTTGCGGTGGTGGTGATTCATTTTGAACCAAATCGAACTTTCAAATTTTCTAGGGTCCTGTGAAAAAACCGAAAACTTTCATTGTCCATTCTCACAATGAAACGGTTTCTACAACGCCTTCCAGTGAAGATTCTGATATTGCGTCGGTTTTCAGTGGGCTATATAATAGAATTGTGGTAAGAAGCGTAATCTAGGAGGACCTATTCCAACTCAGAAATATGAAAGGATAATAGACATGTCTACTTCCGCTTACCATTTTTCAGCAGTCATCAACATCTGCCGTTCCTGCATTCGCCTTTCTCATCGTCATGCTCATAATGTTCATCGCCATCGTCGTGTACGCCTACCGAAGGTTTTCAAGATTGAAGCTGGACATTCCTTCAACTTTCAATTTTCAGAATGTCTAAACGGTCAGATGACATGACCTATACAATGAGTCATATGTGCCCACCTGAAGCCTTCACCGTCTTGAAAACACCCAACGGTAGGCACATTCCTGTGCATCAAATGACGTCCTGTCCTGCATCTGCTTCTACCGTAATCCCTCAAACTCCACACCCATCGTCAGCTTCTGAATCGAGAATACCAATGAGGCAACAAGCGATTCGAAACAACGCCGACCAGGCCAGGAACAACTTCTTCAGCATTCTCAGATCTCAAGGAACTATTCCATCCAGGTATTTATTTTTGAAAAAAAAACTATGTCTGATTAAATTTCTTAATTCCAGAAGCCTAAACGACGACGATACGCCGAAGCACTACAAATCAGTGCCACGTGTCGAGGTGTCTGCCATCAATTATTCCGGTCACATCGATTTTTCCACCATCTCTTTCCAATCGGTATGAGATTCTAGATCAAATTTTAGGTTATTCACATTCACGACTTTTCCAGACAGATTCAGAAGTTTCCAAAGCATCCAAAACGTGTCCGCCTCCTGCACACTGTGTTATCGATATTGAACAAGACTCGGCGGATACAGTATGTTCTGAAAACTCGTTTAATTTCAAATTGATTTAGATTCAGATCTTCCGNNNNNNNTCTTCCGATCACCGTCTCGAAGTTCCGGAGAACCCGGATCACCGACAACCTGTGAACCAATGATTCCTCATATG

>C.REMANEI
ATGAGAAATCTTCTACTGTTTTGCATTCTACTCCTGTTCATGCCTCATTTCACAGGTAATTTTTTGAAGATTAAAACGACTTCAGAGTCACTAAAAACTAATCCAGATACATCATTTCGAACACCTTCTCAAACATATCGAATGGGTTGAATAACGATATTTTACACGATGACCACATGTGTTTTCTACATTTCTTTTTTGTGTTAACCACTGTCATATGGAACAAAAAACATTCTTGCCACCACTAACTTCTCCTCAAACGACTCTGAGAAAAGCGAGGGATTGAATGTCTCAGCCAGAATCCACATTCCAAATTTCTCACTATAACTTATTAGTACCCGATCGGAAATTGTCGAATTGTTAAAAGTGACCCTACACAGTAGGTGGTCGTAAATATTCAAGTACCCGAATATCGCCAAAATACATGCACCACTCATAAACACCCTCTTCTTCACACCACATATCGGTATTACTTTCACTTTGAACCCTTCTACTACAATTTTACAACCGATATTCGACTATGTATATACCGGTTATATCTTATTTAACTTCAAAAGATTCGATACAGGGTTTGTGATAAACAGTGACTGGAAAATACAGATACCGACATTAGTCAAAAATTAGAAATTTTTACTTACAGACAACCAGTCGGCACGATTTGGGTTCAAGGTAGTTGACAAATAGATCTAAATTTATCTCACCTATAACGTAGAACACTTCGAAATATCGAAAATTTCGATTCTATAACTCTAGTTGTACTTTTAACCCTAGTTATCTCGTCCTACCCGAAACACCTGCATTCCATGTTTTTATTCTTTTTTCTGACCCTGACCACTGTTCGTACAGGTGTTCTACACTTTTCTCCCGCAAGAAAGTGCCGAAAAACTTTTCGAAGCTTTTCGTAAGATTCAAATTATTCATTCTACAAATATTGAAATTATGAGTATTCACTCTATCCCAGAAAGGCTTCTCATTGCATTCGGTTCGTTTGATTAGTATGACTGCTGGTTGAAAATTTAGATAATCTAACTGTATAGCTTTTCATCACTCTAAAAAAGTTTCAGAATCATGCCTCCCTTCGTGGTTTCGACAAGAACGTAGTGCTCCCGAAAAGTTTCAGTCCTCGGAAAATGCTGAAACAAGCGGCTCTCCTCCAACTGACAGCTCACGGAATGATTTAGAAACGAATGAAATAGGAGATGCTCCATCAACTACTTCTGACGTTGAAACAACCACTGAAGTAACTTCTGTGACATCCAATCAGAAAGAATTAGAAGAAGAAAAGAAATTACAAGAAGTTGTCAAAGAATTGAGTTAGTTTCGACTGTTTGAACTCCTTTATAACTTCAAATGATTACAGCGGATAAAGAAGCAGAGTATGAGGATGAATATGAAGAAGAACAAGAGGAAGAAGACGCAGAAGAAGCATTAAAATACAACGAAGAAGCTACTCGAGATGCCACATCAACACTCAAACCATCAGTTCGAAAAGAGATTGAAAAGTTGAAAGAAGCAAAATGCAAAGATTACTGTCATCACAACGCGACGTGCCACGTGGAAGTGATATTCCGTGATGATCGAATTTCAGCAGTGGTTCCTTCTTGCCAGTAAGAGAGTCACTCTTTATAAAACTGCTATGTTACATTTTCAGTTGTCCACACGGTTGGGAAGGTACTCGCTGTGATCGTCACTACGTACAGGCGTTTTACGCCCCGATCAATGGCAGATATAATGTACGTTTGAGCACGATGAGCAGCACGGCGCAACTCTTCGTTCAAGTAATTACACTCTCTAATCTTCAAATATCAAATAACCAAACTAGCATGACATTCCCCACAAAATTCCAACTAATCAGTTCCCTATTAAACGCATGTTTCATTTTGTTTTTTCGGTTTGTTTGCGGTGTTGGTGCTTTAATCGGAATCATACAATTTTTGTGAAATTTTTCTAGGGACCAGTGAAAAAACCAAAAGTTTTCATTGTTCATCCTTCCAATCACACTGTTACTTCCACGCCTTCCAGGGATGATTCTGATATTTCGTCTGTTTTCAGTGGGCTTTATGATAGAATTCTGGTAAGAAGCAGAAGATAGTGTCTATAAAAATTAGTTTTAAAAAATGAGTATATCTTTATGATCTAGTAGATAACTGGAAATTCGAGCTACTGATAGATATCACAAACGTTCGCTGACCCACTTTCCAGCAGTCTTCAACATCAGCTATTCCTGCATTCGCATTTCTTATCGTCATGCTCATTATGTTCATCGCAATTGTTATCTACGCTTACAGAAGGTATTTTCATCTTTTTATCGATGGTTCCACCATTCAATATATGTATTACAGAATGTCAAAGCGTACAGATGATATGACGTATACAATGAGTCATATGTGTCCACCTGACGCTTTCAATGTCTTGAAAACTCCAAATGGGCAGCATATTCCTGTGCACCAAATGACATCTTGCCCTCATTCTGCCGGACAATCTACAGTAATTCCTTCAACTCCACATCCATCGTCAGCTCCTGGATCAAGAGTACCAATGAGACAACAAGCGGTGCGGAATGTCGATCAAGCCAGAAACAACTTTTTCAGTATTCTTCGAAGTCAGGGTACTATTCCATCCAGGTATCAAATTAACAAGAAGTACGAAGTAAGTGAATGTTTTTAGGAGCATAAATGACGACGATACGCCGAAGCACTATAAGTCAGTGCCGCGTGTCGAAGTTTCAGCAATCAATTATTCCGGACATATTGACTTTTCCACCATATCTTTTCAATCGGTAAGAACCCCCTTTTTGATTGTTCTGATTTGTCTCAATTTCAACTTTTCAGACAGATTCAGAAGTTTCAAAAGCATCTAAAACGTGCCCACCGCCTACACACTGTGTGATTAATATTGAACAAGAATCCGTGGAAACGGTATGTGTATCATTGCAGGATCATTTTGATATAATAATATCTCAACTTTCAGAATTTCCGATCACCATCCCGAAGTTCGGGAGAACCAGGATCTCCCACAACTTGCGAACCGATGATACCTCATATGCATCTATAA

>C.WALLACEI

ATGAAAAATCTATTGCTTTTATACATTCTTCTCTTATTCATGCCCCAATTAACAGGTAATTCTTTTTTTGAAATCTTCTCAAAATCAAAAAAAAAAAGATCCGAATAATTTGTCATTTCAAATGAACACCTTCTCTTGTTCAACATGTTGAATAGATCGAATGAAGTTATTTGACACTGCCACACATGTGGTTTATACACTTTTTTGTGCACTAACTGATGTCATATGGGAAAAAAAACGAACACTTGTAAAACCACTAACTTCTACACCAAAATGACTATGAGAATATGAAGGGATTTTCCAAGATTCTAGAAAAACGTTTCCCTATCTCGTTGGTGATTTCCGTTCACCCGATTACAAATTGGTGAATTCATAAATGGTGACCCGAACACAGTAGGTGATCGTAAATATTCAAGTACCCGAATACCGACACATGTAAACCCTCTCATAAACACCCACTTCTAGAGTAATATCCCATATATTGATACCACTTTCACTTTGAACCTTTCAAATTACTTTTGTATATATCTACTATTATGTATATTCCGGTTATCACATATCTGGATTCAGAAATACAGGGTATGTAATGGATTATTTGAAAAACAAAATTAAGAAGTACAGTATCCCGTTGTTGCACTTTCATATCCATTCCATGTTTCCTCAAGACACCTGCGTGTTTTTATTCTTTTTTCATGACCCTGAAAACTCAAGACACAGGTGTTCTGTACATTTTTCCCGCAACGAAAAAAGAAAAAAGTGTCCATACTTTTGGTGGGATTTCACTAATTCCACTATACGATATTCTAAATTTAATATGTTTGGATATTCAGTTCCAGAAAGACTTTTGATTGCATTTGGTATGTCCATCTATGAAATAGATGACGGGAAAAGATGAAATGTTCAACTGACATCTGAATAATTTCCAAAAAGTTTCAGAATCCTGTCTCCCTTCATGGTTTCGTCAAGAACGTAGTGCTCCCGAAAGGTTCGAAGCATCTGAAAATTCTGAATTATCCGCTGAGAATAGTGGTTCAACTCCATCTGAATCTCCTCGAAATGCTCTAGAAACAAATGAAATTGGTGAGGCTCCTCCATCGACAAATTCTGAAGTGAACAGTGATTTGGAAACAACATCAGAAGCAAAGAAAGATGAAGAAAAGAAATTAGAAGAAGAAGCTGTTAAAGAACTTGGTGAGTTAAAATAGAAGAATTCAAATAGAACGTTTGGATGATTTCAGCTGAAAAAGAAGCAGAATATGAAGAAGAGTATGAAGAAGAAAAAGAAGAAGAAGAAGCAGAAGAAGCATTGAAATATAATGAAGATGCGACACGAGAAACATCAGCAACTTTAAAACCATCGGTACGAAAAGAAATTGAAAAGTTGAAAGAAGCCAAATGCAAAGGTAAGCATCTCAATCTATTCATTTAAGACTGAGAGTGGTTTCTAGATTACTGTCATCACAATGCGACGTGCCACGTGGAAGTGATTTTCAGTGACGATCGAATTTCAGCAGTTGTTCCTTCTTGCCAGTAAGAATTCTAATTCTTTTTTTTTTCATTTAGAAATGTTTTTAGCTGCCCGCATGGTTGGGAAGGTACTCGTTGTGATCGTCACTATGTACAGGCATTTTATGCTCCGATCAGCAACAAATATAATGTACGTTTGAGCACGATGAGCAGCACGGCGCAACTGTTCGTTCAAGTAATTATCCTCTCTCCAATCTAAAAATTATTCATATCTTACTAATAACAAACGTAGATTTCTGTGTGTGTGTTTGTCTGTGGTTAGCGGACTCCAGAGCCTTTCCCCTTTATCCAAATTTTTTGAAACAAGGACAGGTCGATTCAGAATTTGACCGTGATGATGCCCGTCTTTTCCGTTTTCGATTCTGATAAGCGAGAAGCTCTGTATACTTATAGCACTTCCCAGGAGCAAAGTTGGGATCTCCCAAACAGGTGGTCTCTCAATTACCTCCTGGGAAAGTAGATCAAATGTGGGAGAAGGCGCGAAGCGTCGTCAGCAAGCTTGTAACTAACATTAACCAATATTAATCTACTAATTTTAACAAATTGCATGTAGCATGTCTCTCATTTGGTTCTTTTCTTTAGTTTTGTTTTGCGGTGATGGTATTTTCATCAAAATCATTTCATTTTCTTTGAAATTTTCTAGGGAACTGTGAGAAAACCAAAAATTTTCATCGTTCATTCTCCAAATCATACGGAAGCATCGACGCCTTCAAATGATGATTCAGACATTTCATCTATTTTCAGTGGGCTTTATGATAGAGTTTTGGTGAGAAAATATTCATTTCTTAGCTCTAAGTTTAGGTTTCTAGGTTTAGTAAATTCAATGTTCATTCAAATATCAAATTCTTATTTTCTTAAATTGGCCAAATTTCTTTTTCAGCAATCTTCATCTTCTGCAATTCCTGCATTCGCATTTCTCATAACTATGCTCATTATGTTTATTGCCATCGTTGTATATGCTTACAGAAGGTAATTAATTGTTGTGAAATCCTCTTTCAACAATTAATTTCAGAATGTCAAAACGAGCAGATGATATGACATACACAATGAGTCATATGTGTCCACCAGAAGCTTTTACAGTTCTTAAAACACCTAATATTCGACATATTCCTGTTCATCAATTACATTCTTCATGTCCTCAATCTGGTGGTCCTTCTACAGTAATGTCTTCAACTCCTCATCCTTCATCAGCTAATTGTCCAAGAATACCGATAAGACAACAAGCTGTTCGAAATAATGATCAAGCAAGGAACAACTTTTTCAGTATTTTAAGGAGTCAAGGAACTATTCCATCCAGGTAGTAAATATGAAGATTCTGTTCTTTTTTAAAGAATATATTTCAGAAGCATTAACGACGATGACACGCCGAAGCACTACAAATCGGTGCCACGTGTCGAAGTTTCTGCTATCAATTATTCTGGTCACATCGATTTCTCTGCGATTTCATTCCAATCAGTACGAAAAAACTAATCAAGAGTAAAATTATCTTCTTTTTCTTGCAGACCGATTCAGAAGTATCGAAAGCTTCAAAAACGTGTCCTCCTCCAACACATACAGTTATTGACATCGAACAAGATATTGTCGATACGGTACTCAGATTCATCCAAGTATTCCTCGAAATATCTCTTTTCAGAATTTTCGATCACCATCTCGAAGTTCTGGAGAACCTGGATCACCAACAACTTGTGAACCAATGATTCCTCATATGAAATTATAA

>C.TROPICALIS

ATGAAAAATCTATTGATCTTCTGCATCCTACTTTTATTGATGCCCCAATTAACAGGTAATCATTTAAATAGAAAAATAGTCATTCAAACGAACAGCTTCTTTTCACAATATGTGGAACGGATCGAATGAAAGTTATTTGACACTGACACTACCACATGTGTTTTATGTACGTTTTTGAGCACTAACTGATGTCATATGAGAAGAAAAACACTTGTACAACCACTAAGTTCTCCACGAACGACTATGAGAGTAGAGAATAGGGAAAGCCTCGGTTCAAAATACCTAGAATACAGACTTCTCTTTGTGGTTGGCGTTAATCCGGTTGCAAATTGGTGAATACATGAGTTGGTGACCCTACGCGCACAGTAGGTGATTGTAAATATTCAAGTACCCACCCGAATACCGCCAAATGCACCTCTCGTAAACACCCACTTGTCAGCGTTATATCCCATATATTGATACCACTTTCACTTTGAACCTTTGAACTATTTGTGTAAAACTTTACTACCTCTATGTATATTCCGGTAATTACATATCTGGATTCAGGACTACAGGGTTTGTTTGAGTGAAAGCTAGAATTTCAGTCCATTTCACATTTTGGGAACTAAAAGTACAGTATCTTTTTCTCTTTCAACTCATAAATAGTCCATGTCCACTCAAGACACCTGCATGTTTTTATGCTTCTTTTTGTGACCCTGAAAACTATACGAATACAGGTGTTCTCTACTATTTCTTTCGAAAGTATTTAAAAAAAAGTTTCACGAATTTTCCGTAATTTCGGCAAATTCGTTGTTCAGTTTTCTGAATTAAGAATGCTTAAATATTCAGTTCCCGAGAGACTTTTAATTGTATTCGGTAGGTTTGATTCTTTGAATTCAGAAGATTACTAAAAACCAAATCAACATCTAGATTTGAAGTTTCTGTAATTTTGGAAAAGTTTCAGAATCCTGCCTCCCTTCTTGGTTTCGTCAAGAACGTAGCGCACCCGAAAAATTTCAGTCTTCTGAAACTCCTGAACTATCTGCAGAAAACAGTGGCTCAAATCCATCTGAATCTCCTCGAAACTCTCTTGAAACAAATGAAATCGGCGAAGCGCCGTCGAAAACTTTTGATGTAGTCAATAATTTGGAATCGACATCAGAATCAAATGATAAAGATAGGCAAGATGAAGAGAAGAGACTACAGAAAACCGCTGAAGAACTAAGTAAGTTTCAATTGTTGAAATGTTGATAATTTTGAATATTCAGCCGAAAAAGAAGCAGAATATGAAGAAGAGTATGAAGAAGAAAAAGAGGAAGAAGAAGCAGAAGAAGCACTAAAATACAATGAAGACGCAACACGAGAAACGACTGCAACACTGAAACCATCAGTACGGAAAGAGATTGAAAAATTAAAAGAAACAAAATGCAAAGGTAAGTTTCTATCAAAAAGTTGAGATCTTTGTTCTTAATTTCCAGATTACTGTCATCACAATGCGACATGCCACGTGGAAGTGATATTTCGTGATGATCGGATATCAACAGTAGTTCCTTCTTGCCAGTAAGGCGACACCGTAGTTCTCTTCTAAAAATAAATGTTTTTAGCTGTCCGCAAGGTTGGGAAGGTATTCGTTGTGATCGTCATTATGTACAGGCATTTTATGCTCCGATCAGCAACAAATATAATGTACGTTTGAGCACGATGAGCAGCACGGCGCAACTGTTCGTTCAAGTAATTATACTCTCTCCAATCTAAAAAAACATTCATACTAACCCTTCCCTTACCAAAGATTGAACTACTAATAACTAGTAATTGCATGTAGCATGTTCCTAATTTCGGTTTTTTTTGTGTGCGGTGATGGTGTTTTCATGAAAACATTTTATTTTCTAAGAAATTTTCTAGGGAACTGTGAAAAAACCAAAAATTTTCATCGTTCATCCTCCAAATCATACGGAAACTTCGACGCCTTCAAGTAAAGATTCTGAAAGGTCTTCCATTTTCAGTGGGCTTTATGATAAAGTTTTGGTGAGAATCATTCATTTTAGAGACTTTAAAAAAACCAGATCTAGTATAAAAAGTAAATATTGAATTTCAATTTTCTCAATTTTACTCTAACCTCTTTTCAGCAATCTTCAACGTCAGCAATTCCTGCATTCGTATTTCTCATCACAATGCTCATAATGCTCATTGCAATAGTTATCTATGCCTACAGAAGGTATTCGCTGTATTTATTCAGAACTTCTCCCACAAAACCAGTTTCAGAATGTCGAAACGTGCAGATGATATGACATATACAATGAGCCACATGTGTCCACCAGAAGCATTTACAGTTCTTAAAACACCGAATGGGCGACATATTCCAGTTCATCAATTAACTTCTTCGTGTCATCAATCGGGAGGACCTTCTTCAGTAATCTCATCAACGCCTCATCCATCGTCAGCTCATGGATCAAGAATACCGATGAGACAACAAGCAATTCGAAATAATGATCAAGCAAGGAATAACTTTTTCAGTATTTTGAGGAATCAAGGAACTATTCCGTCCAGGTATAGGATTCGAAATTTCAAAAACACCTTTGTATCACTTGCAGAAGCATAAACGATGAGGACACGCCCAAGCACTACAAATCGGTGCCACGTGTAGAAGTTTCTGCTATAAATTATTCTGGTCATATCGATTTCTCTGCCATTTCATTCCAATCGGTATCACACTTCTATTGAGTTTAGAAAAAGATTTCTTCATTTCAGACCGATTCGGAAGTTTCAAAAGCATCAAAAACCTGTCCGCCTCCAACCCATACAGTGATTGATATTGAACAAGATACTGTAGATACAGTAAGTCGATTCATCTAAATTTAATCTCATTTCTCGATTTTCCAGAATTTCCGTTCACCTTCTCGAAGTTCTGGAGAGACCGGAACACCAACAACTTGTGAACCAATGATTCCTCATATGAAATTATAA

>C.BRENNERI

ATGAAATTTATATCCCTCTATTGCATCCTACTCCTGTTTATGCCTCAGTTCACAGGTTAGGTCTATTCAGAAATCATTCTGAACTAAAACTGAAATACTACTTCTTTATCTCCAGTGGGCCTCCCATTTGAGTTAACTTCATTCGAACACCTCTCAAAACATATTGAATGGATCGAATGAAGTTATTTGACACTGACACAACCACATGTGTTTTATACACGTTTTTGTGCACTAACCGATGTCATATGGAAAATGAACACTTCCAATACCACTAACTTTCCCCACAAACGACTATGAGAAAGTGGGGATTCGAGCTCCGAACAAAGAGTTTTCTGAAAGCATTCCTTCTTTTCATTTCCGGTTATCTCACTGCCAGATTGGCGAATTCATGAAAAGGTGACCCTACACGGCGCACAGTAGGTGATCGTAAATATTCAAGTACCCGAACACCGCCAAAATGCATGCACCTCTCATAAACACCCACTTCTCAACAATACCACCATTGATACTACTTTCACTTTGAACCTGTCCAATTACTTCAACGGATAACAACTATTTTACAACCAATATGTATATCCCAGTGATCACCTACCTAGATTGGGATAAGCCTTTACAGGGTTTGTATTAAAATCTGAAAATCTACAGTAGCCTCCTAAAAAATAATAAGTACAAGTTTGTCTGCGCAACGGAAGGATGTACTCTTCCTAAGTTTTTCTCATGAATACTCGGGACACCTGCATTGTTTTTATGCTTTTTGTGACCCTGAAAACTATACGCACAGGTGTTCCGTGCATTTTTACCGCTTCTAAAAGTATACGAATTTCTCCGGTTTCACTAATTCGGTTCCACAAACTTTTAAAATCTGAGATGTTCGGCTTATCAGTTCCCGAGCGTCTCTTGATTGCATTTGGTACGTTGAATAATTAGAGACAGGAATAGGAATTAGGATTTACTTGAATTTCATACTCCCACAACTGTATAAAAGTTTCAGAATCCTGCCTCCCCTCCTGGTTTCGACGAGAACGCAGTGCTCCCGAAAAGTTGCAGTCTTCTGAAAATCCCGGACTCACTGTAGAAAATAGTAGTTCAGTACCACCCGAATCACCTCGAAATGAATTAGAAACAAATGAAATTGGTGAGGCTCCGTCAACGACTTTTGAAGTTAAAAACACACTGGAAACAACATCTCAGTCCTCTTCCACCAAAGAAGATGTGGAGAAATTAGCAGAAGCGGTCAAGGAATTAAGTAAGCATTTCTTAATTCTATAGTTTTTGAAATGTTTCGTTTTCAGCGGAGAAAGAAGCAGAATATGAAGAAGAGTACGAAGAGGAGAAGGAGGAAGAGGATGCAGAAGAAGCGCTGAAATATAATGAAGATGCTACACGAGATGCCACGGCGACACTGAAACCATCGGTACGAAAAGAAATAGAAAAGCTGAAAGAGGCAAAGTGTAAAGGTCAGTATCGTTAAAAACGAGATCACTCTTGACCAATCAATCTCCAGACTATTGTCATCACAATGCAACGTGCCACGTGGAAGTAATCTTCCGTGATAATCGAATATCAGCGGTAGTTCCCTCTTGCCAGTAAGTTTTCAAATTAAATAAGAAACTAAAATGTGTTTCAGCTGTCCGCATGGTTGGGAAGGTACTCGTTGTGATCGTCATTATGTACAGGCGTTTTATGCTCCGATTAACAGCAAATATAATGTACGTTTGAGCACGATGAGCAGCACGGCACAACTGTTCGTTCAAGTAATAATTATACTCTCCAATCTAAAACATTCGAATCTAACCTTCGCTCATAACCTATTTTCCGTACTACCTCTAATAGAAGAACACAGAGTGTTTTCATAACAGTAGACCATTTTCGGTTACCTCACATCATAACTTCAAACTTACTGCATGTAGCATGGGTCGTTTTATTCATTCTGTTTCTTTTCTGAGTTTGATTGCGGTGATGGTGCTTTTATCGAACATATTTCATTTTCGTAAAATTTTCTAGGGAACTGTGAAAAGACCAAAAATATTCATAGTTCATTCTCCAAATCATACTTCCACCTCGACGCCTGCCAGTGAAGATTCTGATATTTCTTCCATTTTCAGTGGGCTCTATGATAGAATTGTGGTGAGAAAATTACTATTTTTAGAGATTCTCAGTTTCTTTTTGGAACGTTTAGGAAAAATAAATCAATCAATTATCAGAATGTTTTGGTACTCACTTTCAGCAATCTTCAACATCTGCCATTCCTGCATTCGCATTTCTTATCATCATGCTCATTATGTTCATCGCCATCGTTGTCTACGCTTACAGAAGGTTCAATCATCCCACTTTATTAGTCAAGGAATTCATTTTCATTCAGAATGTCCAAACGTGCAGATGACGTGACGTACACAATGAGTCATATGTGTCCACCAGAAGCCTTCACTGTCCTCAAAACACCAAATGGACGACATATTCCTGTCCATCAATTGACTTCTTCTTGTCCTCAATCTGCCGGACCTTCCTCAGCAATTTCATCAACTCCACATCCATCTTCAGCTCACGGATCAAGAATACCAATGAGACAACAAGCTGTTCGAAATGTTGATCAAGCCAGAAACAACTTTTTCAGCATTCTCAGGAGTCAGGGGACTATTCCATCAAGGTAACACTCGAAAGCATGAGATCCCACAAGTATTGGGAATTCTTCCAGAAACATCAATGACGACGATACTCCGAAACACTATAAGTCAGTGCCGCGTGTCGAAGTTTCAGCAATCAACTACTCCGGGCACATTGATTTCTCCACAATATCATACCAATCGGTTATTACAAATTATAAATTTTCAAAAAGTTTCAGTTTTCAGACGGATTCAGAAGTTTCTAAACCATCAAAAACATGCCCACCACCAACGCATACTGTAATTAATATTGAACAAGATTGTGTCGATACAGTTAGTCATGTTCCATTTAGCTTTATTCAATTTTGAGCATTTTCAGAACTTTCGTTCACCATCCCGAAGTTCTGGAGAGCCGGGATCACCAACAACTTGTGAACCAATGATTCCTCATATGAAACTATAA

>C.ELEGANS

ATGCGGAAAATGCTACTTTTTTGCATCCTTCTACTCTTTATGCCTCAATTTACAGGTAATTTTTCATTTAAAAAAAATGATGTTCTACTTGGAATTGAACGTCACACCTCTTAAAACATATTGAACGGATCGAATGATATTTGACACTACAACCACATGTGTGTTCGTGTTCTACACATTTTTTGTCTAGTGCACTAAACATTGTCATATGGAATAAAAATCGCCACAAACGACTATGAGAAAAGATTGGTTCAGGAGCAGGGAATTTTTTTTCGAAAACGAAAATGAAAAATTTAGTTTGAAACGATATTTTGACCGAAATCGCACTATTTATGTTCACAATTTCCAGTGTGATTCTTTGGAAAGTTGTAAAATTTAAACATTTATAATTTTTCAGTATATCAGCATTTTTCGCTTAATTTTTTTTCCAAAAAATCGAAAAATAAATTTTCCACCAAATACACGAAAAATCTACAGCCCTGTTCAGGCATTTTCTCGATTTTTGCTCTAAAAATACGGTACTGGGTCTCGACACGAAAAGTTTTTATTAAATGCATGTGCGCCTTTAAAGAGTACTGTAATTTCAAACTCCCGTTTTTGCCGAATTTTTAAAACTGGTTTCCGATAAAAAATTGTCTGTTTATTCAAAAACAACTATAAAAGCACACCAATTTTAACAAATCGTAAGAAAAACTTTAAAAAATTGATTAAATTTCCGCAGCAACGAAATTTTGTAATTACAGTACTCTTCAAAGGCGCTCATCTATTTACATTAAATAAATATTGTCGTGTCGAGACCACGGACCGTATTTTTGGGGCGAAAATTGCAAAATTTTGCGTCTGGCGAATAGCCGTATTTTGTGATAATTTCCGTTTACCCGGTTGCAAATTGAAGAGGTCATACAGCAATGCACAGTAGGTGATCGTAAATATTCAAGTACCCGCCAACACCGCCAAAATGTTGTATGCACCCCCTCATAAACACCCTTGACAATACTACCATCGGTATTTATTTCACCTATATTGGCAGCAATCTACGAGTTTACCACCAACTATGTACATACCAGTTATTACATATTCGAATTTAAATGGTCCCTTGAATGGTTTGTCATTAATTATTGTAGATTTTTAAAGAAATCAGGCAATTTGATAAATTGACAAAAAAATGTAGATGTCGAGAACAATAATCACAATACAAATTCAAAAAAAGACTAACAACCAATCTACAGTAGTCTAGAAACAAAGAAATAAAATCGTACTTTTTTTATTCTTCAAGTTTCTTT**CCGTGGATCCTTGAGCTTCTGTACTTTCAAAATTCTAGAACTTCCCGTCTCTCCCTATTCAATGCACCTGTGTATTTTATGCTGGTTTTTTCTTGTGACCCTGAAAACTGTACACACAGGTGTTCTTACCAATGTCTCAGGCATTTTTGGAAAAGTAATATTAAGAAAATTATACATATTTTCTTGAATACGAAAAATTTAA**ATGTTCGGTAAATCGATTCCTGAACGACTTCTAGTCGCATTTGGTATGTCTATAATATCAAAACAAAGATTATTATTTTCCATTCAAATATCAATAAAAGTTTCAGAATCGTGTCTCCCTTCGTGGTTTCGTCAAGAACGTAGTGCTCCCGAACAGCTTCAATCTGCAGAGAATGCAGCTGAAAATAGTGGCTCTGTACCACCCGATACTTCTCGAAATTCTCTAGAAACAAACGAAATAGGTGATGCACCGTCGTCGACTTCGACACCTGAAACACCTACTGAAACTACGATTTCCGAAGCTGGAGACGATGAAAAACGAACTGAAGAGGTTGCAAAAGAATTAAGTAATACTTAAATGTATAGAGAAAATCTCAGAAATTTGTTTTTTTCAGTCGAGAAAGAAGCAGAATATGAGGGTGAATATGAAGATGAAAAGGTTGATGAAGAAGTAGAAGAAGCGTTAAAATATAATGAAGATGCCACTCAAGATGCCACGTCGACTCTTAAACCGGCGGTTCGGAAGGAAATCGAGAAGTTGAAAGAAGCAAAATGCAAAGGTAGCCATTTGATTAGGTCTGAAAAACCTTTTGAGTTTTCGTTCCATTTTTCAGATTTCTCGGTTTTCGGGAAAAACCAACCCTTTTTTTAGTTATTTCAACAAACAGTTTTTGAAAATCTAAAAAATTGAAAAACTGAAAAAAGTTGGAAATAATGTATGTTTAAAAAAACGAGAAAACTAGGTTTCTTAAAAACATGTCAATTTTTATGCGAAAATGTATTTTATAAAATAGGAAAATTACCGAACATTTTGAATTTTCAAAAAAAAAAACCAACAAAAAATTGAAAGTTTTTGATTTTTAGTTTTTTCAAAAATTTAAATTTAAAAACGATTTTCGATTTCAAATGAATGAATTTTTCGATTATTTCGATTTTCAAAAAATAGAAAAAGTTTTCGACGACATCTTTCCATGATTCTTGCTTGTTGAAATAATTAAAAACGGGTGTTTTTTTCCGAAAATCGACACCCTTGCAAATGGATTTATGCAATTGGAGTTCTTTAATAGATTAATGAATTCAGTCTAAAATCTTGAAAAAAAATAATTTTCAGACTACTGTCATCACAACGCGACATGCCACGTGGAAGTGATATTCCGTGAAGATAGAGTTTCAGCAGTTGTTCCTTCTTGCCAGTAAGTCTGGAAACAATTATGAGCAAGTACAGTAATCCTTTTTGCAGTTGTCCACAGGGTTGGGAAGGCACTCGTTGTGATCGTCACTACGTTCAGGCGTTCTATGCCCCAATCAACGGCAGATATAATGTACGTTTGAGCACGATGAGCAGCACGGCGCAACTCCTCGTTCAAGTAATTATAATCTCTCATCTAAACAAAAAAAATTAACAAAAATACCCTTATTGTGAAACCCTAATTAAGTTGCATGTAGCATGTTCTCATCTAATTATTTTTTGGTTTGTTTGCGGTGATGGTTTTCTATGAGAAACATATCATTTTTGTAAATTATTTCTAGGGAAAAACGAAAAAACCATTAATTTTTATGGTTCATCATCCAAATCAAACGATTTCTACAACGCCTTCCAGTCAAGATTCTGAAATTTCTTCTATTTTCAGTGGGCTTTATGAGAGAATTGTGGTGAGAATATAAATTATATAATGGGCTTGATGTCCACATTATTCGTCAATCTAGAATATTCGAAATATGAAAAAAATTAAATTTTAAAATGCTGCATTTGCTCATACTTTTTTTCAGCAATCTTCAACATCAGCTATTCCTGCGTTCGCATTTCTCATTGTCATGCTCATCATGTTTATAACAATTGTTGTTTATGCTTATAGAAGGTATACTTTTCATCAAAAATTTGAAGTCTTGGTAAATTTTCGGTTTTTTTTCTCAGAAAAAATTTCAGGATATTATGAAATTTTGTTCATTTTGATGAGAAAAAACCTTAGAAAATAGCATTTTTCTAAAAGGAGAAAAATGACCGATAAATTGATTTTTTTTTGAAATATTGAAATATCTGAAGAACGAAAAATTGGAAAACGAAAAATTTCGATTTTCAAGTTTAAAAAAATCGAAAAAAATTTGCATACAAAAATGCAATGTTTCGAAAAATATATTTGAATTTTGGCCAGTTTATTCTTTTCTCCTCTCTTCAGAAGAAAATTATGTTAGGAAAATTGTTTTCTAATCAACACACAGCTTAATAATATTTTGAAATTGTTGTTAATTTTCTAAGCATCTTTTTTTAAAGATTTTCGAACATTTTAATGTAAATTGTTCAAAAACAACTATATAAATGTTTCAGAATGTCTAAACGATCGGATGATATGACATATACAATGAGTCATATGTGCCCACCAGAAGCATTCAATGTCCTCAAAACACCAAATGGACGACATATTCCAGTTCATCAAATTCCATCATGTTCTTATACTATCCCAACACCGGGTACAGTACCTCCAAATATATCATCAACTCCTGGATCAAGAATACCCACTCGTCAACAAGCTATTCGAAATAATGAACAAGCACGGAACAACTTTTTCAGCATTCTCAGAAGTCAAGGTACCATTCCATCCAGGTATCAAAATTCTGAAAATTCATTTTCAAAATAAAATGAACTTTCAGGAGTATCAATGACGACGACACGCCGAAGCACTACAAGTCAGTGCCACGTGTTGAAGTTTCAGCAATTAATTACTCTGGCCACATTGATTTTTCAACAGTATCATATCAGTCGGTCAGTTTTTCGATTTTCATGGATTTTTTGAAAATAAATTAATCGCACATTTCAGACTGAATCAGAAGTTTCAAAAGCATCAGTAACATGTCCACCACCGGCGCACACTGTAATTAATATCGAGTTGGATTCTGCAGATACGGTAATTATTGTTTTTAAAAACTAAGAATCGAGGTGATATTACTCAAATGCGATTTTTTCCACGACAAATTTTTGTTGAATGTGGAGTTGTGTCGTTTTGAATGCACTTCAAATGATAAAAATAGACCTCAATGAACAGATATAACTGTTAAAAAACTTTACGAAAGTTTTTTAAATCTTTAATGATTTTTTGGATTTTGCAAAAACTAAAAAAAGGCTAATTATTTTTGAATTACCGCGCAAATGATGGAATGATTTCTGGAAAATATTTTCTAATTTTTCAATATTTGATGTAAACACCGTTTGTCAATTTTTCAGACAATTTCCCGTAAACTTGAAATAACCCCCGAAAGAAATTCGTTGAAATTACTAGTTTGGCAAAAATATCGATAATTTAACTTAAATTAATATTTTTTGCGCGGAGAATTGTCTGAAAAATTGAGAAACAGTAATTCAAATCAAATATTGAAAAAAAAAATTGAGAAAAACCAACGAATTTATTCATAAAAGTGAAAATGTCGAATCCAGGTTAGTCGCAAATTCAAAAATAATTATCCCTCAAATGTTTTTTCATTACTTTTTTAGCAGTTATATCTGTTTATTACGGTCCTTTTTCATTTAGATCACATTTTTAAAAATGACTGTACTCCGCCTTTAACTGCAAGCGGAATTGCGCCTTTAAAGATTACTGTCTTTTGCAACCCCTCGTTGCTACGAAATTTTCATTGATTATAATACAATACATTTTTCGGGTTACATCCATTTGTTTATTTTTTGGAAAATGATTACAGTAATCTTAAAAGACGCAGTTCAACCTGGTATCGTATTATTTGTGCAAAAATCGCAAAATCTAACCTCTGAATAATAAAATGATTACTCATTTGAAAGTTTCAGAATTTTCGATCCCCGTCTCGAAGTTCTGGAGAACAAGGATCACCAGCAACATGTGAACCAATGATTCGACACACATGAaattataatttgtttccttattttgtcttatctttttcttttttcatcaacttctctgtgctataattgtgatttaccactgctcaaacatctctggtctcttcaaattttttcgctttctcttttttcctctattttcatatcgttctacatgttttatagattgcatttggcaccctattatatcccacccaaatgttctatagttctctcactctccctctcagttcctgtgacaatttgtataattttgccagttcaactcctcgatttccaacgggtacaatatttgcatcgtttttgtaatttcctttgatccctagtctcttcaggtcctcgttgaaaaaactaaacatttatttcatgtttttattaatggaaaattgagaaaaatcatatagaaaaaatatatatttagatagtcaataaaatccaggaagtttgcactttttactggcatttttccttctctccgtgaaccttgatgtcgagggtgacgtaggtcatgtcgtaggttcctccgatcttgtcctctggcaccaagtaccttccgatgcggacacagttctggaattattttaataaatatattccattatttattttgacttactggcttagatgggttgcagctatgggtaatcaaaaggtatggggcaatagcatgagtctcatcttcttctccgtaaacgctgaattctccttcagcatcagttttcttggtgtccaaaagatcatctgggtcaactgaaaaatacaaatacatttatagaaaagttctatcaatgaacttacgggtgtccttctcatagagttgaacttcaacatttgccaatctcttcttattgcacacggcaactcccttaacagtaacattctgaagacgggctgtgcattcaagagcacaagcggcgagcaagagacacacgacgagcgagcgcattttcaacaataagttgttgaggtgactggcgtcgtgttggggctcactatttatattgagatttggagattaacgtcaggtgttgtccccccttcaatcaaccaatagaatcaaagaaacgagagccaattggcgtgtccaacagccagccatgagacaacatgagaacttgaatcagaaacaacaacaagtaatcaggg

>C. JAPONICA

ATGCGGAATCTGCTGCTCTTCTGCCTTCTCCTCCTGTTTCTGCCCGATTATGCAGGTACTTTTTCTAATGACATTTGACACGAAACATGTGTTCTGACACAACCACCGTGGTCATATGGTAGTGGTTTTATTGACTGAGAACAGAATATATGCGAAAATAAAAAATAGAGCACGCTAGCAAGACAGGGAAAGTTTTCGTGGCCGAGGAAAATTGCGAAAACTCGGCCACCAAATTTTTTTTAACTTTGTATTTTTTTTTCGTTTTCTTACCTTTTTTTTTTGAAGAAATGTTCAAAATATATGCACATAATTATTTCAGTTTATTTCTAGACGGTTTCTCGAAAACGAGCAGGTAGAATCTTCTGAAAATTGACCAAAACCTTCTCCAGCAAATCCTCTATTTTTTGATATGTAATTTATACTTTTCTATGTCTTCATTTTCGAGAAAAATCACAAAATAGTGAAGAATCTTGATAAAAATTGTTTTTCTCGAAAACAAGCTGGTTGAATCTTCTAAAAATTGACCACAACCTTCTCCAGCAAATCCTCTATCTTTTGATAATTAATTTATAATTTTCTATGTCTCCATTTTCAAAAAAACCGCAAATTTGAGTTTTTTCAAAAATTGTCCCAACGTATTTATCTTTTTTCTATCTTTTTTTTTAATGCCGAAATTTTCTGGTTTTTTGTGGCTATTCAGATACTTTTCCTCATTTTCTTGTTGTCCGAATCTTATTTTATCAATGAATTTCAATTTCCTGTTCATTTTATTTTTATTTTTAATGAAATAAAGCAATTTTGTTGTTGTTGTTGTTAATAACTTCATGGACCTATTCACACAAGCGGAAAAAATTGCGGGAAAAATCGGAGAATGCTCCGATTGGCCTGAAATTTTGGGAAATACTGGAANNNNNNNNNNNNNNNNNNNNNNNNNNNNNNNNNNNNNNNNNNNNNNNNNNNNNNNNNNNNNNNNNNNNNNNNNNNNNNNNNNNNNNNNNNNNNNNNNNNAAAGTTTTCAGAAAAATCGATATTTTGGTCCAAAATTTTTGAGCGTGTACAGGAGAATTTTTTTCCAATATTTCCCAAAATTTCAGGTCAATCGGAGCATTCTCCGATTTTTACCGCAATTTTTTCCGGTTTTGCACGGCGTGCTAGACCCTACGTGAGGAAATTCGCGCCTTTTCGCATGTGTCTGTGTCTCGACAGTGGCATTCTGCCAAGCCTATCAAAAAGTGTGCAAAAACGGAGACACAGAAACGGGAGAATTGGCGCTCGAAAATTTGATCTCAAAAAAGTTTTCTGAAACGTAGTCCTCAACACGCCGTGTTTTGTGAATAGGTCCATTAAAAAGCAAAAAAAAAATCCCAAAACAACTTGGCGATGACGCTAGTGCAACGCTATTTAGATTTGGAAGTCAGCTCGCACTATGCTAATCGCACGCTAGTACAGTGCGCGTCATAAGTATATGCCCACCCCAAATTTTTGATGTTTAGACCAAACTGAACTATTTGGTTGAAAACCGTTAATGCTATTCGATTCAGCATGTCAAATAACCTATGTGGCCAATTTTTCATAACGATACAGTCAATATTACGTGTTCCAGGCGAATAAGTTGAAATTTTGGTCGCGTCATAAGTATATGCCCACCCCGAAATTTTGACGTATTTTCGAAAAAAACATTCATTTTTCTTTGTATTTCATTCACAATTTGATTTTTTTTGTTTTTTTTATCGATCTAGGCGGTTTTCATAGTATTTAATGTTACCGAATATGTTCTCGTTGGAGCAGGGCTCTAATAGTGTTGCTAAAAGCGAAGGTAGCTACCGTAGCCTTATAAAGCCAAAGAGGCGCAAGGAACGCTATCAAATGATGTATTGAAGTGATAAACAGAATTTAAATCAAATTAATTTAACTTCCCATTCGTCTTTCTGCTTTTTCAGTTGATTTTCTCATAATTTTCCAGAATCCTAAACTTTCAATGGAGTTGCGAACATAAAAGCAGCAATTTTTCGACACTGTTAAGAATTATAATTACTGGAAGAAAAGCAGAAAAGCGGCATTTATTAATGATGTGCTTGCGGCATTGACCGCTCCATCCAAAAGACTACAGCCGGCTTCGATGGGCTTTAAATCGCGACTGAAATCTGGCCACTCGCGAGCATTGATCTTCTGACTTCATTCTGTGGGCTTAGTGAGCAGACGTCATGTAGAATGTGATACGAACCGTTATGACTTCTCAATACAATTAATTTTGTAATGTGGACATCGAAGTTCTTGATGATTTCTGTTGGAATCGTTATTTAACTGGTGGTACCAAATCACATTGAGCTTGTCTTGTCCTTTGAGCGTTTTTGGTAGAGCGCATCAAGAGTGTTGTATATTTGAATGAACATTTGACAGATAACTGCATGATACCGCAAGTTCACTAGATTTTTTTTTGTTCTTACGAGTTTGCATAGTCCGAATTTCAACATAATATCCAGTTGACCGTGTTCTCAATTTACTAGATTTGTTACAATTGGTATATTGGTTGGAAAATGCTGAGAAAACTATTGAGAAAATACGAGAGAAGCATTGGAAATTACAACAGACAGTTGTTAATTTTGCTCATCAGTTCGATAAATCACTTGATAGCGTTCCTTGCGCCCCTTTGGCTTTTTGAGGGTACGGTAGCTACACTCGTTTTAAGCAACACTTTCAGAGCTCTGCTCCAACGAGAACATATTCGGTAACATTAAAAATTATGACCGCCTAGATCGATAAAAAAACAAAAAAAAATCAAATTGTGAATGAAATACAAAGAAAAATGCATGTTTTTTTCGAAAATACGTCAAAATTTCGGGGTGGGCATATACTTATGACGCGATCAAAATTTCAACTTTTTGGCCTGGAGCACGTAATTTTGATTGTATCGTTGTGAAAAATTGGCCACATAGGTTATTTGACATGCTGAATCGAATAGCATTAACGGTTTTCAACCAAATAATTCAGTTTGGTCTGAACATCAAAAATTTGGGGTGGGCATATACTTATGACGCGCACTGTATTACGTGTTCCTCGATTTGAGATACCTCTTAAAATGGTGCGCATTCGGGTCTCGTCACGATGGTGTATGCACACTTGTGCATTTACTCGCAAAATTGAAGTTTTTCGTTTTGAATTTTCGTAACTCCATGCACACTTGTGGATTGACGAAAATCTTTACTATCTTTCTTATATATAAAAACCCAGATCGGGATGTCTGTGCGCGTCCGCACTTGCCTTTTTGCAAGAAAATGCAGTGTGGACAAAACGCGAACCCGTGACATTGGGATTTGTATTTATTCAAGCGCACTACCGACTGAGCCATAGAGGACAGAAATCTTCCCGAAACAGAGTGCGTATAAATGAATGAGGAAGAGTGGAACGTAAAAAAAAAGAATGTGGCGACGAAATCGCCAACAGGCTGCGTCAGCAGCCGCGCAGGACTGCGCAGCAGGCTTGTTACTAATAAAATTTTTCTGTGTGTAAGTCTGTCTGTGCATCGGTATGTTCGCACTGCTCGCGCAGTCCCCAGAAGTCCGTAGGGGCCCAAACTTGGAAATCTGAGTCTCCTGAGTCCCGCGATGATGCCCGTCTTTTTCAAATTTTGATATCGTTCTTTTGAAGGGTACTTTACTGTTAGGCCGACCGGTCCACATTTTTTTTTCTTCTGCACTTTTTGCTTTTCTACAGTCGAAAGGAAATCCGACAGAAACTGTGGTTCGGCCGAAATCTCATTGCACACATAGGGAGAATTATTCAATCTGCGCGGTGTCCAATTGGTTTGTAGTCTTGGCTGGCGTGCGCGAGGTCGCGAGTTCGAGTTTCACTGTGGTTTTTTTCTTTTTTGTTAGGGATATTTTGTTTAGTTAGTGGAAATTTAGCCGACAAAGTTAGATTCAAGTACGTTTGTTATTATTTTTGAGCTATTTTTTGAATATTTCTTTTTTGATTTGTCGTTTGAAGTGTATCTTTTATTTTCATCCATACTTTTGTTTTAGTTTCTGAGCGGTAATGAACAAAATTGCGCCAATAGTTCTGTTGTTTTGATCAAATCCAAACAGTTGGTAATCATTTCGAAACTCTCCATTCAACCCAAAAAGTTTTGTGGAAACCAGAACAGGTGTAATGAAGAATTAAACTTTTAACTTAAGATCGGGTTCAGAGGAAAATAAATAATGGGAGAAATAACGATTCCAGATACCGTAAATTCTCTAATAGAACGACCGGCCGTTCTATTTTTCACTACCCTCCTAGGAGGGCCGGTCTATTTTAGGGGGCCGTTATATTATAGGGTTAGGTTTACTTTTCAATGTCTTTAAATTTGTATTAAATCTTGAGGATTTTCCATAAAAACTACACAAATGTATGACAAAACAAATTTAGGAACCGGTCAATAAGTAACAATTGGAAAAATCTAACCAAATTATTGAAAAAAAATGTTAAATTCATTTCTATATGTCAAAAAACGAATCCTATGAATAGGTCCATTAAAGAGTAAAGATGACTTTGCTTTATGAGTTTTTATAATGGATTCGTGACGGAGTGAAATTTGACGTGCAATTGGGAGACAAACAATCTCTTTGGTTACTTTTTTTTTTTCGAATAAAAACATGAATTCAAATGTCACTCCTCCTCGAATCCATTATAGTATGGGAAGACGAATTAGAATATTCAAATGCCAGGACTCGACCTGAAGAATTGTGTTCAGTTTTAAAACATCTGAGTACGGTAGTATTTTTTTCAAATTTTCTTGCGCAATGCATCTGGTTGAAAACATCAAAAACTGTTTTTTTTGCTGCACTGAATAGGTTTGTGACATTTTTCTTTTTGCAGGCAATATACAAAAACGGATTGACAGGCACACAGAAAAACTTTGAAATTTTAATTGATTCAATTTTGGGTACTTACTCAATGTTTCTTTTGGCATACATTAAAACTGTTTGAAAAATCAGGGAGACTCGGGCATTCCCAAGCTTACCCAGAAGGTTTTTGACGGCGATAGCCGTCAACAACCTTCTGGTTCAAAACGAAAAAAATTAATTTTGCGCGTAAATCGACAAGTGTGCATATAACATCGTGGCGAGACCCACCATTTTACGAGGTATCTCAAATCTAGGAATACGTGATACTAGCGTGCGATTAGCAGTAGTCTGGGCTGAAAACACTTCCAATTCTAATTAGCGTTGCACTAGCGCCGTCACCAAGTTGTTTTGGGGAGTTTTTTATAATCACAAATTGATAAATACGTAATAATACGCTATCAGAGTATTACGGACTTATGTACGAAATAAAAAAGTCCATTTAAATGAATTAATTTGACATTTTTTTCTAATGCACGAAAGCAAGAAAATTCGATTAAATTTAATAAATTTGAACGCAGAAAAACGTTCGTGTCGAAATCTCGTAAATCGACACAATTTATTAAAGGCGTACGCAATTTTTCAATGACAATTTCTCGACACGAACGTTTTTTTTTCTTGCTTTCGTGCATTGGAAAAAATGTCAAATTAATTCATTTAAATGGACTTTTTTATTTCGTGCATAGTTCCATAAGAAAATGTGTGATTGGCATTAGCGTGCGATTAGCTTAATATAGTTTTACCCTCCATGAAAAAGCCTTTTCACCATGTTTGCAAAGCTCCAAAAGTAATTTGAGCTAACTTTTTATGACCCCCATCTATTCTCTCCCCAGTAGGAGGCCGTAAATATTCACAAGTGGTACACGATAACCATGTACTACCGCCATAAACACTCTGCATACACATATTCGCACCTTGCCACACTTTTATGATTCCACTTTGCCCCCACCACCAAATTACGCCAAAACTATTGCAATGTACATTCCGATAGTCAGCTATACCGATTTTGACAGTACTGATGACGTTTATCGTGAGTTTTTTCTTAAAAATGGAGGAGGGAATTTTTGCGCAGTCTAGGAGCTATTTTTGATGGCTAATAGCCAATAATAATATGATAGTAGGGAAAAACTCTATTAGAAGGGCATGCACTGCCCTTCTAATAAAAGAAATACGGTATGTGGAACGCCTAAGCCTTGAAATGTTCAAAAATTACACAAGTTTCCAAACACCTGGTGACCTTACTGTTACTGTACATATTTCCACACAGGTGGTTGTACTTTTTCGCGTCGCGCACTTGCGATATTATACTTCTCAAGAAAATTTTACTAATTCTACTACTTTCTTGAAAAATTGAACGTTTGAAAATGGCAACGATTCCAATTAGGATTTTGGTGGCGCTAGGTGGGTGAAAGTTATCTTTGCGATGAGGAATTTAAAGAAAATTTAGAAATGCGGTGGATACTGCTCGAAAAATATTCGAAATTGCATGAAAATAACTAGAAATTCCATGCGGAAATATTAGGTGACACCCCCAAACAGTCTATTGAAAGTTGGGACTAATACAACAGTCTGTGAGCAGTTAAAACAAGGTTAAAATTTCTACTGATATTGTATTCTAACAACAGTCTACTGGAAGAAAGTTAACCTGAAAATGGAGAGGAAGTACGCTTTTTCCACCCCATTTTCGAGTGAACAGAGACTGTCGTTAAAATAGAATACCAGTAGAAATTTTAACCTTGTTTTAACTTCTCACAGACTGTTTTATTAGCCCCAAACTGTGCAAAAACTCTGAGCAAATTTTTTTGAAAATTGTCGTAAAATTAAATGCCGCGTACGCAGACACTTGAATCGACGCGCAAAGTCTTCGTCACCAAGAAATACGTTTCTGGACAAAAATGAAGAAAAACAAATTAAATTAAAGAAAGTGTCATTCCGTGGCGAATGCGTAATATTTCGGGGGGGAAATTGTTTTAAAATTGTCGTAAGGCTATTTTTGTAGCCAAATTTTTTTCGAAGGGGCCTCACGCCGGCCTAGTAAGCCTGCTGAATTACAAACCATACTCTACTGAAAAGCTGATTCCAAAAAATATAATGAAGAATTTTTAGCGAGCAAATCTCGATTTTTCGCCAAAATTTTTAATTTTTAGTCATTTTTTAATGGTTTATTCAACAAAAAAAAAGGAAAAATAGGGGGAAGGCTCTGAAAATGAAATGCTCTTATTTGTCTGAAACATAGGAAATATTGGGAAAAATGCTCCCGGACCAAAATATAGATTTTTCTGAAAATTTTTAAAGATAATTTTGAACCGAAATATCTTTAAAAATGTTCAGAAAATCTATATATTCGTCCCGGGAGCATTTTTCCCAGTATTTCCTATGTTTCAAACCAATTTGAGCATTTCATTTTCAGAGTTTTCACCCCTATTTTTCCTTTTTTTTTTGTTGAATAAACTATTAAAAAATGGCTGAAATTTAAAAATTTTAGCGAAAATTCGATATTTGCTCGCTAAAAATACCTCATTATATTCTTTGGTATCAGCATTTTAGTAGAGTATGGTTTGTACTTTAGCAGGCTTACTAGGCCGGCCATTTCACAAAAAAAAAATGCTAAAAATGACCTTCAGAAATATTACGCATTCGCCAAGAAATGACACTTTCTTTGATTTTTTTCCTTCATTTGCGCGTCGGTGCATGTGTCTGCGTACAAGGCATTTAATTTTACGACATTTTTTAAAAATTTATGGAAAATTACTAGACAGACAACAGTTTACAGCCAGTTAACTCGCATTTTCCCTATACCCAAAAAACCGCCAAAAAGCTTCAGAATCATGTCTTCCTTCGTGGTTTCGACAAGAACGTAATGCCGAGCCACTGCAGCCGGACAGCCTCGTCGACCCGTCTGGCACCAAAGACTCGCTGGAAACCAACGAAATTGGCGACCCGCCAACTACTGAAACTACTGAAAATCCGTGAGCATTTTTCGCCATTTTTTATTGGGGAAACGGTGATTGCGTACTTTAAATTCTCAGAGATCCTCTCCCAACGCCTCCAGACTTTGATCAACACGAGACGAAGAAGATGAGAGAGAAAAAGAAAAAGAGAAGGAGCTACAAGAAGCCGTCAAAGATATCGGTATTTCACCCCCAACCACCATTCAATTTTATCCAATTTCTCTTGAATTACAGTCGAAAAAGAAGCCGAATACGAGTACGAGTACGAAGAAGAAAAAGTGGACGAAGAGGTGGACGAGGCGCTGAAATACAACGAAAAAGCGACGAAAGAGGCGACGTCGACGCTGAAACCGGCCGTGCGCAAGGAGATTGAGCGGCTAAAAGAGGCAAAGTGTAAAGGTGACGAAAAATATGACTCTCGGACATTTTGAACATTTTTCTGCATTTTGTCGTCGCGGTGTTTGCACAAAATCATTTTTTTTTCATGGGAAATTCCATATTGTGTGCAAACACCGTGACGACAAAATGCAGACAATTTTTTGACAATAGATCACAAATAACCGACCAATTACAGATTACTGTCAGCACAATGCGACGTGCCACGTGGAGGTGATATTCCGCGACGATAGAGTCACGGCGGTGGTGCCGTCTTGCCAGTGAGTCGTCTTTTTTTGTTGCCGAAACTGCGCCAAACCTGTGCCGTTTTTTCCAAACTCGCGCATATCCCGCTATTAGCCACATATCATACATGCCGCATAGGCGCCGAACCCGCGCCAAAACAGTGCATAACGCTGATTAAGTGGCTATTAGCTAAATAGCTGTCATATCCCTGCCAAAATTGCATAAATTCCGCACAGGCGCCGAACCCGCGCCAAACCAGTGCCGAACCTGCGCTAACCCAGTGCCAAACCATCGCCAAACTTGCCCTGGTTATCGCCAATCAGCTGGCTACTAGCTGTCATATCCGCGCCAAAGTTTCCTTAATAAAAATGTATTTTGGTGGATTTGTTGGCTATTAGCCGTCGATTTTGGGGTGTCACCTCAATTTCGAGGGCTAACTCCCGCTTGTGTGCCGATTGCCGCCATACTCGCGTCGAAACTGCGCCAAAGCTGTTGCAATCGTCGATCAGCTGGCTATTAGCCACAAAGCTGCCCTAACCTATGCCAAAATTTACATTTTGGCATAGATGCCGCATAGATGCCGCATAGGCGCCAAGTCCGCGCCAAACCTGTGCCGATTGTTGCCAAACTCGCGCTGATAGTCTGCCGTTGGCTATTAGACACATAGAAGCCATATTCGCGCCAAAATTCCAAAATTGGCATACTAGCCGAACCAAGCGAAAAGTGCCGAATACGCGCCAAACTTGTGCCGATCATCGACAAACTCGCGCCGATCGCCGATCAGTTGGCTACTAGCCACATAGCTGCCATATACATACAAGCCATAATTACAAAATTGGCATACATGCCGGATAAGCGCCGAAGCCGCGCCAAATATATGCCGATTGTTGCCAAACTCGCGCCAATCGCCAACCAGCTGGCTATTAGCCATATAGCTGCCATATCCGCGCAAAAATTCCAAAATTAGCATACATGCCGCATTGGCGCCGAACTCTTGCTAAACCTGCGCCGATTGTTGCCAAACTCGCGCCGATAGACGATTAGGTGGCTATTAGCCACATATCAGCCATATCCGCGCCAAAAGTCTCAAATTGGCATGCAGGACGCATAGGCGCCGAACCCGCGCCAAACCAGTGCCGATTGTTGCCAAAGTCTCGCCGATTAGGGGGCTACTAGCCAAATAGCTGCCATATCCATATCGAAATTCCAAAATTAGCATGCATGCTGCATATACGCCGAAACTGTGGCGAGCCTGTGCCGATCGCCGATTAGCAGTTTATTTCTATGGCTATTAGCCACACAGCTGCCATATCCACGCCAAAGTTTCCAAGTTGGCATACACGCCGCACCGCCGCCAAGTCAGCGCAAAATCTGTGCCGATTGTTGCCAAACTCGCGCCAATCGCCAAACAGCTGGCTATCAGCCACATAGCTGCCATGTCCGCGCCAAAATTCCAATATTGGCATCCCGCCGCATAGGCGCCGAACCCGCGCCAAACCTGTGCCGATTATTGCCAAACTCGCGCTGATAGTCAGACGTTGGCTATTAGCCACATAGCTGCCATATTCCCGCCAAAATTCCAATATTGGCATCCCGCCGCATAGGCGCCGAAGCCGTGCCAAATCTGTCCCGAATATTGCCAAACTCGAGCAGTTCGCCAATCAGATGGCTATTAGCCACATTTTAGCCATATCCGCGCCGAATTTCCAAAATTGGCATGCATGCCGGATAAGCGCCGAAGCCGTGCCATTTTTTCCAAACTCGCTAATCGCCGATTAGATGGTTATTAGCCAAGCAGCTGCCATATACGCGCCGAATTTCGAAAATTGGCATACATGCCGGATAAGCGCCGAAGCCGCGCTGATAGTCAGCCGTTGGCTATTAGCCACATAGCTGTCATATCCACGCCAAAGTTTCCAAATTGGCATACACGCCGCACAGCCGCCAAGTCCGCGCCAAATCTGTGCCGATTGTTGCCATACTCGAGCCGATCGCTGATCAGCTGGCTATTAGCCAGATAGCTGCCATATTCGCGCCAAAATTCCAAAATTAGCACACATGTCGCATAGGCGCCGAACTCTTGCTAAACCTGCGCCGATTGTTGCCAAACTTGAGCCGTTCGCCAATCAGCTGGCTATTAGCCACATAGCTGCCATATCCGCACCAAAATTCCAAAATAGGCATATACGCCGCATAGCCGCCGAACTTCCGCCAAAACTGTGCCAATCGCAGAATTTATGACTTTTTCGACGGCTACTAGCCAAATAACTGCCATGTTCGCGCGAAAAATCTAAAATTTTCATAAATGCCGCATAGGCGGCGAACCTGTGCCAATTCAGCGCCGATTTTCGCAATTAGCCACATAGCTGCGATATCCGTGCCAAAAAGTCCTGGTGAATGCCTGTTTTTGAGGAATATCACATTAAAATTGCGTAATAGTTTAAAGTGAGCGAAAATAACGAATTAAAGCGGTAAATTGGTAAAAATAGTTGAAAAATGAATTTGAAAATGCCGCCACCTCGTGTACTCCTTTTGGACGACTTTTAACTTTTTTTTTTTTGAGCGATTGTGTTATTCATTTGTAAATCCTTCATGGAAAAGTACATTTTTAAGCACTTTTCCGGAAAAATGTGCCAATTTTCAATTTGCGCGGCATTTAACTTTACGGCCAAAAAAAAAAGCGCGTATGCAGACACGTGAATTGACGCGCAATTTAATACGAATGTTGAAAAAAAAAACATTGAAATAACAAGAAAATGTCGTAAAGTGAAAGCACGCGTATGTAGACACGAGATCTGACGCGCAAAAATCGCAAAATACAAAATAAAGGCTCCGCCCCTTTTGCAAATTCAATGCATAGAGACTTGCAAAAGGGGGTCTAAAAATGTAATGCAACCTACTCAAAACCTCAATTTTTCAGCTGCCCACGTGGCTGGGAAGGCACCCGATGTGACCGTCACTACGTGCAGGCGTTCTACGCGCCCGTCTCTGGCAGATATAACGTACGTTTGAGCACGACGGCGCAACTTTCCGTTCAAGTAATTCTCTATCCTATCTCTCTACCTAACGTACTAGCTGACCCCTAATTGCTCCCAATTAACCAACATAACACCACGCCCTGACGGCCTAGAAAATTTGAAATTTGAAAAAAACAACTACACAGCTAAAACGAGTGATACCGTAGTCATGAATTTTTTTTTTACAAATTTCAAGTTTTCTAGACCTTAAAAACTAAAATGTATGCATGATCCTCTTACCCGGTTTGCAATGAATGAAATGAATGAATGCGGTGACGGTTGATTTTCGAAGTTGTCGGCATCTTTTCCAAATTTTCTAGGCAAAAACCGGAAAACCGAGGCTTTACCTGGCAAAAGTGCCTCAAGATTTGACAAATTCAACGTCTACAAATGCCACGTCGCCAGTTTTCAGCGATTTTTACGCGCAAATTGTGGTGAGAAAAAACATTCCTTGACCATTGGGGGGCGTAGAACAGATTTATCACTGACAAAACATTGACCTCATAAAGCTCCTTGAACTTTTCTTCCAGCAAGGTTTGTCGTCGACAGTGCCCGCCTTTGCCTTCCTTGTCGTCATGCTCATCATGTTTATAGCGATTGTCGTGTATGCGTATAGAAGGTGAGCGTTTTTAGCGATTTGTGGGCAAACGTTTTGTTACGGGCCTTTTACAGACCTAAAAATGTCCTGGCGTGTCCAAAAAGCGCATTTTCATGTTGAAAAATTGCCTTTCTAGACATTAAAAGGCCGATTCCTGGTCAAAATAGGCCTTGCCAAGTCTAAAAAGTTCCATTTCAGGACAAAAATTATGCTCTGCCAGGCCTAAAAAAGGGATTTCCGGTACTTAAAATTGCATTCCAGTCCTGAAAAATATTAATTCCCAGCCAAAAAAGTCAGGCTTAAAAAAGGCCCACTCCTGGCCGAAAACCCCCATTCCAGGCCTAAAATAGGCTTTTCCAGGCCACAACTTCCATTTCAGGCCAAAAAATGCCTTGCCAGGCCTAAAAAAGAGCCCCTGCCCTTCCTAAATAGGCCCTTCCAGACAAAAAAAAAAGATGTCCTGACAGGCCTGAAAATGCTCTTTTCAGGGCTAATAAAGGCCCTTTATCCAGATAAAGAAAGAAATAAATTTTTGCCTAAAAACGGTTTTTTCCAGGCCTAAAAAATGCCCTGCCAGGCCTAAAAAGGCCCATTTCTGACTGAAAATAGCCCTGCCAGGCCTACAAATGACCCTTTCTAGGCAAAAAACATTCTCTGTCAAACCTGAAAAGGCCTATTTCAGGCTATAAAAGATTCCCTGTCCGGCATAAGAAAGGCCCTTTACAGACCTAAGAAAGGCCCTTTTCAGGGCGAAAATTCCGTTTCAGGCCCGAAAAAATGTCCTACTATACCTAAAAAGAGTCCTTCCCAGGCCTAAAATAGGCTTTTCCAGGCCACAACTTTCATTCCAGACCAAAAAATGCCTTGCCAGGCCTAAAAATACGCCTTTCAGGACTATGAAAGGCCCCTTTCTATGCAAAAAACTTTCCATGTCAAACCTGAAAATGCCTACTTCAGGCCTAAAAATGCTCTTTTCAGACCTAAAACCTTTTCAGGCTGAAAAAGATTCTCTGTCCGGCATAAGAAAGACCCTTTACAGACCTAAAAAAGGCCTTTTTAGGCTTTTCCAGGCAAATACTTTCCATGTAAATCCTGAAAAGGCCTTTTCAGGCTTAAAAATGCCTTTTCCAGGCCTAAAATAGGAAAAATGGTTTTTCAGGCCTGGAAAAGGCCCATTCCCTATAATTCCGTTCCAGTTCGAGACTCGCAATTTTCTGAAATTTTTCCTATACCTAAAAAGGCCTATTTCTGGCCTAAAAATGCTCTTTTCAGGCCTAAAACAGGCTCTTTCTAGGTCAAAAATGTACCCTGGCTCATTTCAGGCCTGGAACAGGCCCATTCCCTATAATTCCGTTCCAGTTGGACACTCGCAATTTTCTGCACTCTTTCCTATATCTAAAAAAGGCCTATTTCAGGCCTAAAAATTCTCTTTTCAGGCCTAAAGTAGGCGCTTTCTATGCCAAAAAAATATGCCTTGTCAGACCTAAAAAAGGCCCTTTCTAGGCAAAAACATATTCCCTGCCCCGCCTCGAAAATGCACATTTCTGGCCGAAAATAGCCCTGCCAGGCCTAAAAAACCTTTTCGGGCTAAAAAGATACCTTGAAAATTCCATTCTAGATTAAAAAAAAATGCCGTTACAGGTCTACAAGGAAATTTCTAGGCCGAAATAGGCTTTTCCAGGCAAAAACATATTCCCTGTCTCGAAAAGACCCATTTTTTTCGGCTCTGGGACGTTTCAAAACAAGACGTTTCGAAACTGGGACACTTCAAAACTGGGACGATTCAAAACCTGCCACTTCAAAACTAGGACGGTTCAAAACTTTGAGTGCAATTGTGCGTCAAGGTGAACGCACTAGTGACACCAGAACACATGCACATTAAATGTTTATTTTCGTTTTGTCATTGTTTTAACTCTCTTCACGCGTTTTCCTCTCGTTTCGCATTTTTTAAAATAATTTTTTGCCGAGCTCATGAGCTCGGCGAATTTGACCAAATTTAAATACCGGCAAAAATTATTTTTAAAAAATGCGAAACGAGAGGAAACCGCGTGAAGAGAGTTAAAACAATGACAAAACGAAAATAAACATTTAATGTGCGTGTGTTCTGGTGTCACTAGTGCGTACACCGTGACGCACAATTGCACTCAAAGTTTTGAACCGTCCTAGTTTTGAAGTGACAGGTTTTGAATCGTCCCAGTTTTGAAGTTTCTCAGTTTTGAAACGTCCCAGTTTTGAAACGTCTTGTTTTGAAACGTCCCAGAGCCTTTTTTTTCACCGTCTCGAAAAGACCCATTTCTGGCCGAAAATAGCCCTGCCGGGCCTATAATAGGCGCGTTCCAGGTCAAAAAAAGCCTGCTTGGCCGAAAAAACGCCCTGCCAGGCCTAAAAAAGGCCCATTCCAGGCCAACAAAAATATTTTCCCCCAAACTCCTCATTTTTCAGAATATCAAAACGCACAGACGACACCACGTACACCATGTCCCACATGTGCCCACCAGAAGCATTTACTGTCCTAAAAACGCCAAACGGCCGAAAAATTCCCGTCCACCAAATGACGTCAATAAACCCCGCCGAACCGCCCGCCACTTCATCTTCAACCCCGGCGGCGCGGGCTCCAATGCGTCATCAGGCGGTGCGAAATGTCGAGTCGGCGCGGAGCAACTTTTTCAGCATTCTCAGGAGTCAGGGCACGATTCCGTCGAGGTGTGTATTGGGGTGGGGTGGGGAGAAGTGGGCGGAGCTGTGATCAGACGTCTTAAAATTACGGTAATTTCAGAAGTATAAACGACGACGACACGCCGAAACACTACAAATCGGTGCCACGTGTCGAGGTGTCCGCCATCAACTATTCGGGACACATTGATTTCTCGGCCATTTCTTATGTAAGGGGCGAAACCACGCCCACTTTTTTGGCTCATCATTTTTTGAAAAGCTTTTAAAAATGATTGTGTGCAAACACCGTGACGACAAAATGCAGAAAAAATTTCGAAACGTCCGACTTTTGCTCACGAGGTGATAGAGGAGGCGCAGAGAAAAATACAGCCTTTTTGAGCTATTGGCCAAAAAAGTGGGCGGGGTTTCATTGAAAGCACAATATTTAATTTTCAGAGCTCGCCAGAAGTGTCAAAAGCGAGCCTAACGTGTCCACCGTCACCGCACGTTGTGATTGAGATGGATGAGCAGGTTAGTTGATGCCAGATGTGGCCTAGAAAATGAAATGAAAACTCTTCCACGTGTACTTGGACACAGCTAAAATGAGTGTGACCGTAATCAATCTATTAAAACATTAAATTAGAAAATCCAGAAAAAAATACCAGATTAAACAGTTAAAGTCGCTTAAAAATTTTGAATGGAGGATTACGGTAACACTCATTTCAGCTGTGTCGGATCGCGTTGAATTTAATCGTGGAAGAGCTTGAATTTAATTTTCTAGGCCTATTTATTCTCGCAAACTCCGCCCCCTTTTGTTGTCCTTTTTTTTTCGCAGTAAGAGTCTGCAAACACCAACGTCCTATTTTTGCTTCCGTAGGCTCCGCCCACTTTCTAAACTACGGTATTAGCATCCCAAAAACTAGAAAAAAATAATATTTTTTTAGCGCCATATCTTCTGAGAGACAAAAAAATGGGCGGAGCTTATCTGGTTATGGTATGTGATGAAAGTCTGCAAACACCGAGCGAACCTGAATTGGTGTTCTAAGGCTCCGCCCACTTTCTAAACTCCCATGTAACCGGCTTAAAAACTAGCAAAAATTGGTTTTTTTTTTAACGCCAAATTACCAAAAATGGGCGGAGCTTATCTGGTTAAGCACCTAGAGCCCGTTTCGGCTCTGCCCACTTTTTAAAAACGGTCAAAACCGGGGAACTAGCTCTGCGCCTTTTATTTATCTTCGACACTCCTTTTTCTGGCTCTGGGACATTTCGAAACTAGGACGATTCGAAACTGGGACGTTTTGAAACTTTAGTGCATTTTTTGCGCTAAATTAAAAATAGCAATACAGTTACGAAACGTCCCAGTTTCAAATCGTCCTAGTTTCGAAACTTCTTGCTTCGAAACGTCCCAGAGCCTAAAAATGCACCAGTTTCGAAACGTCTCAGTTTCGAAGTGACAGGTTTCGAAACGTCCCAGTTTCGAAGTAACAGGTTTCGAAACGTCCCAGTTTCAAATCGTCCTAGTTTCGAAACGTCCCAGAGCCTGAAAATGCACTAGTTTCGAAACGTCCCAGTTTCGAAGTAACAGGTTTCGAAACGTCCCAGTTTCAAATCGTCCTAGTTTCGAAACGTCCCAGAGCCTGAAAATGCACCAGTTTCGAAACGTCTCAGTTTCAAATCGTCCCAGAGCCCTTTTTCTGTGGTTTTTAGCTCTCCTTCCACAGAAATCGTCTACCTGACTAAAATTGCAAAAGGGGCGGAGCTTGTTCTCCGGGTTTGTCAGAATCTTAAAATTTTGATTCATTTTATCAGGTCTGAACGCTTCAAAAACTAGTAAAAATTGGTTTAGTTTTAAGGCCAAATGATCGAAAATGGGCGGAGCTTGTTGGGTTATGGTAGACGATGGAAGTCTGTAAACACCGTGGGCATTACAAATGCTTTTATTGTTTACTATTGCCCCGAGTGGGACCGTAATCCATTAAAAAAAATTTTGACAGTGAGAGAGAGAGAGACAGAGAGAGAGAGAGAGAGATATCAAAAAATTTTTTAAATGGATGATTACGGTAACACTCATTTCAGCAGTGTCGGATCGTGTGGAATTTTCAAATTTTCAGCATTTCCGCTCTCCGTCACGAAGCGAAGGTGAGCCGGGCTCGCCCACCATATCGGAGCCAATGATACCGCCGCCGCCGACGTAG

>C.AFRA

ATGCGCAATTTGCTGCTCGCCTACATTCTTCTTCTCTTTTTGCCGCACGCTGCAGGTAATCGTTTCATTTTCTGAGCTATCCTGAAAGTTAGAAGTAGGTCAAAAGTGCGTTCAACTTTTTACGCTTCCTGACATAACCACAAGAGCACATATTGAAAGAATGAGGAGGTTAGTGAAGGTGAACACATGTTCTTCTCTCTCTTTCTTTCTTACTACTATCCTAACCACTATCATATGGCTCCTCCGTGAGCACTTGTTTTATTGTCTGAGAGAGAGCAAGTGTATCGAATTTCACACTTTCACACTCACCTCTTCCGAACGCGGGGGTGAGAGGTAATTGAAAAGAGACGTCCTTATCCGGTTCCGTTGGAAATGAGGCGGGGGCACAATTGGAGAGGTGTCATAAATATTCACAAGTGGCGGGGTGTCTCTACCCTTGTTCGGCTACATGCCGCCACCGTAATACGCGTATCTGTCTCTCGCGGAATGACGCCCATAAACACCTTCTACAACTACAACTACAACACAACTCCGAACACCCGCGCGCGCGCGCGAACACCAAGTTTGAACTTCCGTGAACCGGTTATGTACATTCCGCTGATCCGATACAGCGATTTTGAGGTGGACGGAGCAGCAAACGGTTGGTGACATTGAGCTTTTGGTTGTCGTCGGGGGAATCATGACGTGGCACTACCAT**CTGTTTTCGTCGTAAAACTGTACTTCTCTTTCCGGGATACTTCCGCGCAAGGCCACCTTTACTATAACTGTCCGAGAACACCTGTGCGTGCTTTTGCGCGCGCGCGCGCTCACGAGTGAAATGTTTCTTATTTTCCCCTTCCGGACGTCCCTGAACACGCAAACAGGTGTTCTCCTCGGCGCGCGATCTTTCGAGAAGAAGTTTCGTGTTCCACCTTGAAATAGATTCTCTAAAGCTTACATCACTATGAAAACT**ATGGCAACGATTCCTGAACGTCTTTTGGTGGCACTTGGTGGGTTTTGGAGTGTTTTAGTGGCGTCAAAATGACGTGGCACCTACAGTAGTCGGTGGTGGTTTCTCACGTCTCCAAAGTTTCAGAATCGTGCCTCCCGTCGTGGTTTCGACAAGAGCGCAGTGCTCCCGAACCTCTTCAGGCTTCAGCCGCTGACTCGTCACTGTCGGATGCCGCCGACGTCGTCGCCACCACGTCGCGAAGTCCGCTGGAAACGAACGAGATCGGCGATCCATCGGCGCCGTTGGAATCGGATCCGGAGGATCCGTTGCCGACGCCTCCTGAATTGTGAGTTTCCCTCTTCCGCACGGTGCATCGATAAACTTTCAGACAAACGCCTTCTCCATCCGAATCGAGTACAGTAGCGGAAGACGATAAGAAGCTAAAAGAACTCGGTAAATAGCCACGCTTTTTTTCCAGTCGAATAGTGTAATTTTCAGCCGAGAAAGAAGCCGAGTACGAGTACGAGTACGAGGAGGAGAAGGAGGAGGAGGAGGCCGCCGAAGCGCTCAAGTACAACGAGAAAGCGACGAAGGAGGCGACGTCGACACTGAAACCGGCGGTCCGGAAAGAGATCGAGCGACTCAAGGAGGCCAAGTGCAAGGACTACTGTCATCACAACGCGACGTGCCACGTGGAAGTGATCTTTCGCGACGACCGCGTCTCGGCGGTCGTTCCCTCTTGCCAGTGAGTTTGCGCGAACGTCATCTGTCGTACTTTAGCCGTGATTCGCGTGTTTCAAACGCGCCTCCATTGAACGCAAGCCAAATTTCCAGACGGCGGCGAAATTTTTTCCTGTTTCCGCCCGTTTTTCCTCGATTTTCACCTTTTAACACCTTTTAAGGCAGCTCCTCAACATTTTCATTCAATTTTCCCCAAAAAAATGGAGCGAAGTACTGACGTACAGCGGGGCGCGTTTGCAACACGAATCACGGCGATAGTAGACCGACGGAGCTCTCGCAACCAAAAAAATTCAATTTTCTCCATTTTCAGTTGTCCGCACGGCTGGGAGGGCACTCGGTGTGACCGTCACTACGTGCAGGCGTTCTACGCGCCCGTCTCCGGCAGATATAACGTACGTTTGAGCACGACGATGGCGCAACAAGCTGCCCAAGTAATTGCCACCCTTTAATTCACACTAAACAAAACTGGAAAATGATCGCACTTAACTTGCTCCCGCTCCCACCATTTTTCCCCCTCTAATCCCTTCTGCATGCCCCCCCCCCCTTCCTGAATTGATTCTCTCGGTTGTCTTGAATACTGTTTGTTTGCGGTGACGGTTACCTCATTTCTCTTCTCATTCGCCCCCTATTTTTCTAGGAGAAATCTGGGAAATCCAAGGTTTACGTGGTGAGACCACCACCACCCCCCGGCTTCCCGGAATCGACGACCCGCCGCGCACCCAGTGGCTCCGATTTTTCCGACCTTTTCAGCGGTTTCTATGAGAGAATTGTGGTGAGACCTAGGCGGGAGCTCAATTTGGCGGGGAACTAGTAAATTTCACCAATTTTCCCCGCGCACTTTGGAAAGTTTTGTGACTCGATTTCAGCAGTCTTCGACGTCGGCGGCGCCCGCGTTCGGCTTTCTCATCGTCATGCTCATCATGTTCATCGCCATCGTCGTCTACGCGTACAGAAGGTATCACCGACGACGTCGCGTCCCTTCTCACTCCCGATTCCTCCGAATTCAGAATGTCGAAGAGGGCCGACGAGACGACGTACACGATGAGTCACATGTGCCCGCCGGAGGCGTTCACCGTTCTCAAAACGCCGAACGGACGTAAGATTTCGGTGCATCAGACGTCCGCGCCGCCGACAATAACCCTTCCGGCACCGCCGTCCGCGTCTTCCGCGTGTTCTGCCGCGCGGATACCGATGCGGCAAGCGGCCGTGCGGAGTCAGATCGCCGAGAATGCGCGGAACAACTTTTTCAGCATTCTCAGAAGTCAGGGCACGATTCCGTCGCGGTAAAGCCATTCTGAGATGCCTGGACAAAAAACTCGGTCATTTCCAGAAATCTCAACGACGACGACACGCCGAAGCACTACAAATCGGTGCCACGTGTCGAGGTGTCGGCGATCAACTACTCGGGACACATTGACTTCTCGACGATCTCGTTCCAATCGGTACCGTCTGAAAGTGTTCGACGTTGACGTTGAAAATGTGGAAAATTTCAGACAGAGTCAGAAGTGTCAAAGGCGAGCGCCACGTGTCCGCCGCCTGCCTCCACGTCGGCGTCGTCCTCCTCCTCGCACGTCGTCATCGAAATGGACGACGTCGTCGAGCACCACTTCCGATCGCCGTCGCGGAGCTCCGGCGAGCCCGGCTCGCCGACGACGTGCGAGCCGATGATTCCGCCGAAAAGCAGCTAAtctgttcatttttttttgctaattcccttccccctacccaccgtctcctatcaaagtactgtgctataattgtgatcctcctcctcctcccgccgtccaaattgtgccctggtctcttctgaattcaatcaaatgttttattgctccccccccccattttttgttgttcgattccttctgatcctgtgaaaatttgtaattttaataccgcccctctctctcgcttccctcgaaaattgaaattgagaacgaaaaatcaaaacaaaacgggttcg

>C.KAMAAINA

ATGCGGAACTTTCTGCTTATTTGTATTCTATTACTTTTTATGCCCGAATGTACAGGTAATTCGTTTTTTTGGTATCCAAGTGACGTAACGAACATATTGAACGGATCGGATGATGATATTTGACACGACACCACATGTGTGTTCTACACGTTTTTTTCCTTCCTAACCACTGTCATATGGAATAAGTTGTCCTCTTTTTTCACCGTTGAAATCACCACAAAACGGCTCTGAGAACACCCAGGGGGGAAGGAATTTTGGGAGCTCGAGGAATGTCAAGTCCTCCCGAAACTTAGGTTCCGCTTGCAAATTGAGGTGACCCGACACAGTAGGAGGTCGTAAATATTCAAGTACACCGCCAATCCATTGATACTCATAAACACCCTCTTCACACCCGCAGATCGCTAGAATACATTTTTACCACCACTAGCAGTGCCCACTATGTACATTCCAGTGATCACTTACCACGATATTACTGAGGAAATTCAGGGTTCGTCAAAGTTTTCAAGAATGAGGCATTTGAAGATTCTAATGAAAGTATCACCGAAAATAAAATGTTCTCTCAGAATTAAGTAGTTTTAAATTATATATAATCGATAGAGCTCTTGTTTTCTCCTAGAGAAAAGGTGGGAAACAAAAAATTTTAAATGATTCGCCTTCCAAATCAGACAATCCCGACCTCACCGTTTAGTAAGGAATAGATGAAATAACTTTTTGTTTTGTCTTGTGAAGTAATGTATCCAAAAGATGAACCGGAAGGTTTTTTAGCCAAGAATCGTGAAATTCTGAATATTTTAAATCTGCAACCTAAATTTAGTACTAAGTTTTCTTGAAATAGATTTTTCGCAACCTAAATTTCAGTGGGAAATTTTGAAATTCAAATTTACCAACCTAGATTTTCATATATGTTCTAGCCCCCTCCTCAGTTGACCTTCCTACAGTACTAATGTTTCCCGGAAAGTTCTAAATCTGCTTTCACAACGAACACCTGCATTCCGTTTTTTACAATCCCACACAGGTGTTGGTCATATCGCAACATAACAGTAAAAAAGTACACCGTACTTTGTCTCCTTATTTCTGTAAATTCAAAAAGAAGAAAAATGATTTCTATCTCGATTCCCACAAGAATTTTGATAGCTTTTGGTAGGTTTTAAATAAGAGTTTATATTGTCTGTTCAAAAAAGTTGGCAGTTNNNNNNNNNNNNNNNNNNNNNNNNNNNNNNNNNNNNNNNNNNNNNNNNNNNNNNNNNNNNNNNNNNNNNNNNNNNNNNNNNNNNNNNNNNNNNNNNNNNNNNNNNNNNNNNNNNNNNNNNNNNNNNNNNNNNNNNNNNNNNNNNNNNNNNNNNNNNNNNNNNNNNNNNNNNNNNNNNNNNNNNNNNNNNNNNNNNNNNNNNNNNNNNNNNNNNNNNNNNNNNNNNNNNNNNNNNNNNNNNNNNNNNNNNNNNNNNNNNNNNNNNNNNNNNNNNNNNNNNNNNNNNNNNNNNNNNNNNNNNNNNNNNNNNNNNNNNNNNNNNNNNNNNNNNNNNNNNNNNNNNNNNNNNNNNNNNNNNNNNNNNNNNNNNNNNNNNNNNNNNNNNNNNNNNNNNNNNNNNNNNNNNNNNNNNNNNNNNNNNNNNNNNNNNNNNNNNNNNNNNNNNNNNNNNNNNNNNNNNNNNNNNNNNNNNNNNNNNNNNNNNNNNNNNNNNNNNNNNNNNNNNNNNNNNNNNNNNNNNNNNNNNNNNNNNNNNNNNNNNNNNNNNNNNNNNNNNNNNNNNNNNNNNNNNNNNNNNNNNNNNNNNNNNNNNNNNNNNNNNNNNNNNNNNNNNNNNNNNNNNNNNNNNNNNNNNNNNNNNNNNNNNNNNNNNNNNNNNNNNNNNNNNNNNNNNNNNNNNNNNNNNNNNNNNNNNNNNNNNNNNNNNNNNNNNNNNNNNNNNNNNNNNNNNNCACAGCTGCCAACTTTTTTGAACAGACAATAAATCCGTTAAACACTTAATTTTACGTTTTCAAAGTTTCAGAATCATGTCTCCCTTCGTGGTTTCGACAAGAACGTAGTGCCCCAGAATCGTTGAAAGGAGAAGATATTTTGGAAGATGTTGTTGAAAATAGTGGGTCGCCACCTCTGGACACTTCACGAAGCTCTCTTGAGACCAATGAGATCGGAGAGGTTCAAGATGAAATTTCGACATCAGAGACTTCAGAAATAACAACTCCCGACCCGGTTGAAGAAAAAGAAGATGAGAAGTTGGCAAAAGAATTAAGTGAGTTTTTCCGCTCAAAACATTTTTCATTAATCGGAAATTTCAGCGGAATCGGAAGCCGAGTATGAAGAAGCATACGAAGAAGAGAAAGTTGAGGAAGAGGCTGAAGAAGCACTTAAATATAATGAAGATGCCACAAAAGAAGCTGCCTCCACGTTGAAACCTTCGGTTCAGAAGGAAATTGAAAAGTTGAAGGCGGCGAAATGTAGAGGTAGAGTCCAACTTATATTATTAAAACCGCCGGTCTGAATTTTTTATTTATCAGAGTTTTCATAGAAAAAAATTCCCCATTATTTTGCCATCCGAATTCTTCAGTTCTAACCCTTCCTTTCAGACTACTGTCATCACAATGCTACTTGCCACGTGGAGGTGATTTTCCGCGACGACCGAGTCTCGGCGGTTGTGCCATCTTGCCAGTATGCAAATTTTCAATTTGAACATTTTTTGCATTCTCATTTTTGATTTTCAGCTGTCCTCACGGCTGGGAAGGTACTCGGTGTGACCGTCACTACGTGCAGGCGTTCTACGCGCCCATCAACGGCAAATATAATGTACGTTTGAGCACGATGAGCAGCACGGCGCCACTCTTCGCAGCTCAAGTAATAATTCTAACAATATTCCGAAAAAGATCTAACTGTTCCCTTTGACTGCCCTTTTAACTCCCTATTATTGCATGATTAATTGGTTACTCTGGTTTGGTTGGATTTGCGGTGACGGTTAATTTTTAAAGTCCACTAAATCATTTGTGGTTTCTTTCTAGGAGAATGTAAGAAAACCAAAAGTTTACATGGTTCATCCTCCCAATCAGGCGGTCCCGACAGCACCGTCCAGTGAAGATTCTGATATTTCGGCAGTTTTCAGTGGTCTCTATAAGAGAATTGTGGTAAGAATCCCGAGTCTGAGGCCTAGAAATTATCAAAATTTTTGAGCAGTATTGTTCAGATTTAATTTATTTTTTCAGCAATCTTCTTCATCTGCAATTCCAGCTTTTGCATTTCTAATCATCATGCTGATCATGTTTATAACAATCGTTATCTACGCTTACAGAAGGTAATTACAACATTCTGATATAGAAGCAGTGTTTAAATTCAGAATGTCAAAACGGTCAGACGATATGACGTATACAATGAGTCATATGTGTCCTCCAGAAGCTTTCAACGTCCTAAAAACTCCAAACGGTCGTCATATTCCGATCCATCAAATGCCATCTTGTCCTCATGCATCTGGAGGCGTTTCATCAATTACAGTAACCCCTACATTACCTTCTTCTTCGTCAGGGATCGGATGCCGACCTCCAATGAGACAACAAGCCGTTCGAACCATTGACCAGGCACGAAACAACTTTTTCAGCATTCTCAGAAGTCAGGGAACCATTCCATCCAGGTAAGTGATATTCGGAAAGCTTCAAACAACTATCACCTGCATTCAGAAGTATCAACGACGACGACACACCGAAGCACTATAAATCAGTGCCGCGCGTAGAAGTGTCAGCAATCAACTATTCCGGGCATATCGATTTCTCCACGATTTCTTTTCAATCGGTACGTTTTCTATTCGATATGAAACAAATTAAGGTTTTTTGATTTTCAGACAGAATCGGAAGTATCGAAGGCTTCAACCACGTGCCCACCACCTACACACACAGTAATAAATATCGAGCAGGATACTGCAGATACGGTAATATTATGTTTTTTACCCACTAAACTTGAATTGTTCAACGTATAA

>C.ANGARIA

CAAAATCAAGTTCTTTCCCTAATTTCCAGCTCAAAAATCCATGATTTTTCAGCCAACAATATGCTCATCAATCACTTCATTCCAATAATAATTGCTGCAATTTTTTTGAATTTCCCACAAAAAACAGGTATTTCCCTTATTTTCTTTTCTTCAATAATAAATCAAAAAGCTATAGAAAACATGTGTTTTTCTTAAATGAAAACTCTGAAAATTTGTGGCGAGATAATCCAGAATAACCTTGTTTTTGCCACGTCATAAACAATTCTATCATAAAA**AGTTTGTTTGGGGTTGGTTGTAAAACCAAATTATTCTCAAGTATTTATTACAAACCTCCTCCCTCCCCTTCCGATTTTGATCAAAACTTGTATAATTTTTGGGAAAATGTATATTCCTCAATTTTTTGTCAAAGATTTTTCGGCGCCAGGTGTGTTTATGACTCATGTTAGGGCCGAGTTCCTCGTAGAAAAATTCCCTCAAAAAATCTTTTCATTGTGAAAAAT**ATGCGAATTCCTACGATTCCCGAGCGAATTTTGGGTGTTTTCGGTAGGAAATTATTAAATTCTGACTCAAATTTGGAAAAAAATTGAATTTTTGTAGAATCTTGTCTTCCTCAATGGTTTCGCGAAGAAAGATCGGCCGGTTTTGAAGCAACAAATGAAATTTTGGAGCAAAATTTGGAGGAAAATGATAAGAAGAAAATTATTGAGCAATTGAGTGAGTTTTACTTGGGCTCAAAAAGTTCCTCATTGTCAAAAAATGTTTGTTTTTCAGCTGATGATGAAAATGAATATGAAAATCCAAGTGTGATAGATGGTTTTGAAAGTTCGCAAGAAAGTCAATTTATTCAAGAAGAAGAAGAAGAAGAATCGCAAGAAGTGCAATATAATTTGGCAGCAACAAAATCGCCGACAACTTCGATGAGTCCAGAAATGGAACGAAAAATGGAACAATTGATGATTGAAAAATGCACAAATCATTGCGAGAATAATGCAACATGTAGTTTAGAAATGTATTATAAATCTGCTGAAGAAGGCACTATTATTATTCCAATTTGCCAGTAAGTTAGATTCTGAGAGATGGCCGAAAAATGTATTGGTATTTTGAAGGGAGAATTGAAAATTGAGAATTTTGGACCAAAAAAGGAAATACGGAGCTTAGAGAAAATAAAATATAAGCTTCAAATTTGAAAACACAAATCTCTTTTTTTTACAGTTGTGAGGCTGGTTGGGAAGGTCAAAAATGCGAACGTCAATATGTTCAAGAATTCTACGCGGCAATTTCTGGAAATGGACAAAATGTACGTTTTGTTATGTTGACTGGCAGATTAATCTCATCACAAACCCAAAAAAATGTAATTTTTTGTTGAAAACACCTCCTAATAATCATGACTAACAAAAATAATATGAAAAAATAAAGAAAGTGTGGAATTGTTTCTCGAAATTAAACCAAACTGCATGATTTTTTGAATTTCTAACATTTTCTGCATGTGTGCTCTAATTTCGAATTATATTTCGAACAAAAATTTTGAAGGTTTCTTCCACGCACAACAGAGTTGCCAAAACTCAAAATGTCAAAAAACCGGCAACTGAAACTACGAATTTTTTTGGAGATTTTTCATTTTTGGTATGTTAGGAATAAAAATATATTGAGAATAAGAACATTTTTTAAATTCTAAGATTTTTAGCTTGAAAAAAACGTTGAAAATATTTGTTTGCAGCAAAATCCTTCATCTGCAGTTCCAGCAGTCACTTTTCTTATTCTCATGTTGATTATGTTTGTATCGATTGTTTTATATGCGTATAAAAGGTGGGTTGATTTTAAAATTTTTACCAGAATTGATTTATTTTTAAAATTTCTATAGTTTTAAAAGAAAATCCCAAATTGTTTTGTGTTGTGCTAAAAATCCACGTGGCACAAAACGTTTTTCCAATTTTTTCCCATTTTTTTCAGAATGTCTCGACGAATCGATGAAACAACATACACAATGAGTCATATGTGTCCACCAGAAGCATTCACAGTTCTCAAAACTCCGTGTGGTCGGAAAATATCAGTTCATGAATCGCTTTTCTCATCATCAGCTTCATCTTCTGGCGCATCAGGATTAGGACAATCAAATTCAGGATTTGGCACAAGAAATGCTCGAAGATTGAATTCGAATTCTGGTCCAATTATGACATCAACTCCAGCTGTTCATTTACCTCAAAGAACACCGGCTGTTCGAATTTTGGAAAAACCAAGCATTTTTCAAATTCTTCGAAATCAAGGATCGATTCCATCGCGAAGTATCAATGAAAATGACACGCCGAAATATTATAAATCAGTTCCACGTGTCGAAGTATCAGCAATTAATTATTCAGGACATATTGATTTTTCGAATATTCTTGAGACACCGGTGAGTGATAGATTTGAAATGGAGTGTAAAGTTGGAGTATTGTTTGAAAAATCTTGAAATGCTCGGGATCTCTGAAAGGAATCTGAAAAATTTAACAATTCTGAATGTTTGTTTGAATTCACGAAATTACTCATGAACCTGCGGAGTTTCGTTGTGAATACTACGACCCTCCGAAGTTACCCCATCATGTTATATTTTTCAGGAAGAAAGTCGACCGCATCGAACATCTCCGCCTCCACATATAGTTATTCAGATGGATGAAGAGAGTATTTTTTTCTAAAAATTTTCAATCACAAAAAACCTTTTTTTTTCCAGAGGCACCCGATCAACTCTCGATCCAACCACTTCACCAATCGCCAGACTGTGAtctaatttttcctccagaaaaatttaaaattctagtcaaaatctagtctctctctttccctcgggtttcttgttcaatttcacttttctcctcctctcaatcactttttcctgcaaaaaattaaacaacgggtgcccaaattttccccgctccgatcgtaaaattcctgtgaattatatatagtttttatatgttttctttctttattttttatgtatgtatttttgtaaaatagctgctatgttacgtatatgaataacttaccttaacttcaacagatttaaaaattaagacaaggttgaagtcgttaaatatttatatttaaatatattcaaagtaacatctcgcagagattaggtcaaattatcacaggaaaatgtacaaaaatgtgggcggagatcaata

>C.CASTELLI_incomplete

CTATTTTTTTTGCTGAAAATCCCTGAATTTTTCCAGCCAACAATATGCTCATCAATCACTTCATTCCAATAATAATTGCTGCAATTTTTCTGAATTTCCCACTAAAAACAGGTATATTTTCTTGCTCAAAAATAAATCAAAAAGCTATAGAAAACATGTGTTTAAAAACATGAAAAAAAAACTTTTTTCTAAAACAATTCTGGTAAAAAATTCACAGTAACCTTTTTTGCCACGTCATAAACAATTTGTTGAATATATCTATCTATCATAAAAAGTTTGTTTGGTTGTAAAACCAAATTATTCTCAAGTATTTATTACTAACCTCCCTTCCCCGCGTTTTTGATCAAAACTTGTATAATTTTTGGAAAAATGTATATTCCTCAATTTTTTGTCAAAGATTTTTCTGCGCCAGGTGTGTTTATGACTCATTTTTGTTTTACTCGCAGAAAAAATCCCTTTTGTTTTTTTTCTAAAATCTGAATTTTTCTTACAAAAAAACTATGCGAATTCCTACGTTTCCCGAGCGAATTTTGGGTGTTTTTGGTGGGAAAATTCAAAAATTTTCCAAAGAAAAAAGTTATTTTTGTAGAATCTTGTCTTCCTCAATGGTTTCGCGAAGAAAGATCAGCGGGTTTTGAAGAGACAAACGAAGTTTCGGAGAAATATGAGCAAAATGATGATAAAAAAATTATTGAGCAATTGAGTGAGTTTTTTCATCACGAAATTTCCCTTGTAAAATCTTTTAAAATTTTCAGCTGAAAATGGCGATGAAGGTGAATATGTAGAAGATGAGTTTGAGAGTTCGCAAGAAATTCAAGCAGAAGAAGAAGAAGAAGAATCGCAAGAAGTGAAATATAATGAGGCAGCAACAAAATCGCCGACAACTTCGTTGAGTCCAGAAATGGAACGAAAAATGGAACAATTGATGATTGAACAATGCACAAATCGTTGCGAGAATAATGCAACATGTAGTTTAGAATTGATTTTTAAATCTGCTGAGGAAGGAGGACCTACTGTTCAACCCATTTGTCAGTGAGTTTGATTTTGGACTTGAAAATGACAATCTATAAAATTTTTAGTGAACTTTCTAAATCTAACAAGGGATTTTCATGAGGGTGATAACGGGTTTTGAAAAACAGAAATGGCCATCTTGATGGGCTGATCACGATGCCGTAAAAATAAGATGATGCTGAGCTCCAGTCCGAGCCGCACAAACGAGCTCCGAAGAAAATTTTAATACAACATTTTGTGAAATGTTTGTATTTGTAAATTCATGTTTCTGAAAATTAAATTAAAATCTCACGTGAAAATTTTTTGACTCATTTTTTTGGTGTCAATTTTTCGATGGTACCAAAATCGACTCCAACATTTAAAGTTTTTCAAAACCTGTTATAACC

>C.VIRILIS_incomplete

GGCCGGTTCCGCACAAGCCTGCTCGAGACCGTTCCGCAAAACCGCGAGACATGATTCATTTAAGATCATATCTCGGTATCCTCAAAAGGAAAAATATTATTTGCTTTATTCAACTTCTTCTGAGGGGTGAAAAGATTCTATTGTATTTTTTCTGTCCACAGGTGTTTTCGTAGGGCTTTCATGAAACAAAAGATTGTATGCATCTAGTTTTTTGGGAAATATTTTGATAGTATTCTCTAAAAAGTACGTTTTCAATATTTTTACGTCTTACTTTAAAAAAATTGAGAATTTAAGAAGACTATGTTTTCCTTTGGACGGAGAAAGTATATTCCTACAATTCCAGAAAGATTATTGGGCGTTTTAGGTAATTTTCGACTGAAATTTAAAAAAAAAGCTCGGGTTTGGGCTGAAAACAAATATATTTCTCTGTTAATTTGAAAGTATTTTTCCGAGTGTTTTTTGTACAAAAAAATCCTAAAACCTAGAAATTTTCAGAATCATGTCTACCCTCTTGGTTTCGACAAGAACGGTCAGCCGAACCGCTGGAAAGCATCGGCGGAGGTGCAACGATTCAAGGAAAAATTGAAGAGACGAATGAGATTAGGGAGATTGATGAAAATGAGATTGGAAAGGATGAAGCTGAATTACAAACGACAACAAATTTGAGTATTTTTGATGAAAAAACGACCGAAATATTAGGAGTTGTGATGAAGAAAGAGAAGGAGGAAAATGAGATTATTAGGGAGAAGGTGGAGGAGTTTGGTTGGTTTTTAAAAAATTCTTACATCTTCTTGAAATTGTTAGAAAACGAGTAAAGTAATATTTTCAACTGTTTTTTTTTAATTTTAGAAAATATTGATTTATTTCAGTGAGTGCAGAAGCCGAATATGCGTATGAATATGTGGATGAAGAGGCTGAAAAAGAGGTAGAAGAAGAGTTGAAATATAATGAGAATGCAACAATGGCCACATCGACACTGAAACCGGAACTGGAGCTGGAAATGGAGAAGGTTAAGGAGCAGAAGTGTAAAAGTGGGTTTTATGAGGGTATTGTCAACTCAATTATTCTTCATCATTGCGTTCGGAAGGCTGCTCAACCCCACAGCCGCTCCGCGAGCACGCGGCGCGGCTGCCGACACTCAACACACTGGAAAATTTGAAAAATTCATTTTCCCGCCAATCTTTCGGTGTGTTGAGTGGGAGGTCACCGCGTCGCGTGCGTGCGGAGCGGCTGTGAGGTTGAGCAGCCTTCCGAACGCAATGATCAAGAATAATTGAGTTGACAATACTAGAAAAAAAATCAAATTTCGATTGAAGCTGGAAATCGGGGCTTCTGGAAATTTTCCAGTAGTTCAGACAATCAGACAATGCTCTGAAAATTCATGAACAACAACGAAATTTTTTTGACGAAATTCTGCTTCAACTTCAAATTTTCTGAAAATGTTAAAATGTTTCTCATAAATATTTATTTATAATCAAAAATTATTTTTATTTATAGATTACTGCCATCACAACGCCACCTGTCACGTTGAGCTCAATTTCAATGAAGATGGTCATGTGTCAGCGATTGTACCGTCTTGCCAGTGAATTTTTTTTTCAATATAAAAATTTTTATGAAGGCTGCAATTTTGAATTGCATTTATTGGGGGAAAACATTTAGAAAAACGTTAAATTATCTTAAATTCTGGTTCTGAATTACCTTTCACAAGTGAATCCTTGTGATTAAAAAATAATAAACATTTTCGGAATTTTGTATTTATTCAAGAGTTTGAAGTATTCATCCAAGCGTTTAAAAAATTAAGCATATTTAATTTTTTTTTTCGCCACATTATTCCACTCCCTCTTGAAGTTTGTATTAAACTAATGAAAAATAATTAAAATTTTTGTTTTTTTCAGCTGTCCACCCGGCTGGGAAGGAACCCGATGCGATCGTCACTATTTTCAGGCGCTTTATGCACCAGTGTCGGGAAGATATAACGTACGTTTGTTGTTGTCGACGAGTGCATTACCCATACAAGTTTGTGTTTAATTAATATATACTACATGTAACTTGGTGATGAGGGATTATTTTTGTGGAAAAAATCTGGAAAAAATGTTTTAAAAATTTGTTTTTTATGAATTTTTATGGTTAAAATGGTTAAACATTTTGAGTTTTTATAAGAAATAACTGAAATTTCAATTTTTTCCAGATTTTTTCCCTATAGTAATCCAAATAGTATTAATTGGAATGGGTTTTGGTTTTAAAAGCACGCTCCTCTTCCCCAGAATGGATTTAAACTTATAATTTTTTAAACAATTTTTCCCAAATTATTGCTTTTTCCCCTATGGATTTGTATCTACTAACAAGATAGAATACAAATAATGGATTAGAATATTACTTTAGAAACAAACCGATTCCAAACAAGCTCTACTGACAAAAATCGGACCAATATATACCAGGAATGCATTTATGACTAAAAATAATATTTTAAGTACGTATGGGCACGGTAATGGCCATAAAAACTTGCGCGTTTGGAATTCGAAATGAACGAACAGTAATTTGCATGAAAGTTTTACTGAAAATCAGGCTTGGTCGGAAAAAACTTTTCGGAAAATCGGAAACGAAAATTTTTTT

>C.SP.1

GTAATCGCTCCCTGTTTGAAGTTGCTGTTACTGCCAAATCCTATGTGGAACGCGCCGCCGTCGGCGTCGAAACTTGAGGAGCTGCCGGGGTTCACCCGACGACGACAGACGACTTCTGCCATGATCTTCGACTGCAGGTCCGTGCTCGTCATGTGCCTCGTCGTCTCGTTCTTCCAACCGGCAGGTGAAGTTTGTTTTGAAATACTATCGCTATCGCCGGTTTTCAAGGGTTTCAAAGGCACTTTACCGGTAGAAGTCTTTCGAAGTTGTTGTGCACAAGTAAAAAACGCATAAAATTTAACTGCGGCAGTTGTGAACCAATAACTATTAGATTTAATGATACCGGTAGTTCCCTTAATCCCTATTTGCATCCTTCTAGGACAAGAATCTTTCAGAAGTTTAGCACTAGCTGGTTCTACCAAACATATTTTTAATTACCTTAACTTGAGAAAGGTTCCAAGCCGGTCATCTGGATTGTTGATTTTATTTGCTTTTTTACTTGTAGGAGCCAAGAAACTTCGAAAAAAGCCCCGAGTCTTTTCAAAACGATAGGGTATCTTGGGGGTTTTTTTTACTTCCATGTCTCACTCGTTTTTGAAGTCACGCCCGAAAAAGGTCAGAATTCTGATTCACTTCCAAGATTTAATTAACCATTTTCATTCGCTTGGTGCGAAAGAATTTCTGCCCAGTACTCGACAGTTGTCTGGTTTCCCAGTGTCTCGCCCCATTCGTCCGTTTCAGAGGATGATGGATATCCAAACATCAACCAGCGGCTGCTAATGAACCCGTGCGAAGTCAATTTTTTGTCGTTGGAAGCGTAGGATACTTTTATTGTTTGAAATAATTTGCTTCTATTTGCAAGCAATGTTCGGGAAGTCGTGCATAATGAAGAATTTATAAGCGTGACCAACTTCAAGGTTTTCAATATTCTTGAATAGGGTCCTTCCTTGAGATTCGCGGGGTATATTTTTGCAAGAAGGACGGCAGATCCACTGGTAATCTTAAAAAGTTCCAATATTTAGCATATTCACGTATTCTCGAACAATGTTTACAGGAACACGAATGCTCGGGTCTGCCGGTAGGTATTAAATTTGGCAATATTTAAGAAAAATGGATAGCAAGTGGTCACCAGACGTCTGCACGGCGTTCTGCGCATGTGATGAATTTATTTGAAAAATCGTGCTCAAACTGCAAACGGGTACGGTTGCTGATCTACCAGAAAATTTCGATTTTGATCCAACTGCAAAATAAGATttcttggtcaaaatgatagtttttgtcactaatattgttgctaattttttaaataaaaggaaaaacgtaatataatgttaagaaaaatacaaattaaagcTTAAAATTAGTAAAACTCGCGCTCATAATTCTTCCGCAATTCTGCAACCTAATGATCTACCATGCCCGCGCAAACCAGTTTTTCAAATAAATTCGCTATGCCGCAGAACGCCGTGTCTGGAAAGGGATAGGTTTCATACCAATTTTATTTCTATCCTTTTTGTTTGAAAAAGAGCATAGCCTTATGGAAATTGATAAGGAACGTTGTTCAGTTTGCCAGGGGCAGGTTCGCATTAATCCGAAATTTCGTCACAAGATGGGGGCAAGAAGATTTTCGATTGTCGCTGCGGCACAAACGGGGATCGGACCCGGAAAAGGGTTGTAATTGCTTCTGTGCCAGCGCTGGCATTGCAGCGGCGCGCAAGGTTTACACATGTTGGCGGTTTATTTGAATATCCAAATTGGATGCTGACAAAGGTCGGCTGGCCGGAAAGACAACGCGTGGCCGGGAGAGACTTTGCGTCGTCCATCGACGGGTCGCGATTTTGGGCCCGTTTTATGGAAAGGATCAAGTGGGGCCAGTGCGGGGAACACTCCTGTCAGTCGATGTACATTCCAGTGCTCCACTGGATCGCCGACGTCCACAAGACACGTAGGTTATGCTGCGGATGTAAGCGTTTTAGGCTTTGCACATCTGGCTGTAAACATCAAACCTAGGTGTGCAGCTGGACAGATGCGCGCTTTTTTCTTATTTTTCATTTCTATTCACCACGTTTAAACCGCGCGCGCATAAGACAAAGAAGGCGTTTTTCGGGAAGACACGAGGGGGGAGGGGGCGAAGGTGCGACGCGTACGGGTTTGGGAAGAAAACTCTGGAACTGAGCACGCGACACTGATGGTGACTGGTCTTAAACGCGCACCCACCGTTCCTGAACGTATTTTGGGCTATTTCGGTTTTGCCTATTCTGATTCGACGGGTTCAGTGGTCGATTTCAGAGACATGCCTACCTTCATGGTTTCGCCAGGAAAGGGCCAGCGAACCCGAAGCGCTCGACGGCGACGCGGTGAGCGACTCTTCCAAGGAGATGCTGACGAATTCGATTATCGACGATGCTACTGATGGGTTTGCAAGTTTTTGGTTTTGCGTTGTATCCGCTGTCTGCAGGGCTCCTGTAAATGCAAGCGAACCGCCTTCGAATTCGGTTGAGTCGGACGTCGCAAAAGTCAACTCTGGAAGCTACGAAGAGGAAGAAGTGGAGGACATCCGCTACTACGTGCGCACGTTGCCGACGACGACGTTGTCGCCGGAGATGCAAGCAGAGCGCGACAGGATTTGCCGTCGTAAGCGTCGTCAGTTGTCAGTACGTAAACTTTCGTTGTTCGTTTGCAGATTACTGCCAGAACAATGGTCTCTGCGACATTCAGTTTCATCCGCGCATTTGGGGATACGAACCAGTGCCGTTTTGCAAGTATGTAACTTCCTAAAATGCATTTATAGGATCTATGAAGTATAGGTGCCCCGAGAATTGGCACGGACGACAGTGCGAGGACCCTTACTTTCGCAATACTTTTGAAGCTGTCAAAAGTCCCTATTCGGTAAGGTTTTTTGAATTCATTGCAATGCAGTGATCAGAAATCCACGAATATGCCAAAAACGTTCAGCTGCATTAATTTTCATGTTTTGTAATTCTTGGCTAGTGCCTTTGAGGCGGAAAATTCTTTATTATCTAAATACTTCCAGAAAGAGGCCGTTTTCCCACCATTTTTAACGCCCATTTTAAACAACATCAATTTGCAACAAAAAAATTCTGCATAAAAGTGGTCCTTGCGGGTAAAACAACCAACAAAAAACTTCTTCCTACATTTTTAAAGTATTATTTTACCGAAAAAATGTAATACCTGGCAACGAGCAGCTAGAACGTGTTCGAACACAATGTGTGTTAGTTTATGGGGCTTCTGACGATTTTCCTATAAGATTAACATCTAAATCACGATCGTGTTATAGTCCATTCGTGAAATTGCGGTCGTCGAAATTTAAAGGTTCGAATCGCGGGTCCCGACGCGATGGCTTGTTTTTTGCCTAGGTACTGTAGGTTTTAAAAATGCGACCCTTTAAATTCTGGCGACCGCGATTTCGCGAATGGACTATCTTATAGGAAAATCGTCAGAAGCAAAACATGAAGTTTGAATTTTTGGCGTATTAATGGATGTTTTGCCTGTTTTTTGTTTACTATCGTGTGTAGGTCAAATCCTCGGCGCTTCCTGCATTTGCGATGCTTTTCTTCATGCTGGCCATGTTCGTCACACTCGTCGTGTACGCATACAAAAGGTGGGCCAGCGGCGAAGGGAGAAAACATTCGAATTTCGCGCCTTTCAGGATGGCGTCACGGCCCGAAGACTCGTCGACCTACGCAATGAGTCACACGTGCCCATCGGACGTGTTCAACGCGTGGAAGGTTTTCGAAAAAGTTGGATTTAACCCGAACGTGCATTGAGCAGTCGCCGTCGCTCAGAACAATGGCGATCAGTTCTCGGCGGCCGGCTTCTCGCCAGCCATCGCGGGCCAACGCGAGATTCTTCGCGCGACTCGCCGGCAGCATGCCTTCCTCCGTCAACAGGTCCGCCCTTTGTGGTTTTCTCGGCGTGTACAGGGGTGTTGTGTCGTTGGAGAAGGTAGAAGTAGATGAGTTTATAGACCTAAACTCGGTGAAGTTTAATGATCACTAGTGATGTAGCTATTTATGTTCTTAAGTAACTGGGACCTTGCCAGACACGGGATGATTATGATAAGAGAGTGGAGGTTTATAGAGCTAGAGTTTGTCTGTGGCAAGTGACGTCACCTTCACTAGGTTCGCATAGTCTGTTTCCCAGGGTCACCGACATAGATTCGTAGGCTACGGTAAGCATAGATCACAAATATTTTTATGATCTGCACTTGTAGCCTTTTGTAATACCAAACTAGGAAAATTGAATTATTCCACATAAAAGATTTAGGTCAGGAACGCTGATATAGTCGATTCCAACCAGGAGTGGTAGCTTCCAAAGTTGGCAGAGAGCCAATTTAGCTGGGTGTCTTGAATTTGCACGTCTTTTCGAGCTTTGGAGATCGTGCAGGTCGCTACGCTTGATTTTTGGAATCCACTAGTTTCAAAAATTTTAATTTTTAGCATAAACCTGTGCATTTATTGACCACTGCTAGAACCAAGGGGCACATTTTCTTTCATCGAACTACCGAAGTCGAAGTCAAATCCTTAAACAAAAATTATCGTTGCTAATAACATACATTTTAGAGTTAATCCAGGAAAATTGTTTTTTTAATGGGCTAAAGTAGTGGAACTCACTCGCATAGATAATACGCCTGCACAAATGATGGGCTGGCACCCATAGTCAGTCATTATGGTCATTGTTTCAAATAGGATTTAAATGAATCAAAACCAAAATTAAGAAAAGGTACGAAATTTTCGGAATTTTGATTTTGATTTATTTAAAACTGATTGAATCAGAGATTAATTGAGGGCCCTCGGCAACCTCTCTCTCTCTGCACATCTAACTTGTAAGTTTCCCGTGAAGAACAAAAATTTAACTGGTTTTGTGTCATGTGCGGGACGTCGTGCAGTTTCAGAGACGCGATGCCGCAGCAGTACGGGAAAATGCCGCGCGTTGAAATATCGGCGATCGGACAAACAGGGCACATCGACCTGTCGGCCCTCTCCTACAGCTTTGTTCTCTCACCTTCGTCGCCTTTTGCCCAAAACTAATGTGTTCAGAAAGAGCAGCGCAAAGACTTGACGCCACCCACTCGGATGGTCGCCATCGAAATGGAGGCGCTGGAGTCGTCGAGGGAACGACGGTTCGTCTCTGTGAGGTCCGAGACGACGTCGGAGTGTCCGACGCGCGCTTCGTCGGCTCCGAATTTGGGTTGCAAGTCGTTTTCATAGTCTTCTTAACTTTTTCCGTGTCGCTGTTTCTCGCTTTCGTAATGTACAAATAACTAACTTTCACAGTTTCGAAAGTGCGTATTTCGAAGCTATCGAGTAGGTTTCGCAATGGCAAACAAAAGTTTCGGCGTTGCTGGGAAAACATCTCGTGGCGTCTTTTGCGCAAATACTAACGAATTTTAACACAGAAGTTATTGCATTGTTCTTAATTAAATGTGCTCCTATGCACTTTATGTGAAGAAATGCGACGATTGGGCTCCGTTGCAGATTTAGGGTCCAAGGTTACATGTTACTCGCCTAGCTTTCAGGCTTACAATAAGGACCCTTATTTGTTCGGCGAACTCTTAATCTGCAACAGAAGCGACGTCTCATTTTTGTTGGATTGTTGCATAGGAGGTATTGCATGTTGTGTATGTTTTCGTATCTTCATTGTGATACTCGACTGTTGAATGCTAATTCCCCCAAAACGGGTCCTTTGCCATTTGTGATGTTGTAGTCCTTTTTTTTTACCGTCAGCAGGTTTCTATAAATGGTTTTTAATTTTTTATCCACCAAAACTATGGCGAACGAACAATTCAGCTTAAAAAATAAGCTCAGAAGAGAAACCAGGAAAAGTCTCCATTTCTTACAATGTATAGTCGGTTCCCCAGCAGACCCGGCAAACTCTGCAGGATACAGTAACCGCAGATCAAATTAA

>O.TIPULAE

ATGCCTGACGGCTTGGAGGAGCCGTCACCACCGCTGGCATCAAGAGAAGAGGACCTCCCCGGCTATTGTTCCCGTTTCGGCACTAACAGCAGCCGTCGCCTTCTACAGCTGCCAGCTCGTCTGCTCGTCGTCGCCTTTTTCCTCCTTCTAGTCATAACTCCAGCAGGTTTGTCAGCCGACTCTTCTTTGCTTAGGAAACAGTCAAATAAAGGCGATAATTAGAGGCTGGCGGGTGGCACGAAGCTCATAGGCTCATTGTAATATGGCCACTTGGCGAAACGGATGTGGTCAAACTATTGAGAGGAGGATTTCTAGGGGTATGACATCTGTTCAATGGCCACAGTTAAACAAAAAAGCTAGGCCCTCTTCCAAGCCAAAGCAAGACAAGCGTTCGGTTATGGCCTGAAAATCGAATCATATCGCTGTCCTCTTGTCCACCCGAGCACCCAAATTTGAATGCGCAAACAGACTCGAGGTCGTTTATTCGCTCCCACTGCCACTGTGCTGGTTAGCCTGGAGTTTCGGCCCCTTTGTCACCTTTCTCGACCCGAATTTATTACAGAGGAGCTCTATATTGCGCCCTCTTACAATGTATCTACCGCTGGTTTTACAGCTCTCCGAGAACTATTTGGCAGGTAAAATAGTCTCCGTACCCTGAAACCCGCCGATTTTTGTTGAAAAACGATAAAAAATAGCTTGGAAATAGTATGAGGCACATGTTTAATCAACAATTTCCTAATTTTTTTTTAATCGAAAGAAGAGCAGATATCTCCCTTTAGAACGATACCCGAGGCCGGAGCCATTTTTCGCTACCTGGCGGCCACCTGCATGTCCTTTTTGCGCTCTACTACCACGGGAATTTTCGCCGTGGCACATCCACATTCCGTTTTCTACTGTACTACCCCCAATTCGCGACTAATTTGGCCATGAGCCCTCGAAGAAATGCTACAATTCCCGAGAGAGTGCTCTTTTTCCTAGGTAAAAGGCTTGGCTAGTTTCTGCTCGAGGGAAAATGCTCTTCAAAAGTCTTTTTTGTGATTCACCAGCTGAAAGTACTGCCTTTTCTCAAAAGCACACACTATAAACAGAGGCTGAAACATAGTTTCGACAAGCCAAAACACCAGAAGATAGAACGATTTTCAGAAGGCTGCTTGCCGTCCTGGTTCAAAAAGGAGAGGAGTTCTCCGGCGGCACTGGAAGGTGGGATAGTGTGCCTGGGTATCTGTCGAATGAGTGTCCGGGTCCAAACGTCTTATATGCAGTGGCAAATGTCAGAAAATGAGGTATAGGATCGTGTTAGAGCCGGTGTAGTCACCAAAATCGGCAAGACATATGTCTGACTGACTCTAACAACGTTTTCCATCAGTATCTTCTAAAAATCTGGTGTGAATGTATTCACCGCCAAAAACCTTGATACAATCTGACACCTTTTTCCCGCATTTTACCCTAGAAAATTAAGAAATGCGACTGATAAATTTGCACTCATTTCATAAGAGAAAGCAAAATAGTATGGCTGTAAACCTGCTAGTTGGAGAATAAATCTGTCTGTGCTGAAAAATAAGGGGTTTGACTGTAGAAGCCCGTAAAAGTGGGATGAAGACCTTTTCCAGTTCATGTTCAGCCTAAAACCGCTACATTTCACTGACTGAGCGCCTCTGGCAAGACTTTTTTTCAGACATTTTTCCCTGTAAAATTGGCAAATCACCTGTTGGGAATACAAAAATTCGATCAATTTTGACTCTTTATGACACCCTCTTTCCGCCCCCTTGACTCCATAAATTTTGTTCTGTTTCAGGCAGCAGCATAGTCCGGACGAGCTCCCTAAACGAACTCTTCACCACAGGACTGATAAAAGGAACAAAATACGAGGCTCCGGAGCTCGTCGACGAGCAAGGTTTCCCTTTTTTAGTACTTTTTGACATCAGAAGTTTGCAGTAGAATACGTCGAAGAGAGCGAAGAAGAGTCCGAGTCCGAGCTTGCGGGCCCATACGGCACCGCCAGTCCCATAGCCTACGATCCGCTGATAGAGGAATACCGCCGCGCGTACATTGAGCGCGAATGCCAGGGTTGGTGCTTCGCCGGCGGCAACTGTACCGTCGAGGTCCATCCGAAGACCTATGCCTACGTGACAAAGGAATGCATGTGGGTTTTCGCCTGTTTTTTGGCCAGCAAAAATAAAAAAGTACCGTAGCAGGGATCAACTGGATCCAAAAGATTTGCGCGACAGGCTCTCTTGCACACTCGATTTTAGGCTTAACATGAGGTTCCCCCTTGTTGGTTTACTCTGTCGATCCCTGCCATGGCAGAGATTAGTTGACTACCGTACTGTAGAAACTCAATTAGAGGCAGATAGGAGAGATAGTTAGTCATTGGGCCAGGTGCGCCGAGAAGGTTGAAGGTGAGTCACTGTGCACATCTCTTGGCCAACGGGAGTTGTCTTATTGCGCAAGAGAGAGAGAGTACACGTTTTTCGTCAATGTTTGCCATTCGCATCGAATGAAGAAGTTGCAGAAAACATGAAAAAGAAGATTAGGAGCACCTTAAGTGACCTAACATCTGTCTAGCTGTCTAGATTGTGGCTCAGTTTTTGAGAGACGTAGTGGAGCAAATAGGACCCGAAATCTGGCACAAGGGGAACGAATCAGAAGAGCCAGGGATTGAGAGGATCAGCGTACTAGAACAGTCTGAAAGATCTACAGTGAGAGGCATCGGCCCCTATGTGCAGCCCAAAAATGTCAAAGAATCGCGCAAATCGCTACAAAAGCAAAAGGACTCTCTTTGGAGTAAGAAAAAGAACTGTGCAAAAGTTTTTGATTATAGGGGCTCAACTGACCCCTGGCTGCGGGCCGCAAAATCCTTCAAATCTTGGCCCAGATTGTTGGTGGGCTGTTTTCAAAGCCACAGCTATATAAAAAATAAACTTCCTGTCAGCTTTGCTACAAAAAAATCATAAAATCATGCTTTCAGATGTCCTGCAGGCTACACGGGAAAACGCTGCGAGGAGCACTACATCAAGACGCTCTACAGCAGTGTCCAAGGCGACGTCGAGAGGTCCGTTTCTAGATAAATTTTTTGGTTTTTTGACCCTTTTTGCACCTAAATAACCTGCCATTTGCACCCACTAACCAGCACGAGAAATCTCGTCTAAAAAGAGCATGCTTTAGCCCAACGACAGCGGCGGCGGATAGCTACGAGCTGAGCATTTCTCTCGCTTCAGGAGGCCGGGCCAGTCGCTTCCAGATGCAGCTAGTCATGTAGGGCTTTTGCCCTCTTTTTCCATGTTTTTCCAACAAAGCACATTTTTGACCAAAAAATGAGTCAAATTTTGTAGATCCGCAGTCCCTGCATTAGCCTTCTTCTTCATAATTGTCATCATAGCCGTCGGGCTGGCCACCTACAGCTACAGAAAGTGAGACCTTGATTCTTTTGATCTATTTTTATCGGTTTCAGATTGACAAGACGAAAAGTCGTGGCGGACTCGACGTGGCGGAGCAGTAGCTCCGCGAGGACGGCCACAGCGCCAGCGACGGAATCGATCAACTCACCGGATTACTATCTCCGAAGGACTGGGGGCGTCTACAATACGAGGTCGCCTTACTCGCCAAGAACGTGGGTCTTTTCTGCCTACTTTTTGTGCGGATTATCTCTTCCAAAACTGGTCAAATTGGAGCAGAAAAAGAGCTTAAGTTCTTCGTCGCTTAAACTACAAATTAACTTTTATTGGAGAGGAACCCGAGAGCATCATTGTAACTCTGAAATCCCGACTTTTATAGAAACCGTAAAAGAAAAATAACCAGATTTTTTTGACAAAACCTGGCCACAGCAGACTTGTCTCATCCAGAAATGAGCAGGCTCGAGCGGGCGGTGAAATTTTCTAGGCTGCGTTTCGAAGATTTGTATGAGAAAAGTTGGTGCGCTTTCGAAAAATATTTTTTTTCTGACCAGACTAACCTCTTCCAGATCTCCACCCCCAGGCTTCGAGGTGAACGGAAACGTGCTGTCGCCGCTACTCAAGTTCAATGGTCAGACGACACTACCGCAGACGACACTCAGCGCCCATAGTAGCAGTTTTCCGCCGCAGCCGTCGAGGAAAAATGAGTCGAGAAGGGAAGACGAGCCCTCAAAATG

**Cis-regulatory modifications:**

>Cel:Can-lin-3(mf91&mf92)

ATGCGGAAAATGCTACTTTTTTGCATCCTTCTACTCTTTATGCCTCAATTTACAGGTAATTTTTCATTTAAAAAAAATGATGTTCTACTTGGAATTGAACGTCACACCTCTTAAAACATATTGAACGGATCGAATGATATTTGACACTACAACCACATGTGTGTTCGTGTTCTACACATTTTTTGTCTAGTGCACTAAACATTGTCATATGGAATAAAAATCGCCACAAACGACTATGAGAAAAGATTGGTTCAGGAGCAGGGAATTTTTTTTCGAAAACGAAAATGAAAAATTTAGTTTGAAACGATATTTTGACCGAAATCGCACTATTTATGTTCACAATTTCCAGTGTGATTCTTTGGAAAGTTGTAAAATTTAAACATTTATAATTTTTCAGTATATCAGCATTTTTCGCTTAATTTTTTTTCCAAAAAATCGAAAAATAAATTTTCCACCAAATACACGAAAAATCTACAGCCCTGTTCAGGCATTTTCTCGATTTTTGCTCTAAAAATACGGTACTGGGTCTCGACACGAAAAGTTTTTATTAAATGCATGTGCGCCTTTAAAGAGTACTGTAATTTCAAACTCCCGTTTTTGCCGAATTTTTAAAACTGGTTTCCGATAAAAAATTGTCTGTTTATTCAAAAACAACTATAAAAGCACACCAATTTTAACAAATCGTAAGAAAAACTTTAAAAAATTGATTAAATTTCCGCAGCAACGAAATTTTGTAATTACAGTACTCTTCAAAGGCGCTCATCTATTTACATTAAATAAATATTGTCGTGTCGAGACCACGGACCGTATTTTTGGGGCGAAAATTGCAAAATTTTGCGTCTGGCGAATAGCCGTATTTTGTGATAATTTCCGTTTACCCGGTTGCAAATTGAAGAGGTCATACAGCAATGCACAGTAGGTGATCGTAAATATTCAAGTACCCGCCAACACCGCCAAAATGTTGTATGCACCCCCTCATAAACACCCTTGACAATACTACCATCGGTATTTATTTCACCTATATTGGCAGCAATCTACGAGTTTACCACCAACTATGTACATACCAGTTATTACATATTCGAATTTAAATGGTCCCTTGAATGGTTTGTCATTAATTATTGTAGATTTTTAAAGAAATCAGGCAATTTGATAAATTGACAAAAAAATGTAGATGTCGAGAACAATAATCACAATACAAATTCAAAAAAAGACTAACAACCAATCTACAGTAGTCTAGAAACAAAGAAATAAAATCGTACTTTTTTTATTCTTCAAGTTTCTTT**CCGTGGATCCTTGAGCTTCTGTACTTTCAAAATTCTAGAACTTCCCGTCTCTCCCTATTCAATG*ATTTTTTGTCAAAGATTTTTCGGCGCCAGGTGTGTTTATGACTCATGTTAGGGCCGAG*TTCTTACCAATGTCTCAGGCATTTTTGGAAAAGTAATATTAAGAAAATTATACATATTTTCTTGAATACGAAAAATTTAA**ATGTTCGGTAAATCGATTCCTGAACGACTTCTAGTCGCATTTG

>Cel:Can-lin-3_2(mf95)

ATGCGGAAAATGCTACTTTTTTGCATCCTTCTACTCTTTATGCCTCAATTTACAGGTAATTTTTCATTTAAAAAAAATGATGTTCTACTTGGAATTGAACGTCACACCTCTTAAAACATATTGAACGGATCGAATGATATTTGACACTACAACCACATGTGTGTTCGTGTTCTACACATTTTTTGTCTAGTGCACTAAACATTGTCATATGGAATAAAAATCGCCACAAACGACTATGAGAAAAGATTGGTTCAGGAGCAGGGAATTTTTTTTCGAAAACGAAAATGAAAAATTTAGTTTGAAACGATATTTTGACCGAAATCGCACTATTTATGTTCACAATTTCCAGTGTGATTCTTTGGAAAGTTGTAAAATTTAAACATTTATAATTTTTCAGTATATCAGCATTTTTCGCTTAATTTTTTTTCCAAAAAATCGAAAAATAAATTTTCCACCAAATACACGAAAAATCTACAGCCCTGTTCAGGCATTTTCTCGATTTTTGCTCTAAAAATACGGTACTGGGTCTCGACACGAAAAGTTTTTATTAAATGCATGTGCGCCTTTAAAGAGTACTGTAATTTCAAACTCCCGTTTTTGCCGAATTTTTAAAACTGGTTTCCGATAAAAAATTGTCTGTTTATTCAAAAACAACTATAAAAGCACACCAATTTTAACAAATCGTAAGAAAAACTTTAAAAAATTGATTAAATTTCCGCAGCAACGAAATTTTGTAATTACAGTACTCTTCAAAGGCGCTCATCTATTTACATTAAATAAATATTGTCGTGTCGAGACCACGGACCGTATTTTTGGGGCGAAAATTGCAAAATTTTGCGTCTGGCGAATAGCCGTATTTTGTGATAATTTCCGTTTACCCGGTTGCAAATTGAAGAGGTCATACAGCAATGCACAGTAGGTGATCGTAAATATTCAAGTACCCGCCAACACCGCCAAAATGTTGTATGCACCCCCTCATAAACACCCTTGACAATACTACCATCGGTATTTATTTCACCTATATTGGCAGCAATCTACGAGTTTACCACCAACTATGTACATACCAGTTATTACATATTCGAATTTAAATGGTCCCTTGAATGGTTTGTCATTAATTATTGTAGATTTTTAAAGAAATCAGGCAATTTGATAAATTGACAAAAAAATGTAGATGTCGAGAACAATAATCACAATACAAATTCAAAAAAAGACTAACAACCAATCTACAGTAGTCTAGAAACAAAGAAATAAAATCGTACTTTTTTTATTCTTCAAGTTTCTTTCCGTGGATCCTTGAGCTTCTGTACTTTCAAAATTCTAGAACTTCCCGTCTCTCCCTATTCAATG*ATTTTTTGTCAAAGATTTTTCGGCGCCAGGTGTTTGGATTACTCATGTTAGGGCCGAG*TTCTTACCAATGTCTCAGGCATTTTTGGAAAAGTAATATTAAGAAAATTATACATATTTTCTTGAATACGAAAAATTTAAATGTTCGGTAAATCGATTCCTGAACGACTTCTAGTCGCATTTG
